# Addendum: An Optimised Dual Extraction Method for the Simultaneous and Accurate Analysis of Polar Metabolites and Lipids Carried out on Single Biological Samples. *Metabolites* 2020, *10*, 338

**DOI:** 10.3390/metabo10120490

**Published:** 2020-11-30

**Authors:** Joran Villaret-Cazadamont, Nathalie Poupin, Anthony Tournadre, Aurélie Batut, Lara Gales, Daniel Zalko, Nicolas J. Cabaton, Floriant Bellvert, Justine Bertrand-Michel

**Affiliations:** 1 Toxalim (Research Centre in Food Toxicology), Universite de Toulouse, INRAE, ENVT, INP-Purpan, UPS, 31027 Toulouse, France; joran.villaret-cazadamont@inrae.fr (J.V.-C.); nathalie.poupin@inrae.fr (N.P.); daniel.zalko@inrae.fr (D.Z.); nicolas.cabaton@inrae.fr (N.J.C.); 2 MetaboHUB-MetaToul-Lipidomics Core Facility, Inserm U1048, 31432 Toulouse, France; anthony.tournadre@sfr.fr (A.T.); aurelie_batut@orange.fr (A.B.); 3 MetaboHUB-MetaToul, National Infrastructure for Metabolomics and Fluxomics, 31077 Toulouse, France; lara.gales@insa-toulouse.fr; 4 Toulouse Biotechnology Institute, Université de Toulouse, CNRS, INRAE, INSA, 31400 Toulouse, France

The authors wish to make the following comment to the paper [1]:

Some words were unfortunately cut in the Material & Method Section 3.5. Double Extraction Method which prevents the protocol from being carried out correctly. The addition of 1 mL of water disappeared from the text; this step is crucial for a good realization of our new protocole.

So please use this completed protocole to perform the double extraction: Polar metabolites and lipids were extracted **using 4 mL of** a quenching solution consisting of a mix of cold (−20 °C) acetonitrile, methanol, and milliQ water with 0.1% formic acid 2:2:1 (*v*/*v*/*v*). Internal standards were added for polar metabolites and lipids using the same volume as described for the classical extraction. Samples were centrifuged at 400× *g*, polar metabolites and lipids were separated using a Bligh and Dyer modified extraction with 2.5 mL of dichloromethane **and 1 mL of water**. The aqueous phase (upper phase) was kept, evaporated, and resuspended in 100 µL of milliQ water for metabolites analysis. The organic phase (lower phase) was treated in the same way as described for the classical extraction procedure for apolar metabolites.

The authors would like to apologize for this omission, which compromises the success of the protocol described in this manuscript. These comments do not affect the scientific results.
