# Supplementary material for: An Optimized Dual Extraction Method for the Simultaneous and Accurate Analysis of Polar Metabolites and Lipids Carried out on Single Biological Samples
Source: Metabolites. 2020 Aug 19;10(9):338. doi: 10.3390/metabo10090338 (PMC7570216; doi:10.3390/metabo10090338)
Supplement: Supplementary file 1 [file metabolites-10-00338-s001.zip › Figure S1.pdf]

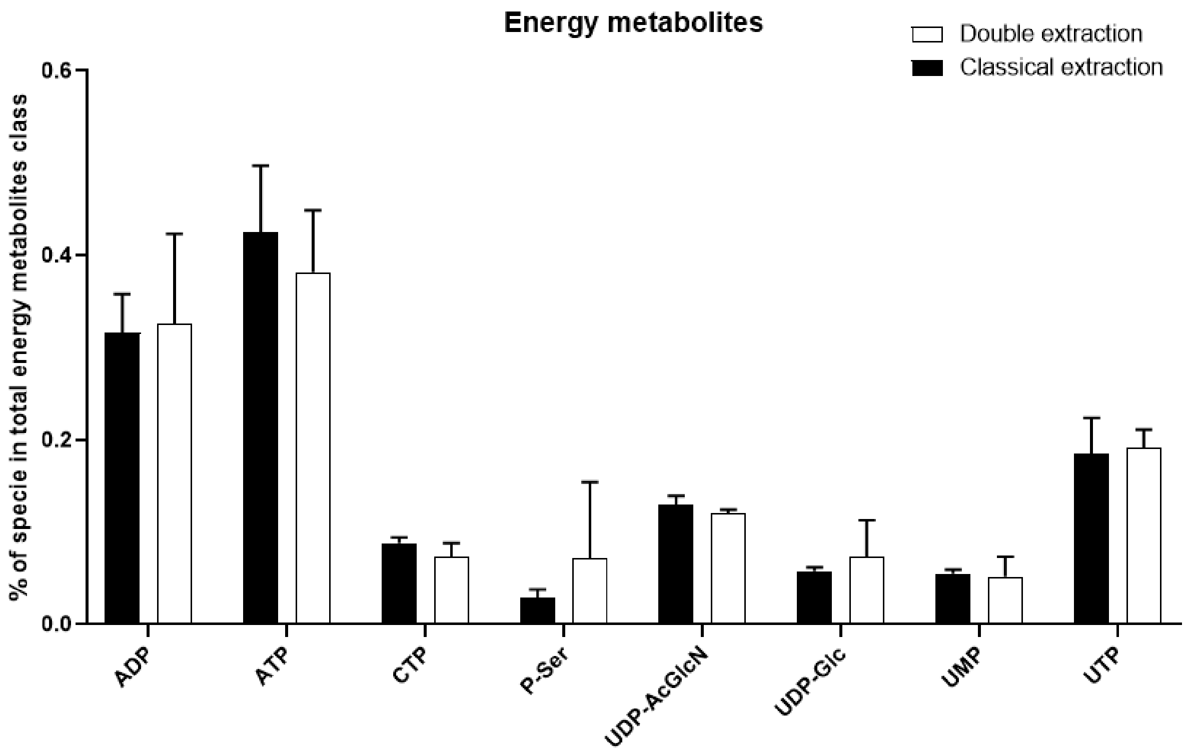

Energy metabolites
Double extraction
Classical extraction
% of specie in total energy metabolites class
0.6
0.4
0.2
0.0
ADP
ATP
CTP
P-Ser
UDP-AcGlcN
UDP-Glc
UMP
UTP

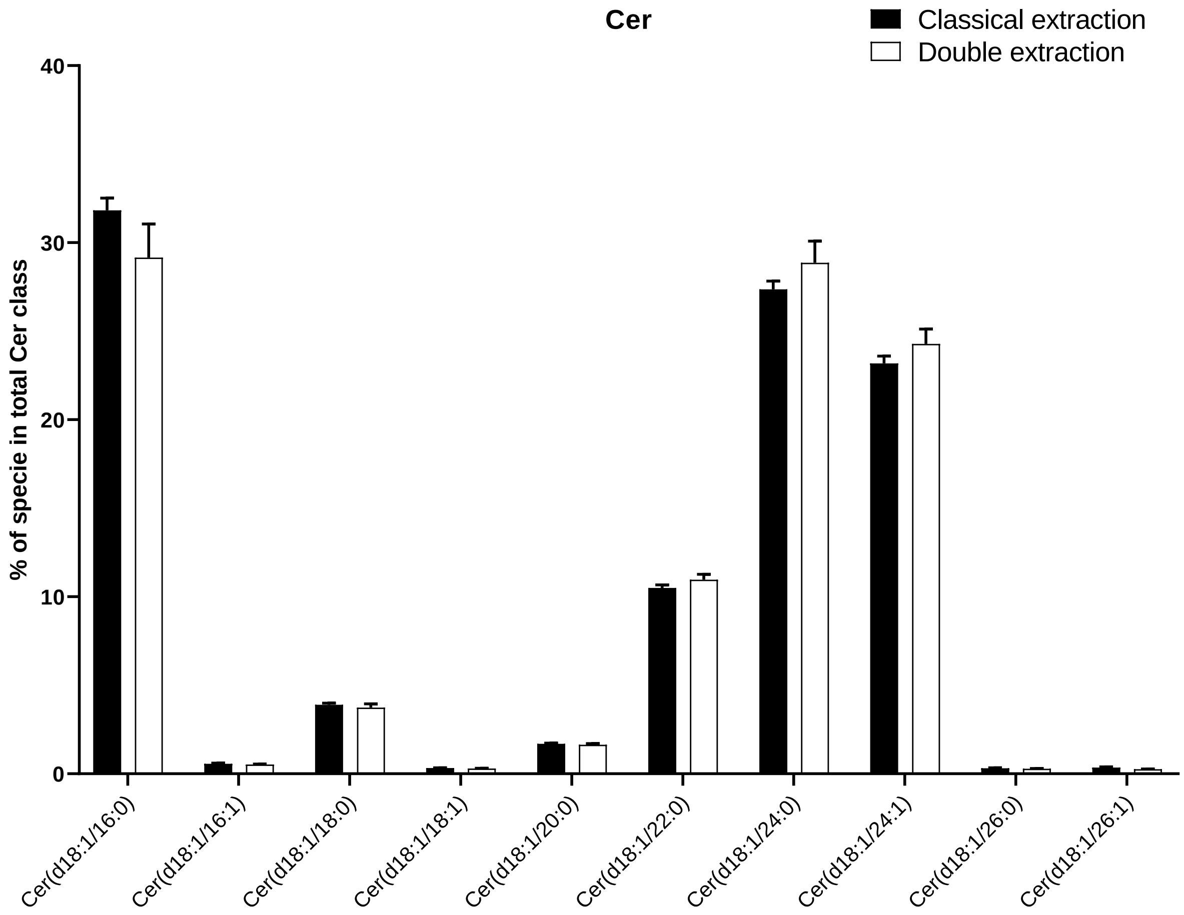

Cer
Classical extraction
Double extraction
% of specie in total Cer class
40
30
20
10
0
Cer(d18:1/16:0)
Cer(d18:1/16:1)
Cer(d18:1/18:0)
Cer(d18:1/18:1)
Cer(d18:1/20:0)
Cer(d18:1/22:0)
Cer(d18:1/24:0)
Cer(d18:1/24:1)
Cer(d18:1/26:0)
Cer(d18:1/26:1)

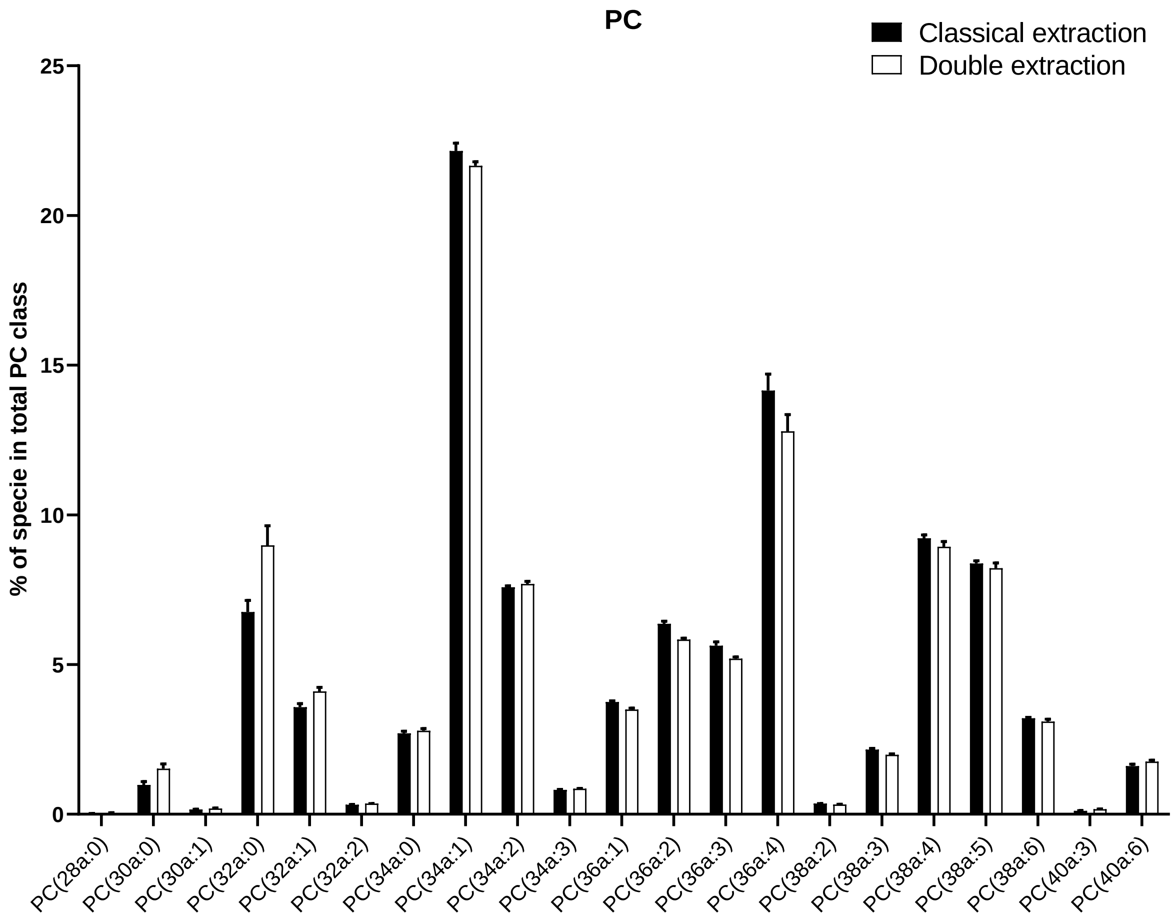

PC
Classical extraction
Double extraction
% of specie in total PC class
25
20
15
10
5
0
PC(28a:0)
PC(30a:0)
PC(30a:1)
PC(32a:0)
PC(32a:1)
PC(32a:2)
PC(34a:0)
PC(34a:1)
PC(34a:2)
PC(34a:3)
PC(36a:1)
PC(36a:2)
PC(36a:3)
PC(36a:4)
PC(38a:2)
PC(38a:3)
PC(38a:4)
PC(38a:5)
PC(38a:6)
PC(40a:3)
PC(40a:6)

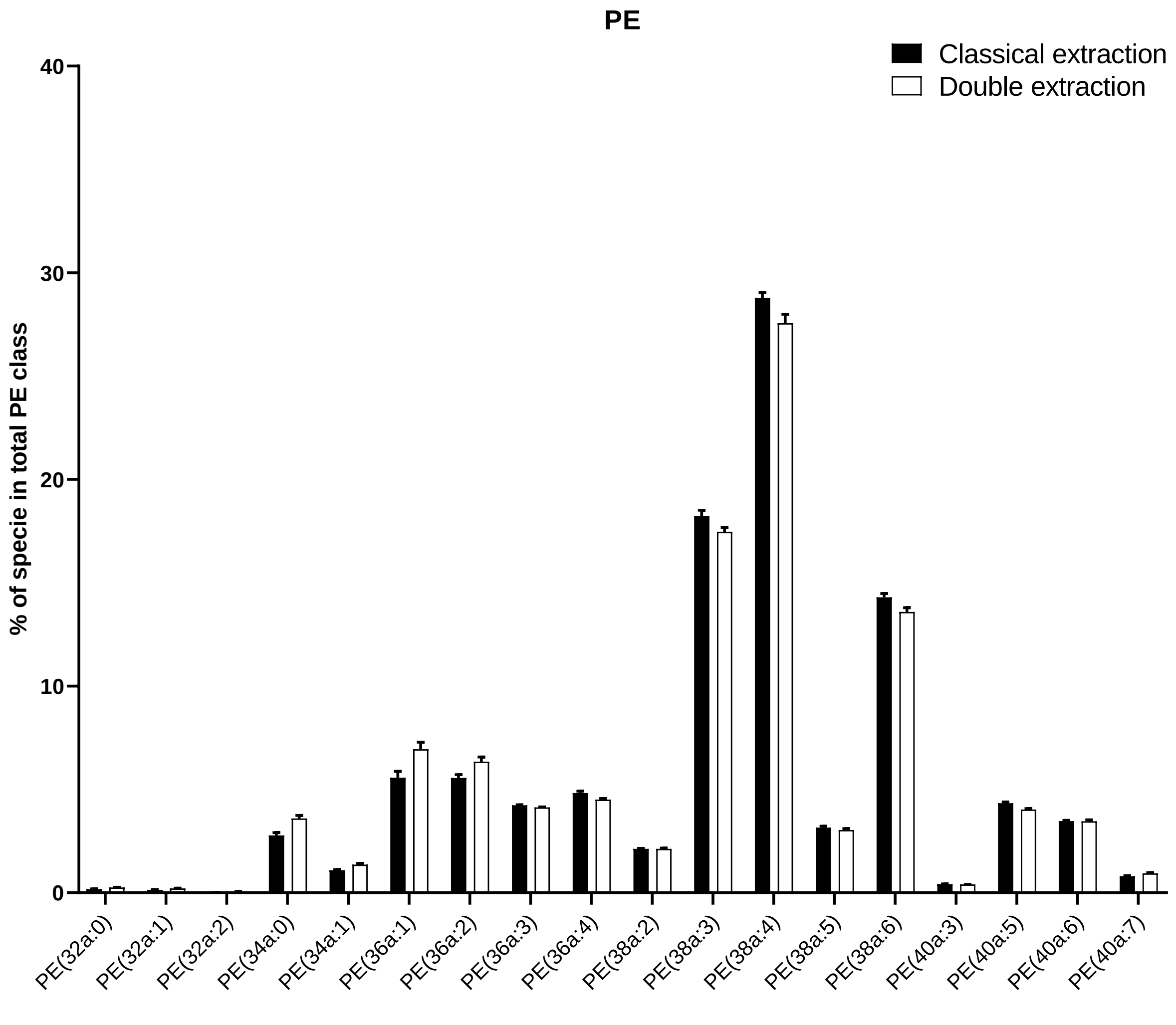
PE
Classical extraction
Double extraction
% of specie in total PE class
40
30
20
10
0
PE(32a:0)
PE(32a:1)
PE(32a:2)
PE(34a:0)
PE(34a:1)
PE(36a:1)
PE(36a:2)
PE(36a:3)
PE(36a:4)
PE(38a:2)
PE(38a:3)
PE(38a:4)
PE(38a:5)
PE(38a:6)
PE(40a:3)
PE(40a:5)
PE(40a:6)
PE(40a:7)

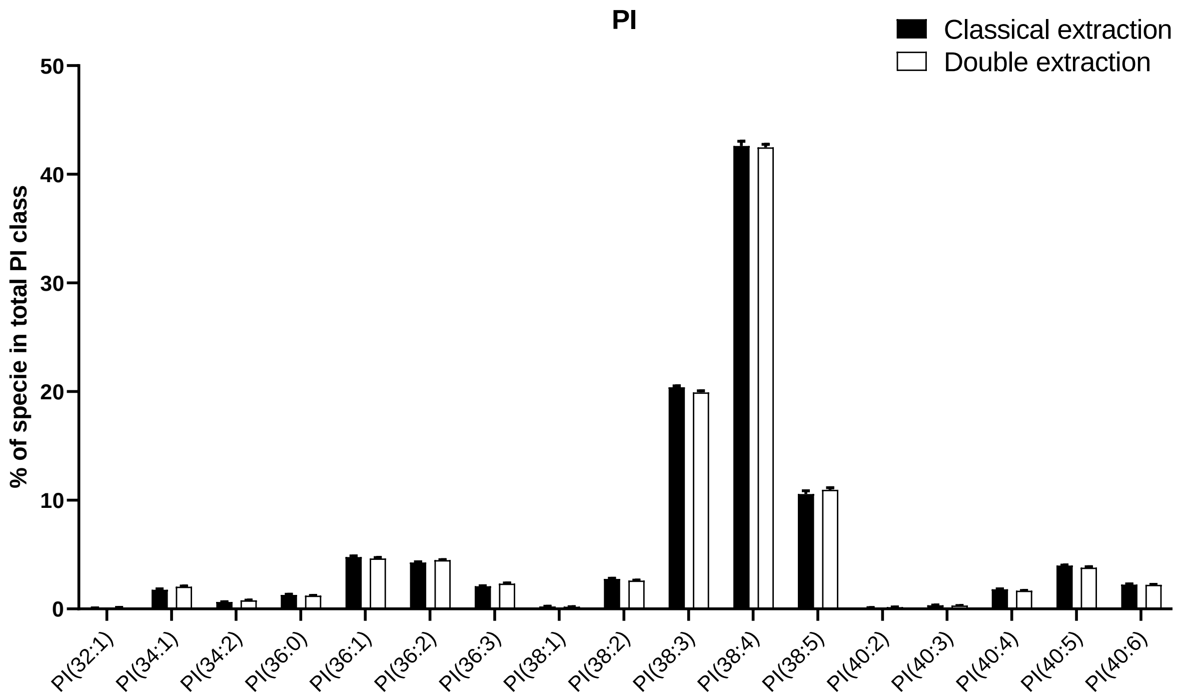

Figure S1. Comparison of the qualitative distribution of Ceramides (Cer), phosphocholine (PC), phosphatidylethanolamine (PE), phosphoinositols (PI) and energy metabolites species between classical and double extraction methods. Each species is represented as a proportion (%) of its total class. Total lipids classes are considered as 100%. Bars represent mean ± SD (n=5). A Khi2 ($\chi 2$) test was performed separately for each class to compare the relative distribution between both methods (all tests were non-significant).
