# Supplementary material for: An Optimized Dual Extraction Method for the Simultaneous and Accurate Analysis of Polar Metabolites and Lipids Carried out on Single Biological Samples
Source: Metabolites. 2020 Aug 19;10(9):338. doi: 10.3390/metabo10090338 (PMC7570216; doi:10.3390/metabo10090338)
Supplement: Supplementary file 1 [file metabolites-10-00338-s001.zip › Figure S2.pdf]

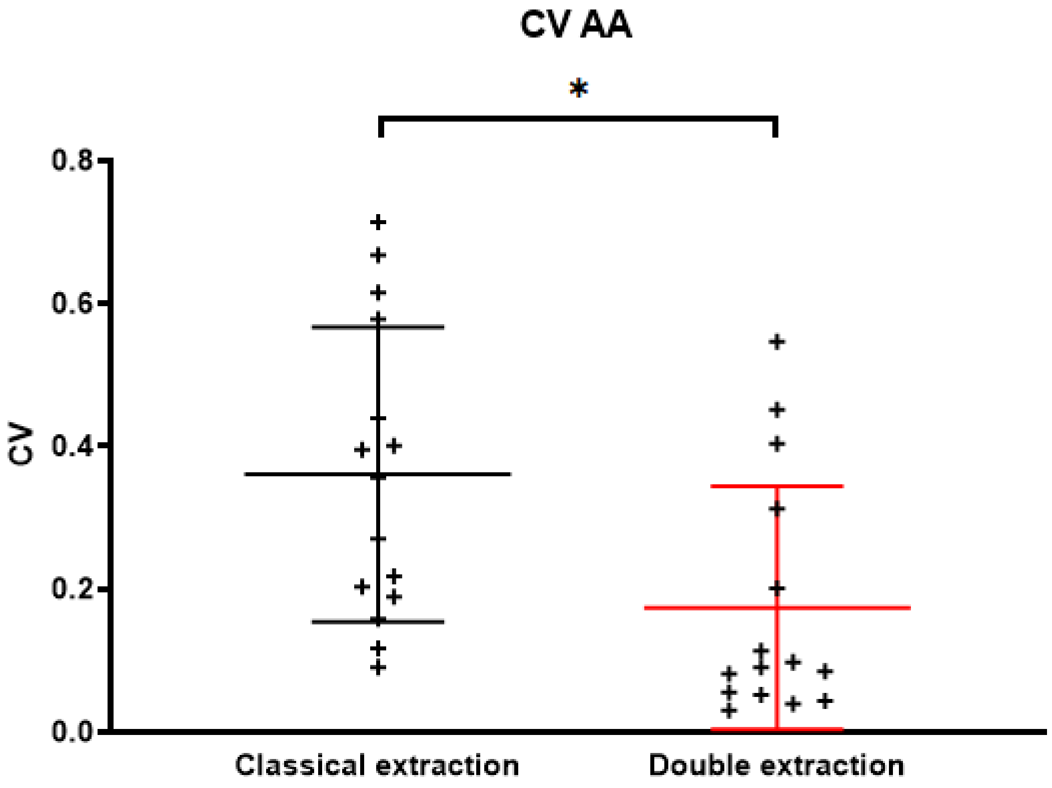
CV AA
*
0.8
0.6
0.4
0.2
0.0
CV
Classical extraction
Double extraction

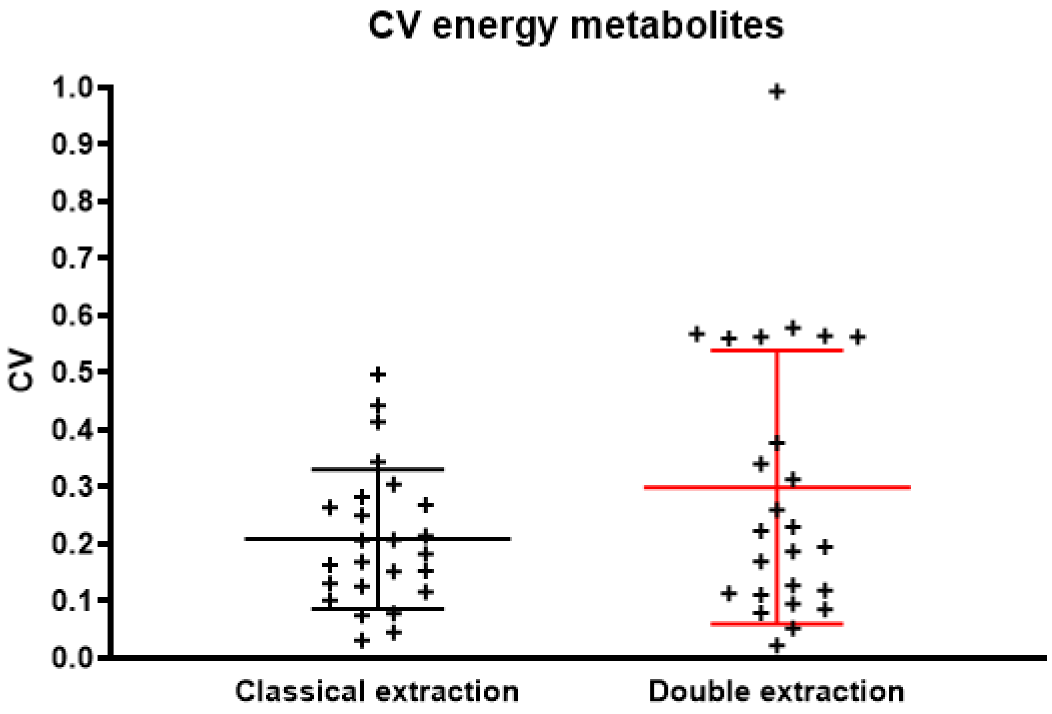
CV energy metabolites
1.0
0.9
0.8
0.7
0.6
0.5
0.4
0.3
0.2
0.1
0.0
CV
Classical extraction
Double extraction

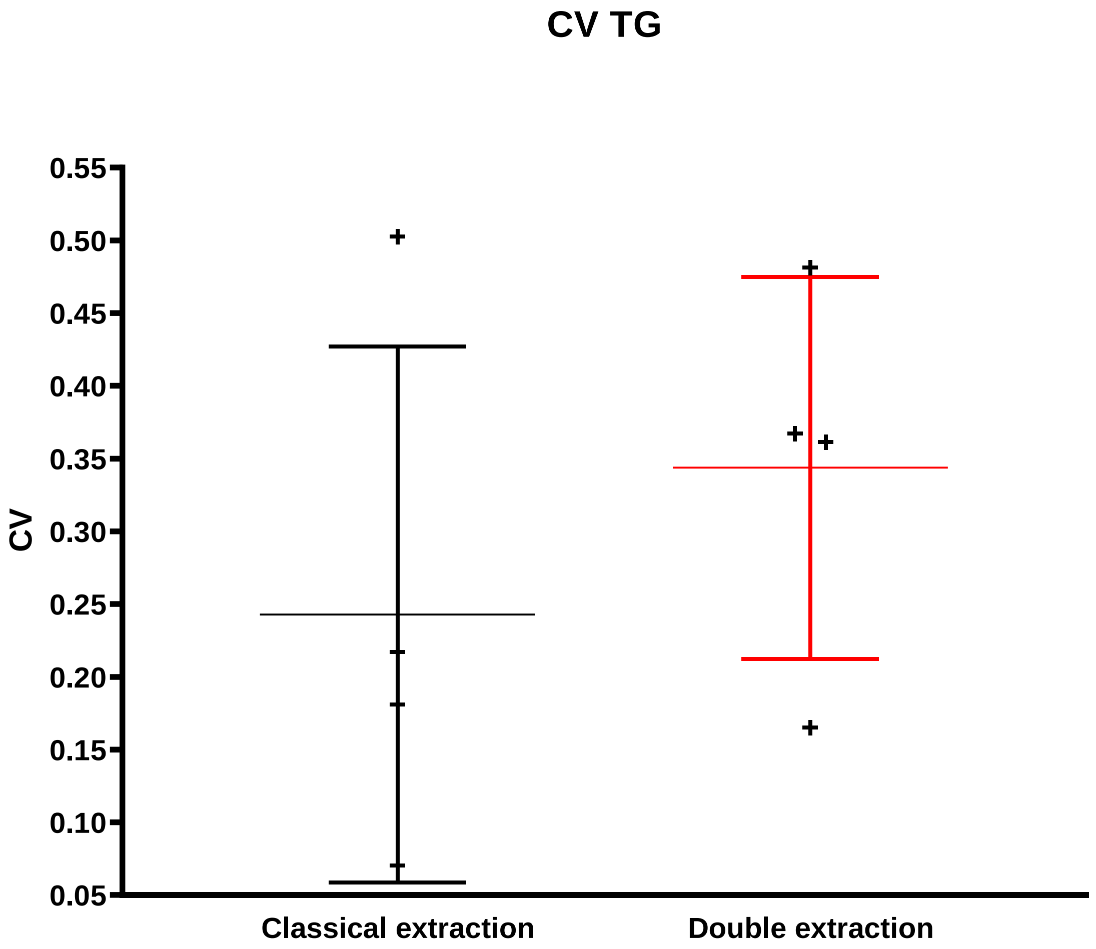
CV TG
CV
0.55
0.50
0.45
0.40
0.35
0.30
0.25
0.20
0.15
0.10
0.05
Classical extraction
Double extraction

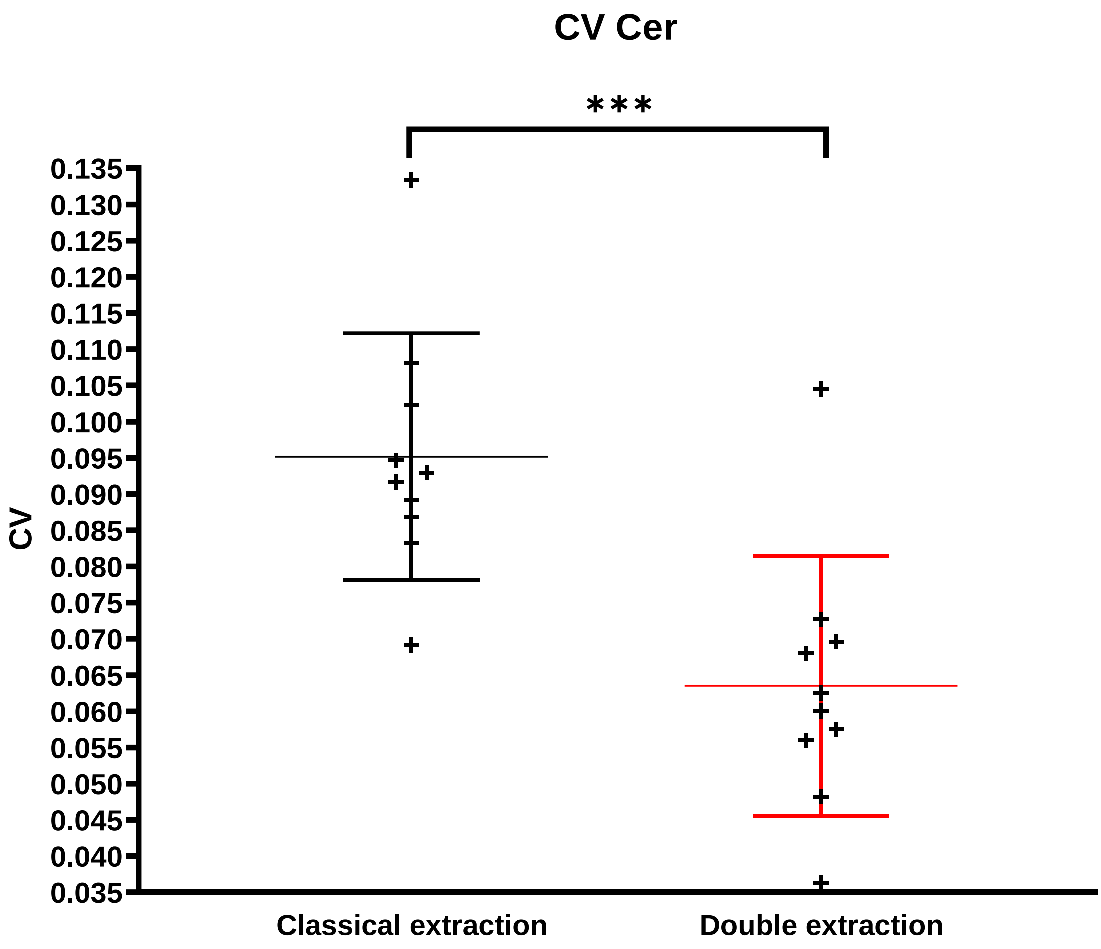
CV Cer
***
0.135
0.130
0.125
0.120
0.115
0.110
0.105
0.100
0.095
0.090
0.085
0.080
0.075
0.070
0.065
0.060
0.055
0.050
0.045
0.040
0.035
CV
Classical extraction
Double extraction

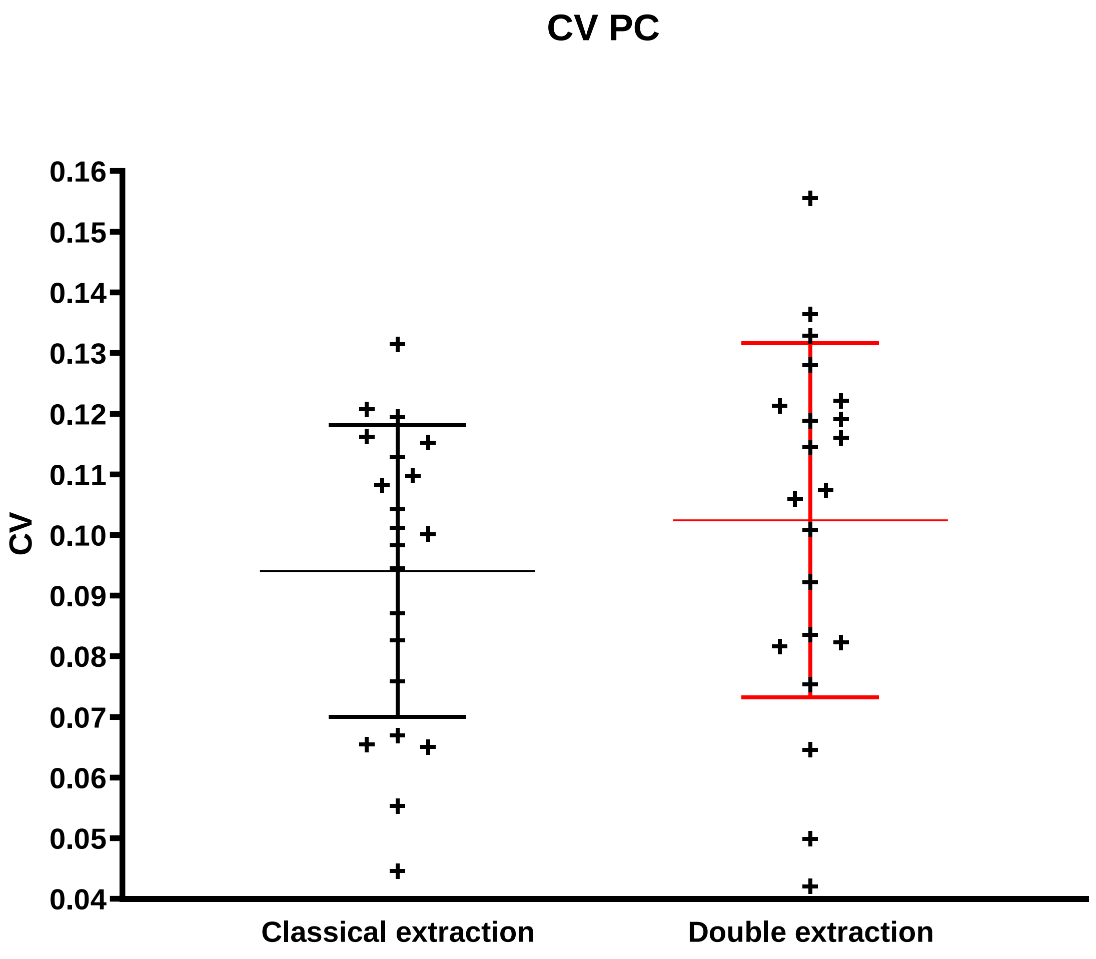
CV PC
CV
0.16
0.15
0.14
0.13
0.12
0.11
0.10
0.09
0.08
0.07
0.06
0.05
0.04
Classical extraction
Double extraction

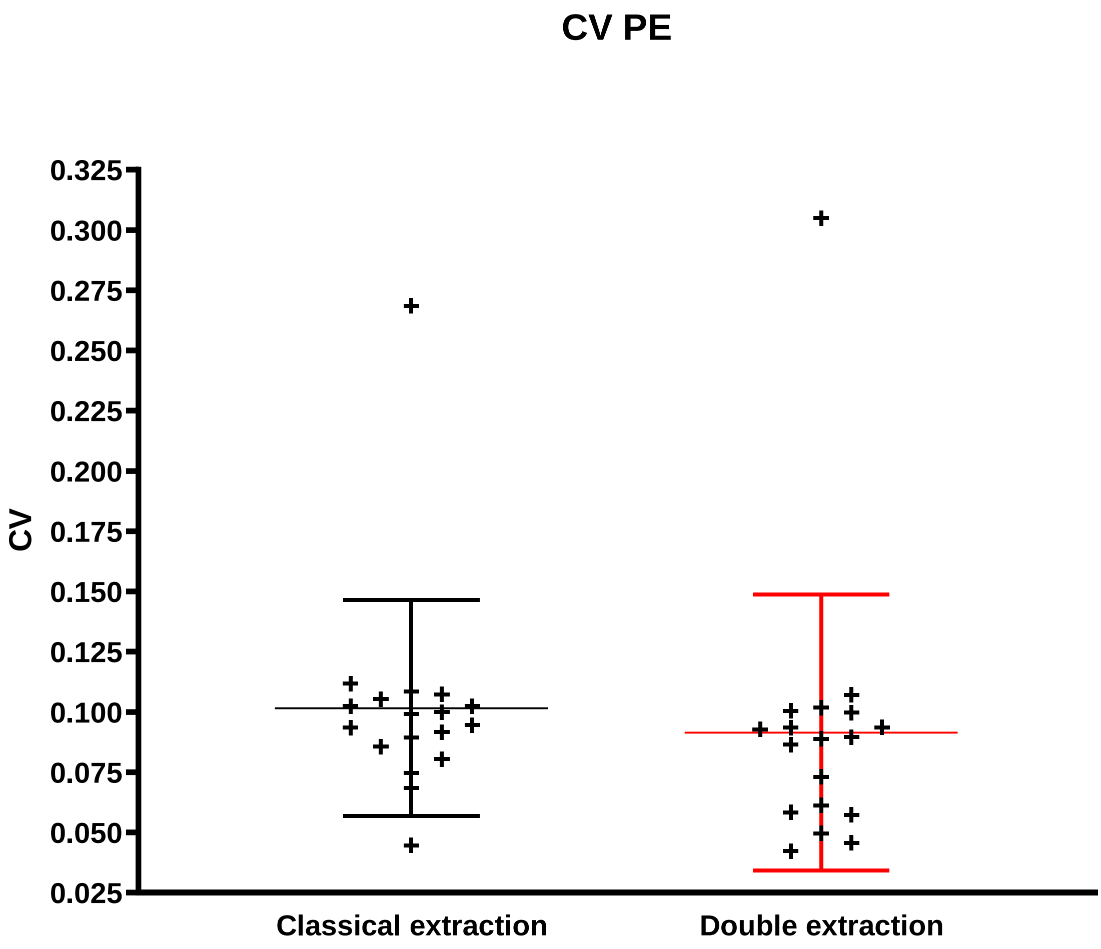
CV PE
CV
0.325
0.300
0.275
0.250
0.225
0.200
0.175
0.150
0.125
0.100
0.075
0.050
0.025
Classical extraction
Double extraction

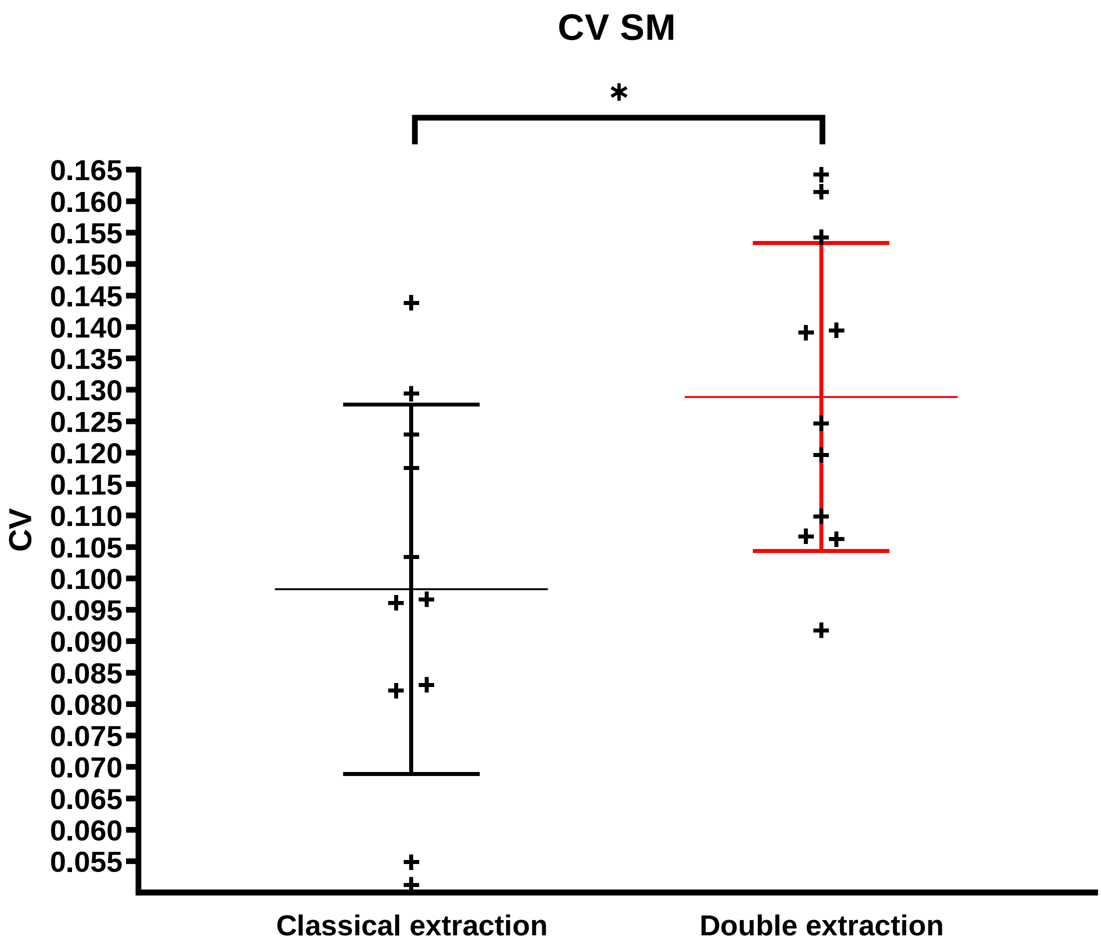

CV SM
*
0.165
0.160
0.155
0.150
0.145
0.140
0.135
0.130
0.125
0.120
0.115
0.110
0.105
0.100
0.095
0.090
0.085
0.080
0.075
0.070
0.065
0.060
0.055
CV
Classical extraction
Double extraction

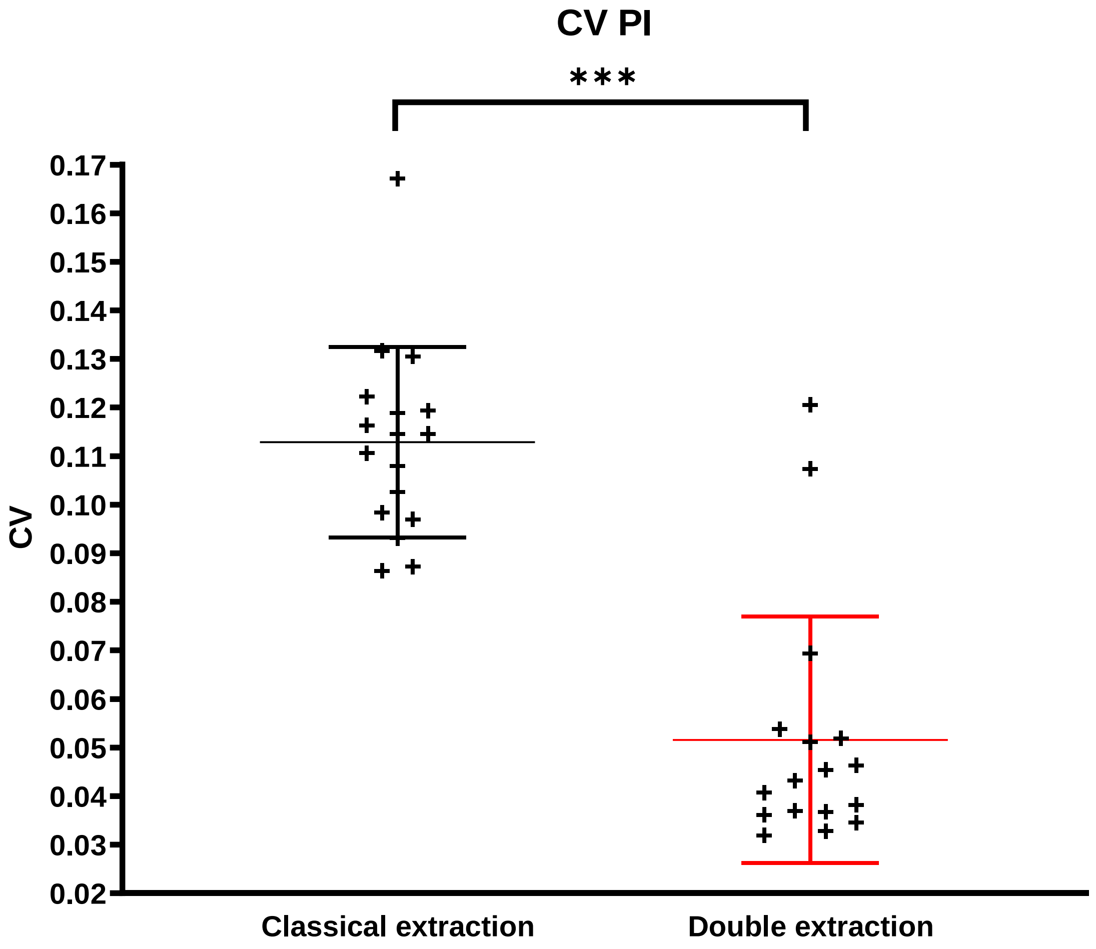

Figure S2. Comparison of the coefficients of variations (CV) obtained for amino acids (AA), energy metabolites, triglycerides (TG), ceramides (Cer), phosphocholine (PC), phosphatidylethanolamine (PE), sphingomyelins (SM) and phosphoinositols (PI) between classical and double extraction methods. Each point represent CV obtained for a specific molecule (n=5). Bars represent mean ± SD. t-tests were performed for each class of metabolites and lipids (* p < 0.05; ** p < 0.01; *** p < 0.001).
