# Supplementary material for: An Optimized Dual Extraction Method for the Simultaneous and Accurate Analysis of Polar Metabolites and Lipids Carried out on Single Biological Samples
Source: Metabolites. 2020 Aug 19;10(9):338. doi: 10.3390/metabo10090338 (PMC7570216; doi:10.3390/metabo10090338)
Supplement: Supplementary file 1 [file metabolites-10-00338-s001.zip › Figure S3.pdf]

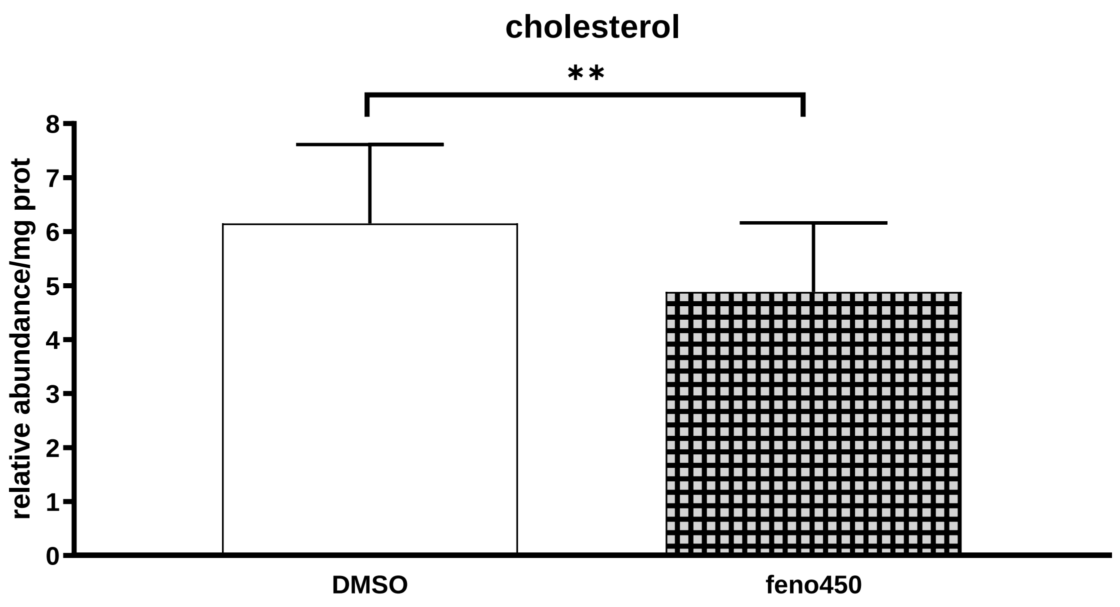
cholesterol
**
relative abundance/mg prot
0
1
2
3
4
5
6
7
8
DMSO
feno450

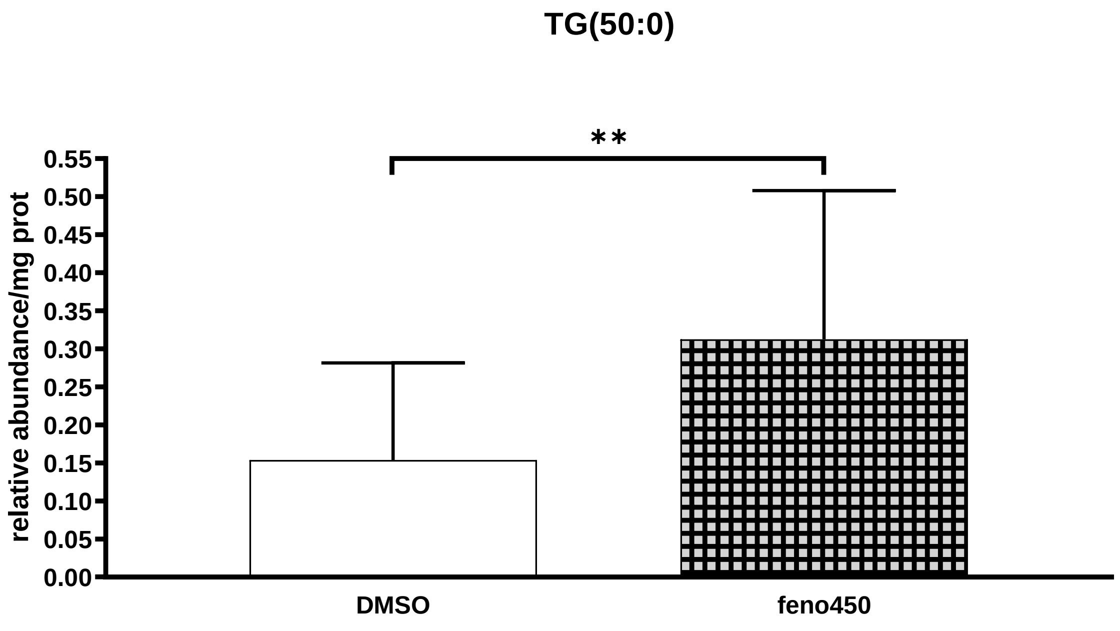
TG(50:0)
**
relative abundance/mg prot
0.00
0.05
0.10
0.15
0.20
0.25
0.30
0.35
0.40
0.45
0.50
0.55
DMSO
feno450

TG(52:0)

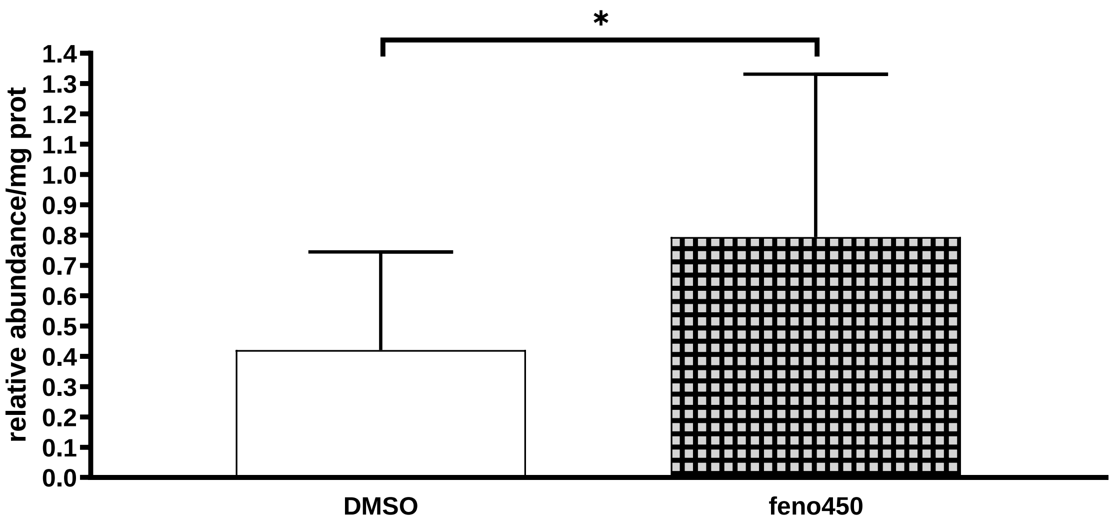
*
relative abundance/mg prot
1.4
1.3
1.2
1.1
1.0
0.9
0.8
0.7
0.6
0.5
0.4
0.3
0.2
0.1
0.0
DMSO
feno450

TG(54:0)

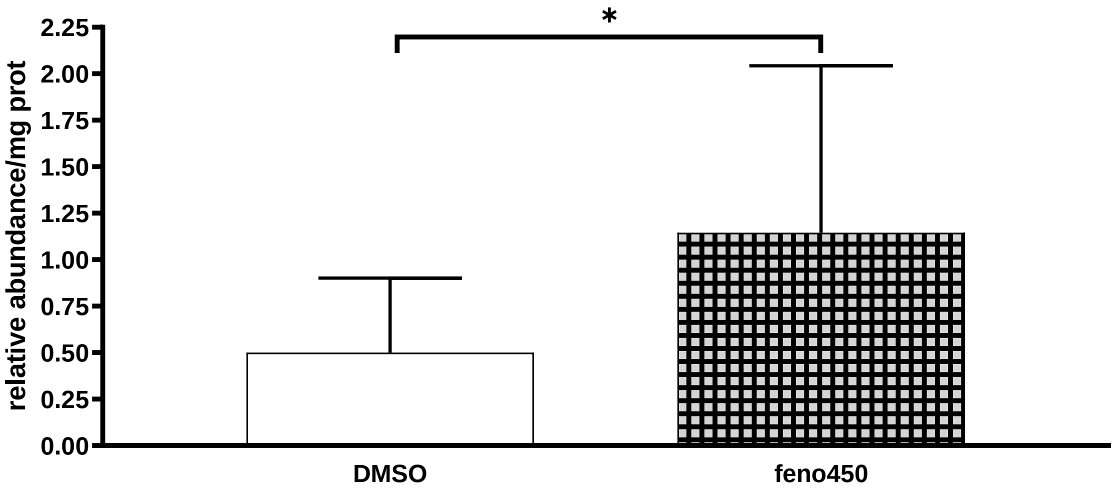
*
relative abundance/mg prot
2.25
2.00
1.75
1.50
1.25
1.00
0.75
0.50
0.25
0.00
DMSO
feno450

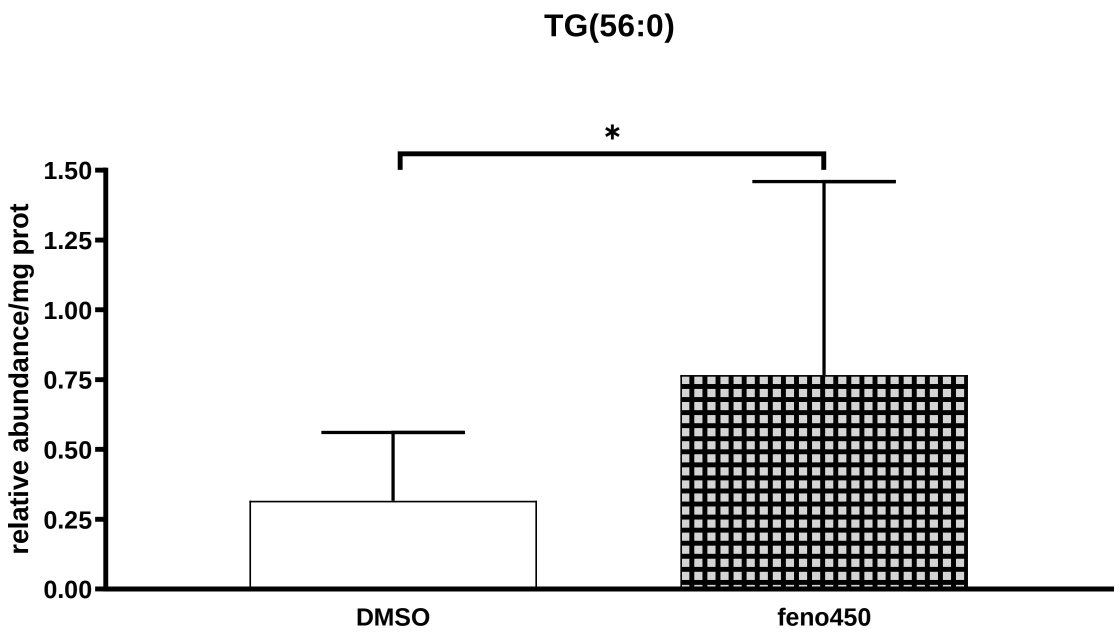
TG(56:0)
*
relative abundance/mg prot
1.50
1.25
1.00
0.75
0.50
0.25
0.00
DMSO
feno450

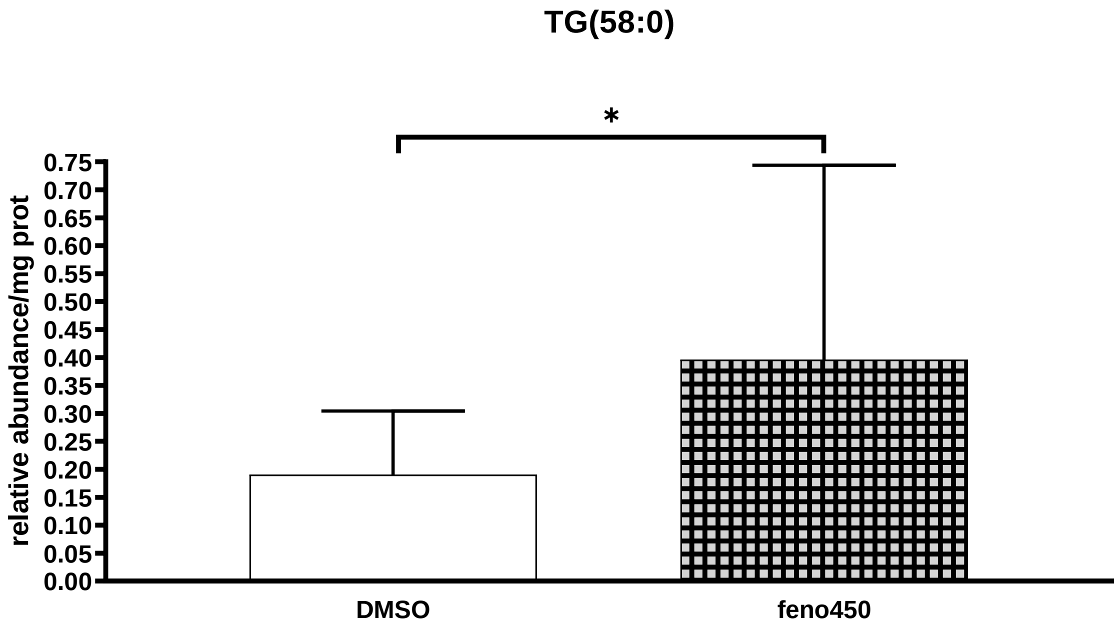
TG(58:0)
*
relative abundance/mg prot
0.75
0.70
0.65
0.60
0.55
0.50
0.45
0.40
0.35
0.30
0.25
0.20
0.15
0.10
0.05
0.00
DMSO
feno450

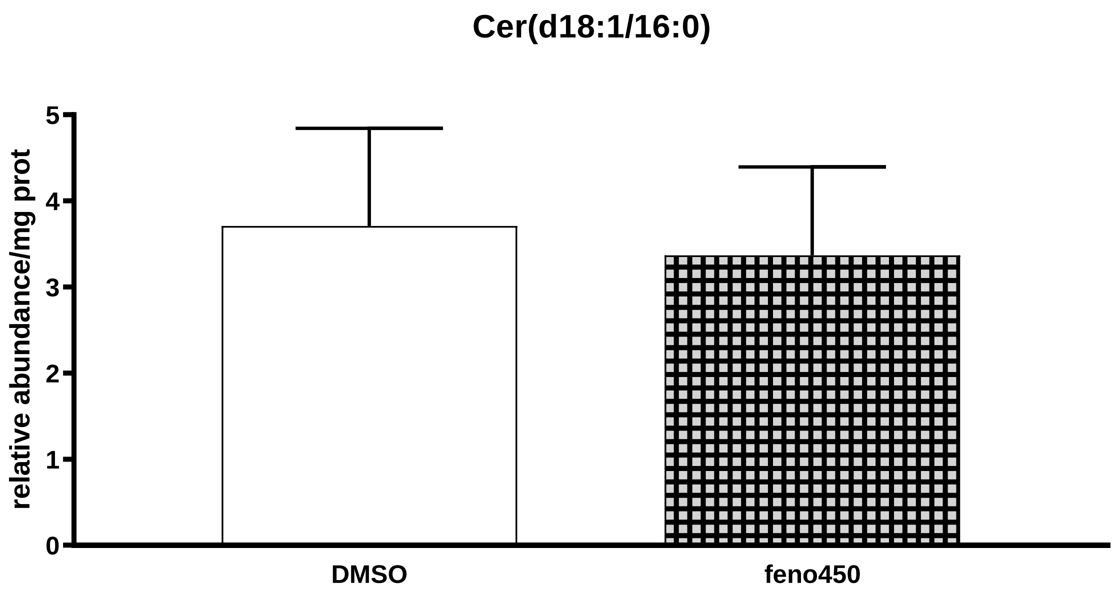
Cer(d18:1/16:0)
relative abundance/mg prot
0
1
2
3
4
5
DMSO
feno450

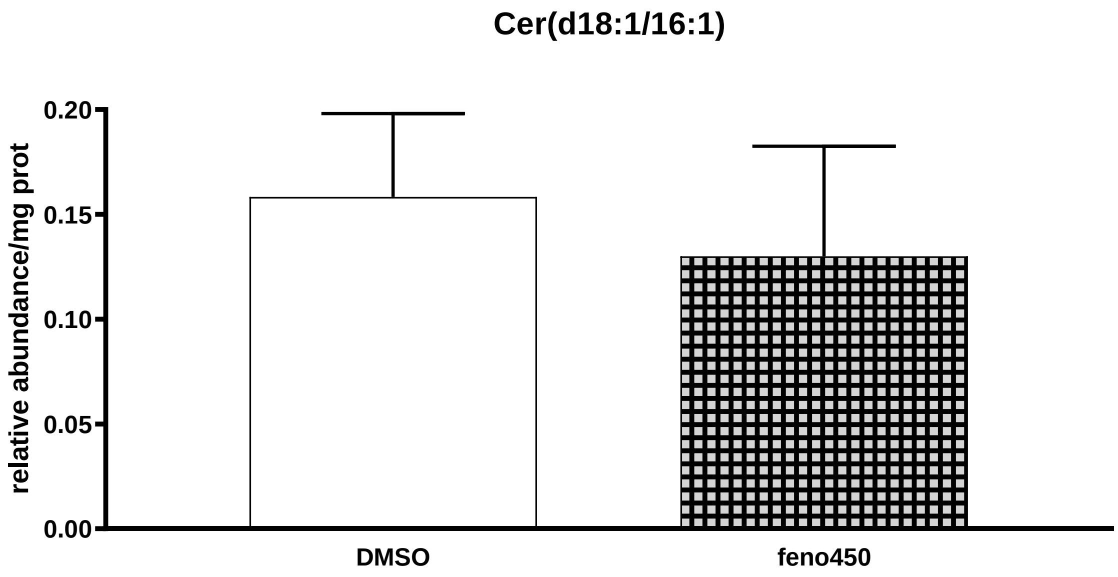
Cer(d18:1/16:1)
relative abundance/mg prot
0.00
0.05
0.10
0.15
0.20
DMSO
feno450

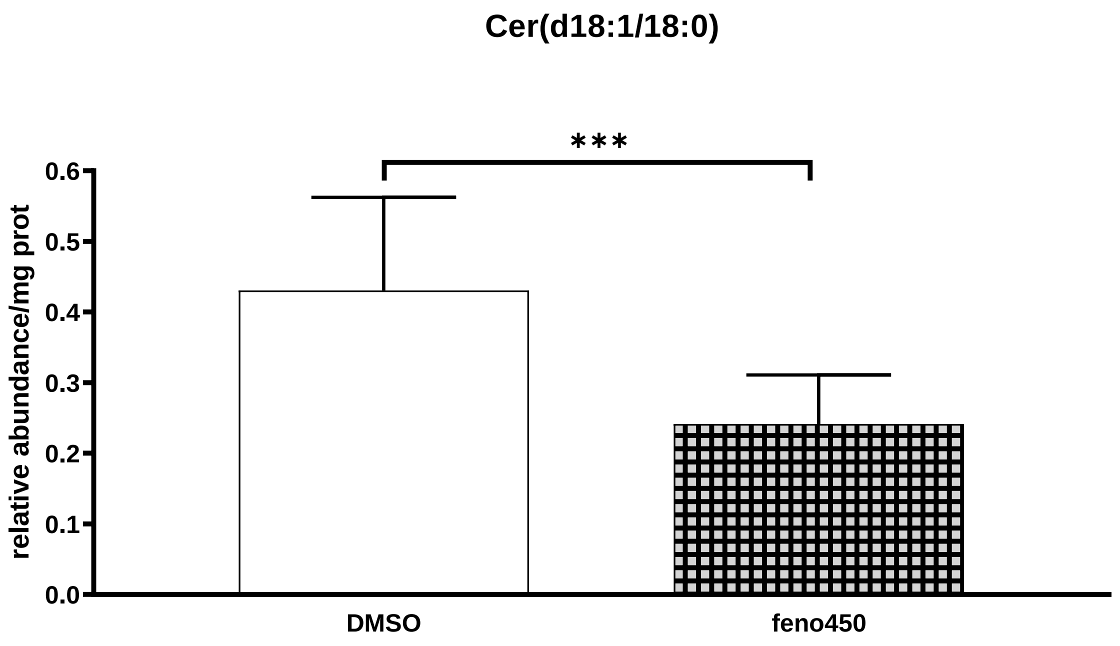
Cer(d18:1/18:0)
***
relative abundance/mg prot
0.6
0.5
0.4
0.3
0.2
0.1
0.0
DMSO
feno450

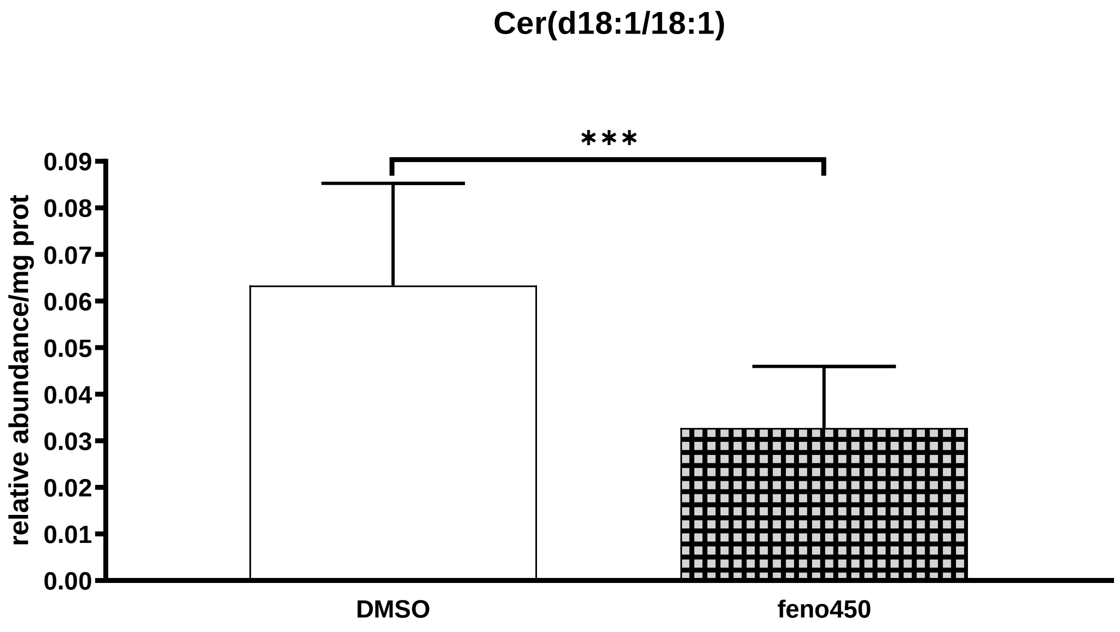
Cer(d18:1/18:1)
***
relative abundance/mg prot
0.09
0.08
0.07
0.06
0.05
0.04
0.03
0.02
0.01
0.00
DMSO
feno450

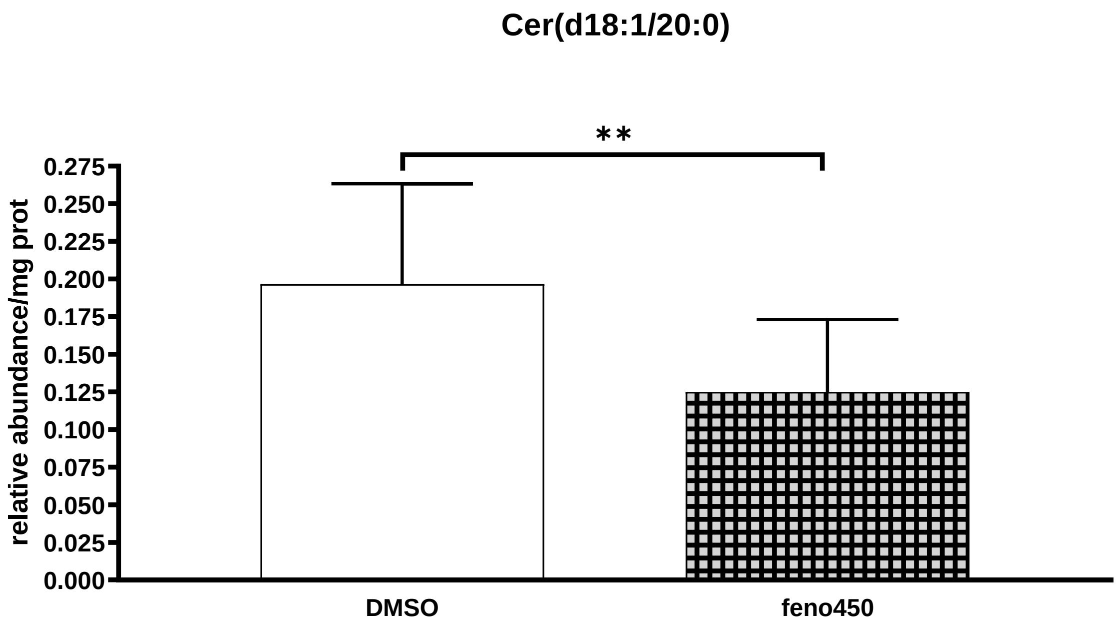
Cer(d18:1/20:0)
**
relative abundance/mg prot
0.275
0.250
0.225
0.200
0.175
0.150
0.125
0.100
0.075
0.050
0.025
0.000
DMSO
feno450

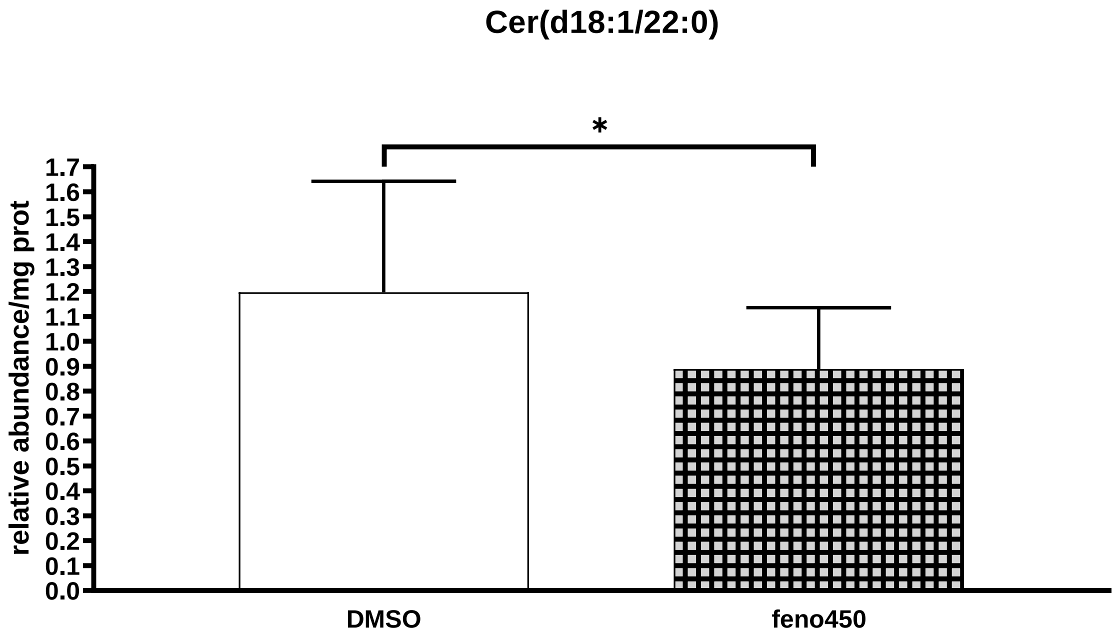
Cer(d18:1/22:0)
*
relative abundance/mg prot
1.7
1.6
1.5
1.4
1.3
1.2
1.1
1.0
0.9
0.8
0.7
0.6
0.5
0.4
0.3
0.2
0.1
0.0
DMSO
feno450

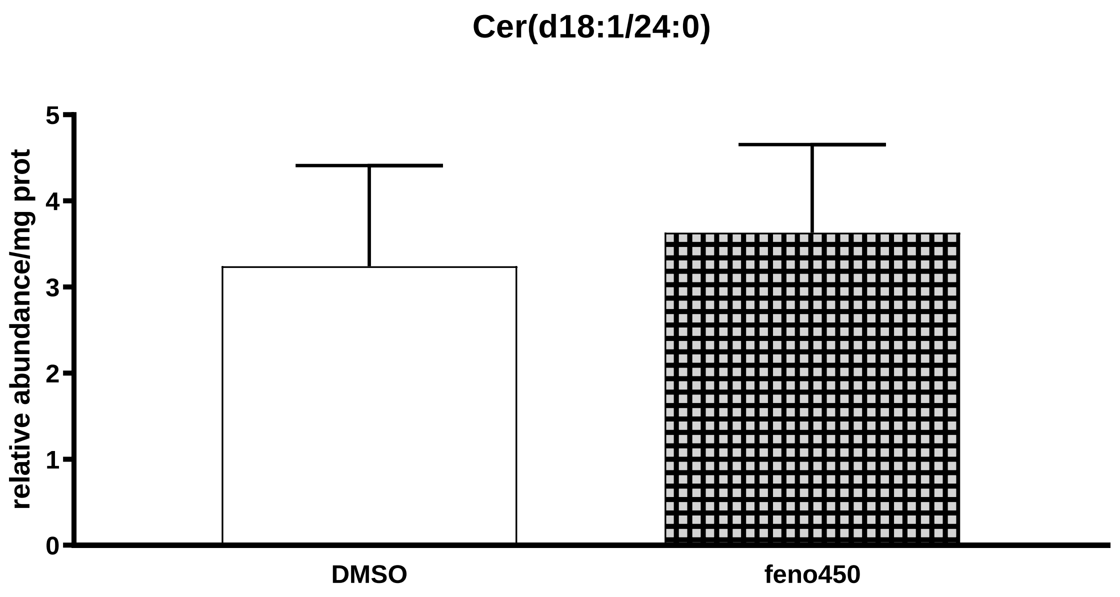
Cer(d18:1/24:0)
relative abundance/mg prot
0
1
2
3
4
5
DMSO
feno450

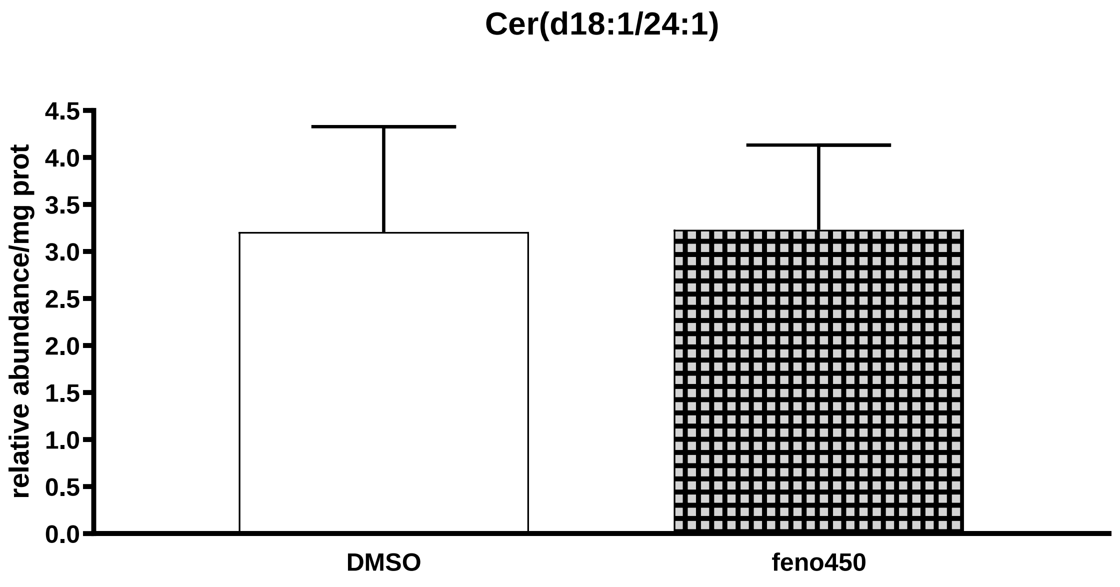
Cer(d18:1/24:1)
relative abundance/mg prot
0.0
0.5
1.0
1.5
2.0
2.5
3.0
3.5
4.0
4.5
DMSO
feno450

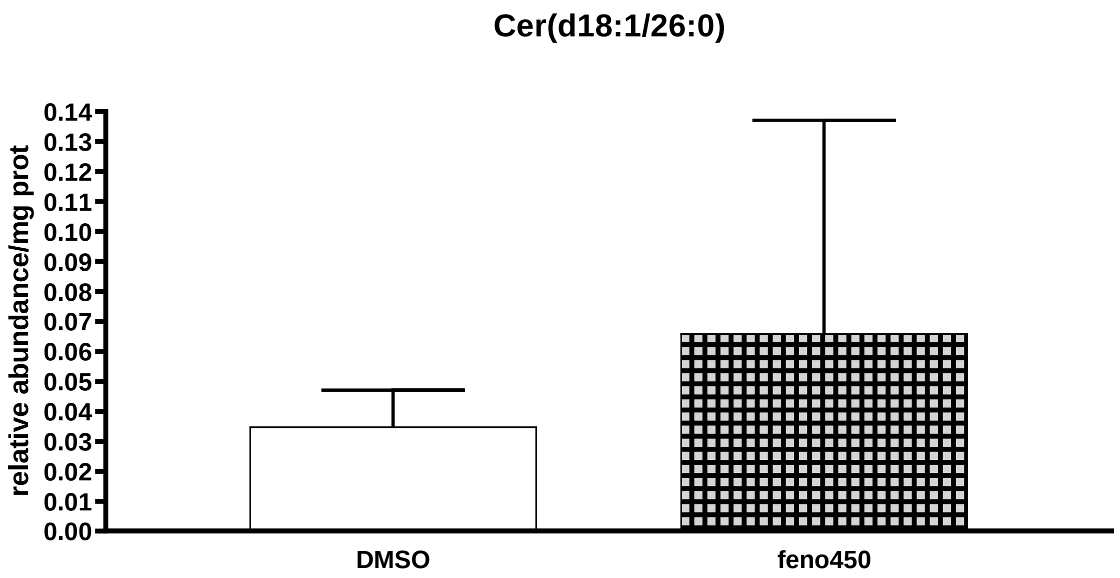
Cer(d18:1/26:0)
relative abundance/mg prot
0.14
0.13
0.12
0.11
0.10
0.09
0.08
0.07
0.06
0.05
0.04
0.03
0.02
0.01
0.00
DMSO
feno450

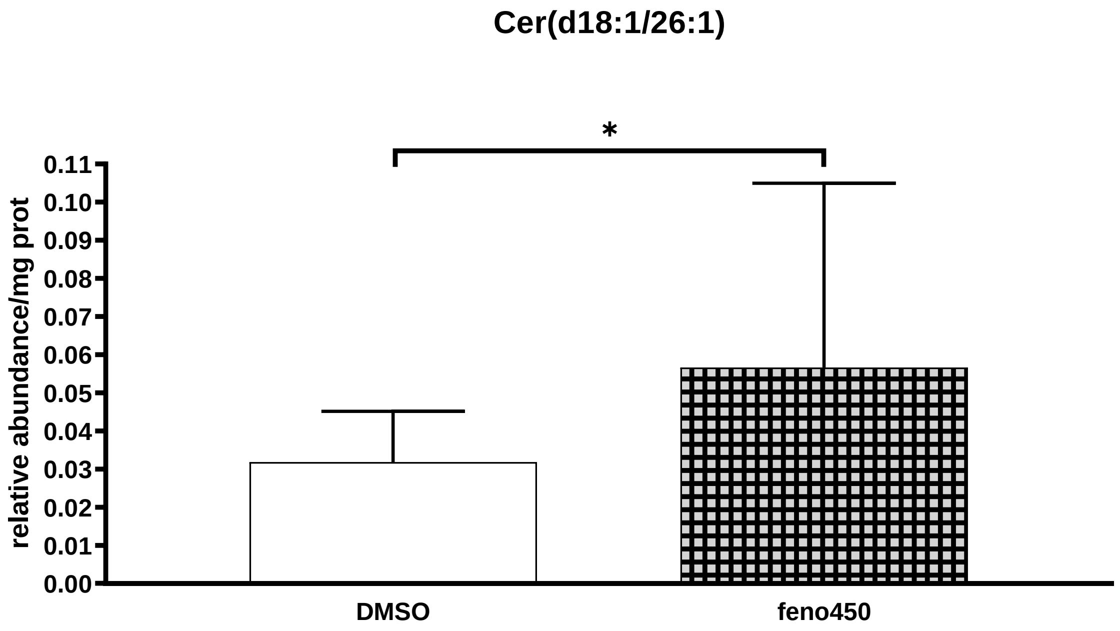
Cer(d18:1/26:1)
*
relative abundance/mg prot
0.11
0.10
0.09
0.08
0.07
0.06
0.05
0.04
0.03
0.02
0.01
0.00
DMSO
feno450

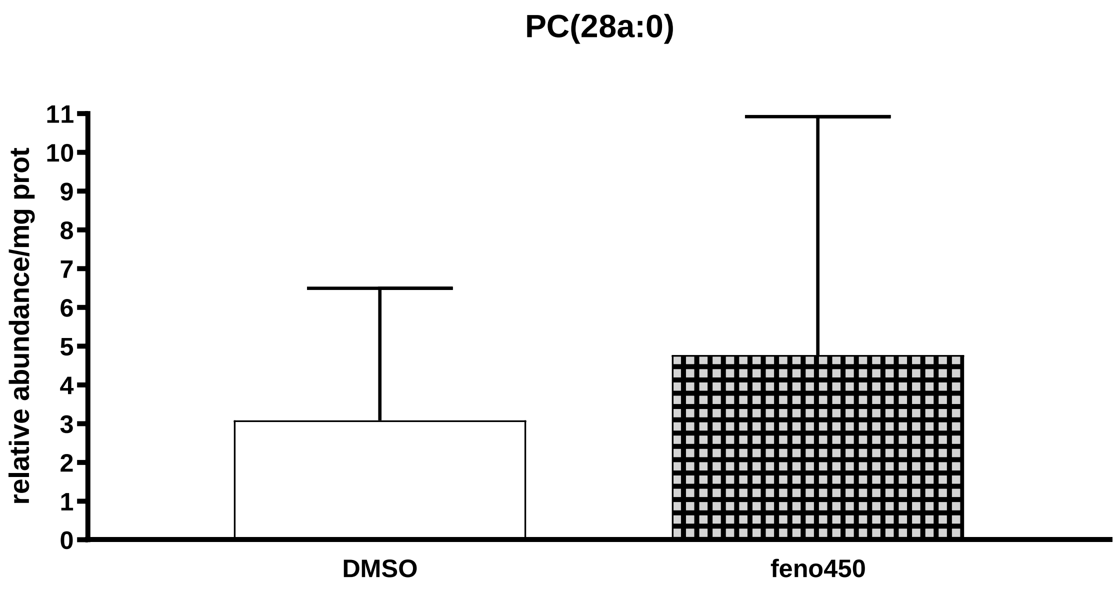
PC(28a:0)
relative abundance/mg prot
11
10
9
8
7
6
5
4
3
2
1
0
DMSO
feno450

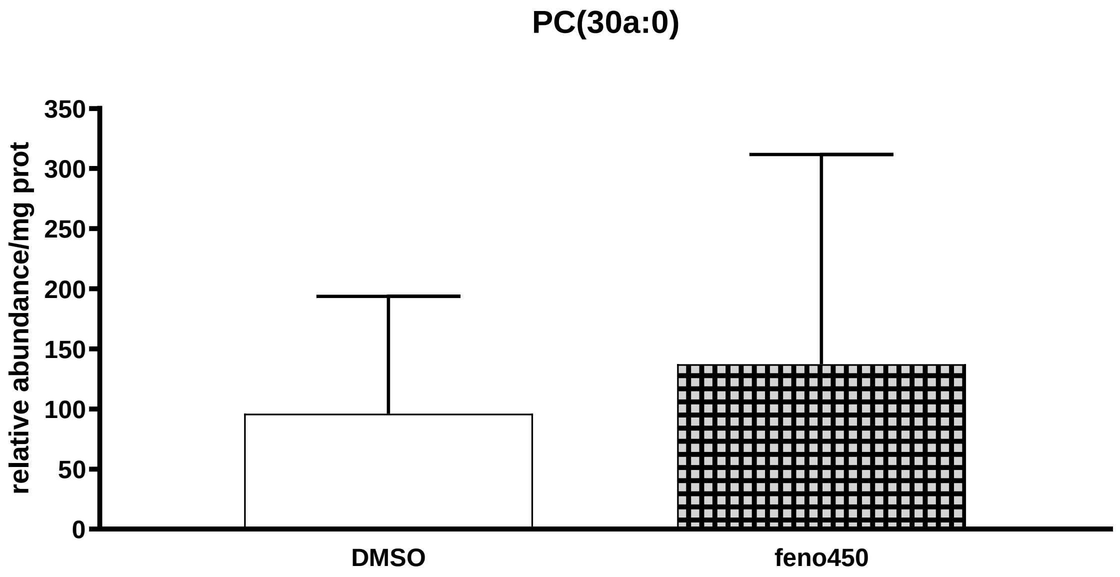
PC(30a:0)
relative abundance/mg prot
350
300
250
200
150
100
50
0
DMSO
feno450

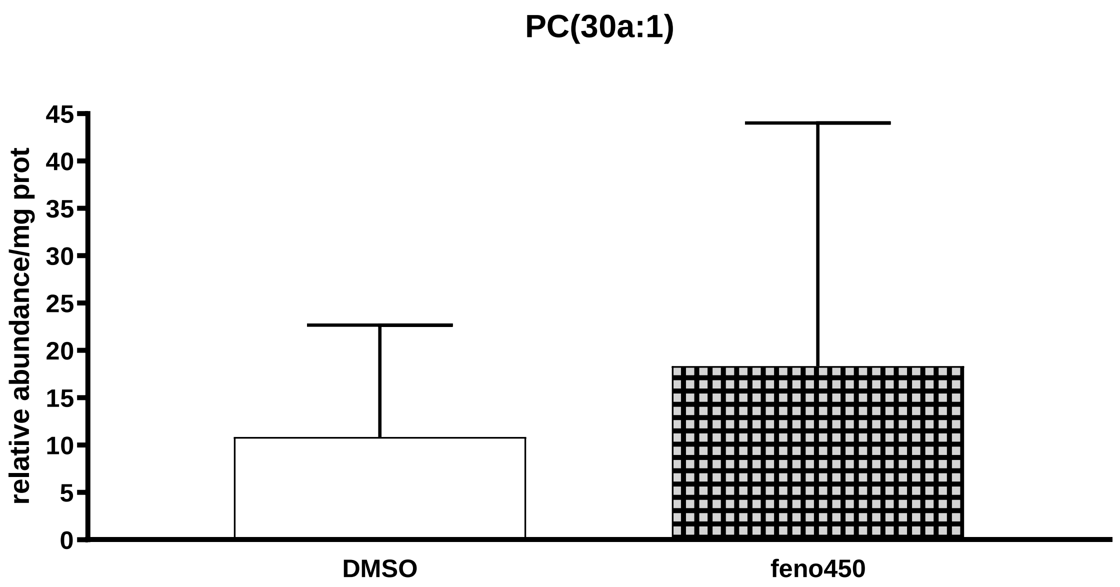
PC(30a:1)
relative abundance/mg prot
45
40
35
30
25
20
15
10
5
0
DMSO
feno450

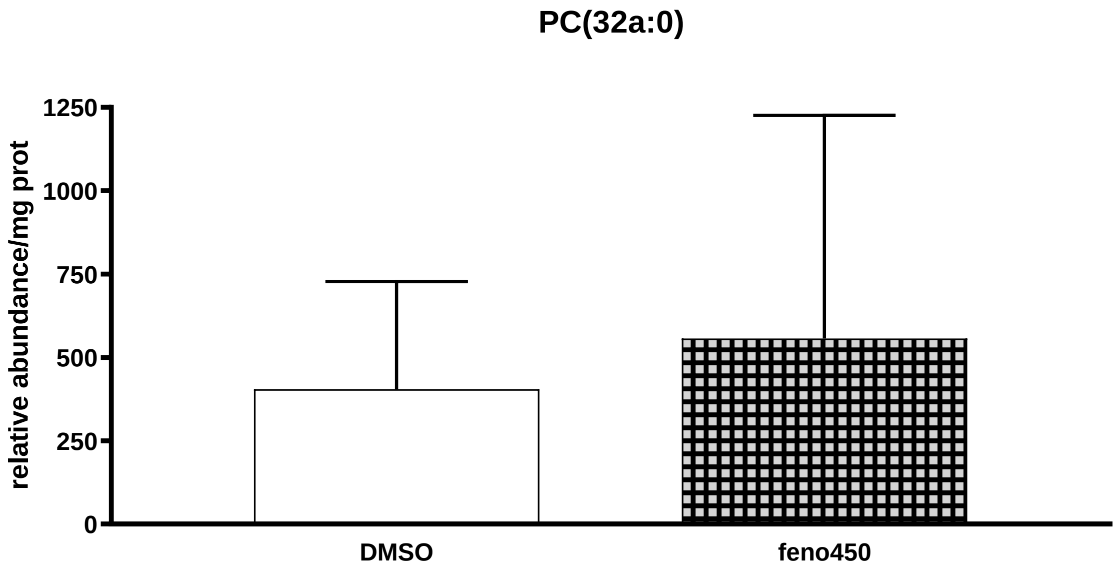
PC(32a:0)
relative abundance/mg prot
1250
1000
750
500
250
0
DMSO
feno450

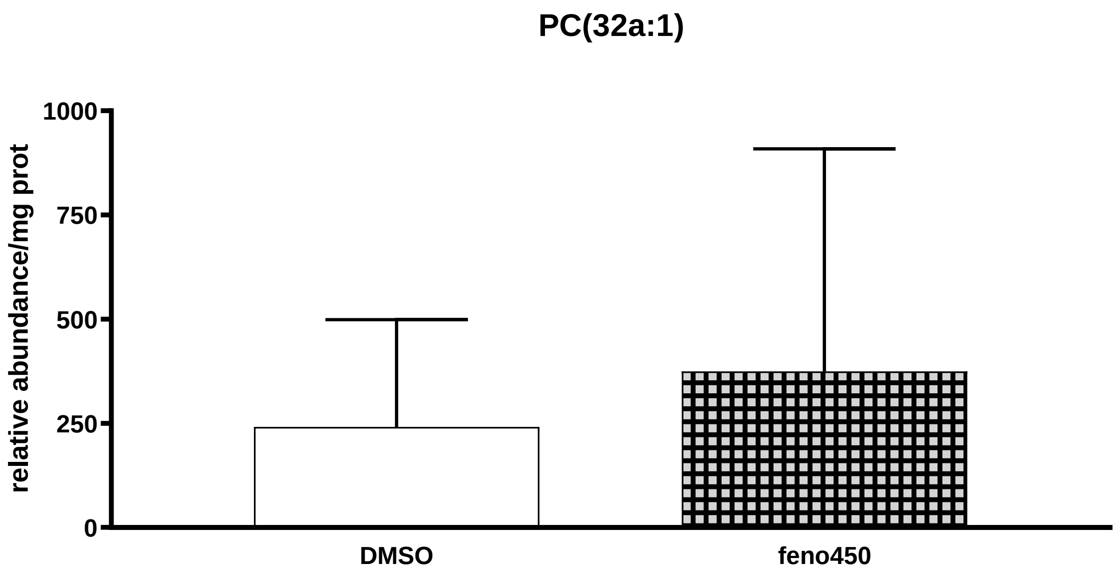
PC(32a:1)
relative abundance/mg prot
1000
750
500
250
0
DMSO
feno450

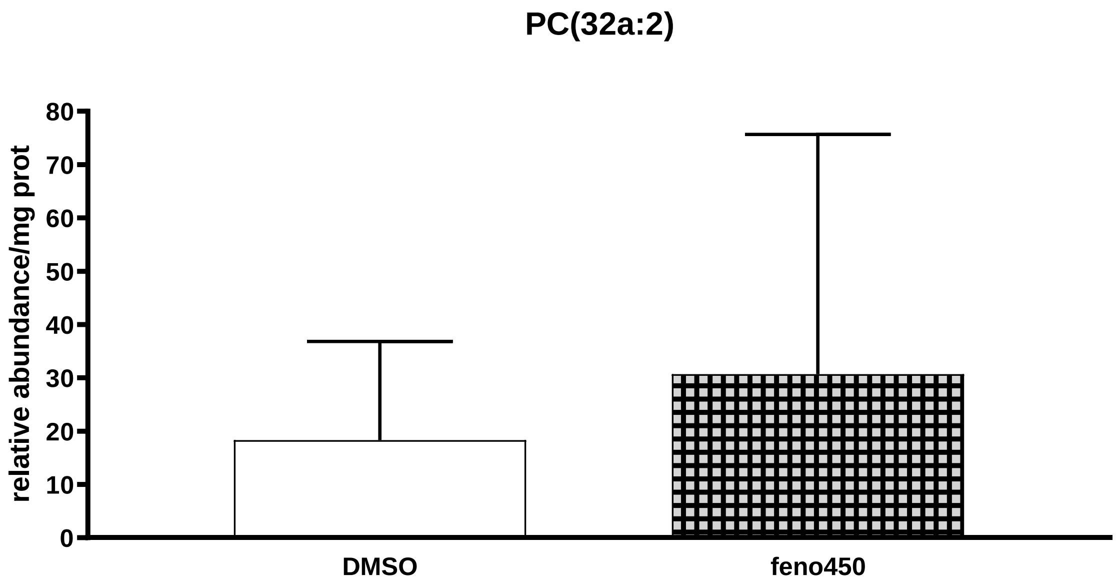
PC(32a:2)
relative abundance/mg prot
80
70
60
50
40
30
20
10
0
DMSO
feno450

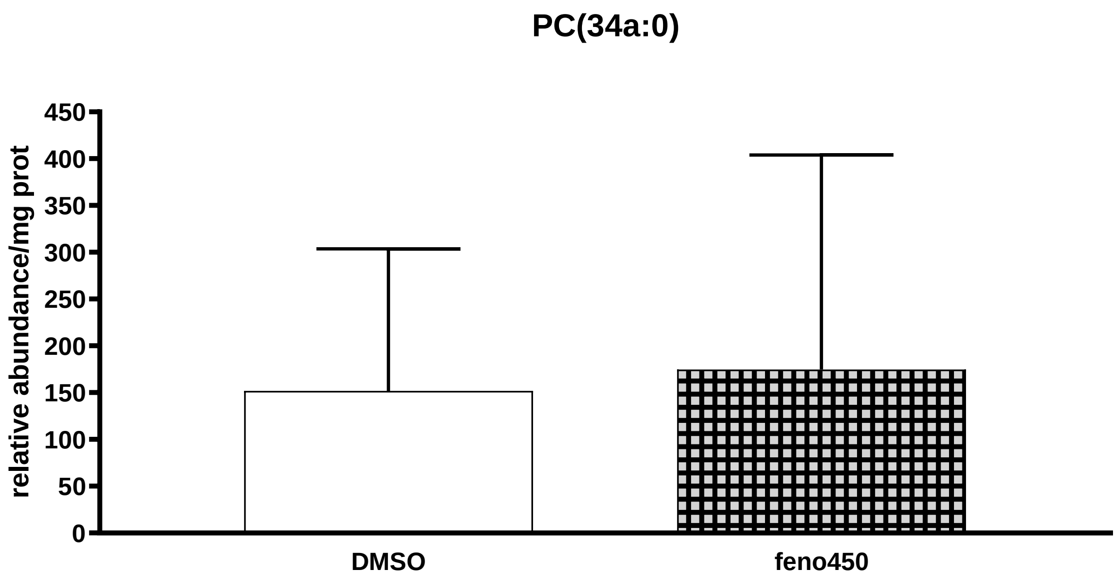
PC(34a:0)
relative abundance/mg prot
450
400
350
300
250
200
150
100
50
0
DMSO
feno450

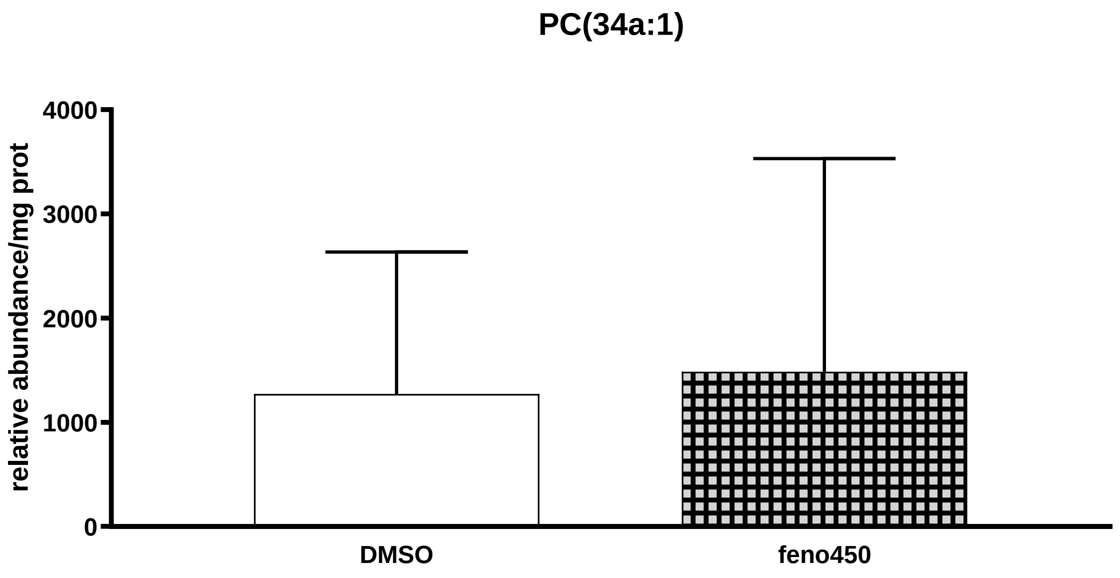
PC(34a:1)
relative abundance/mg prot
4000
3000
2000
1000
0
DMSO
feno450

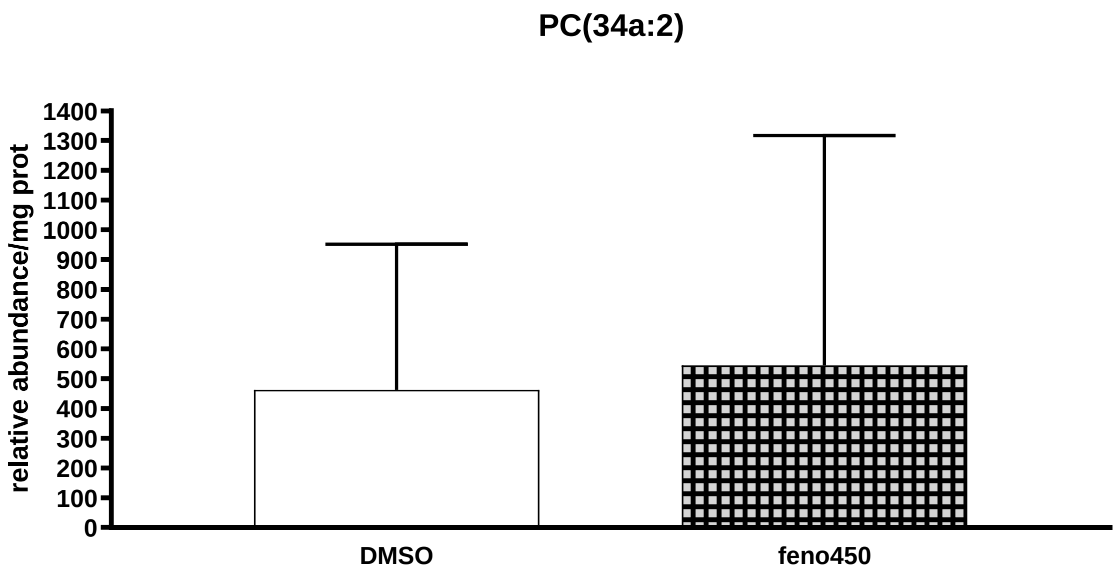
PC(34a:2)
relative abundance/mg prot
1400
1300
1200
1100
1000
900
800
700
600
500
400
300
200
100
0
DMSO
feno450

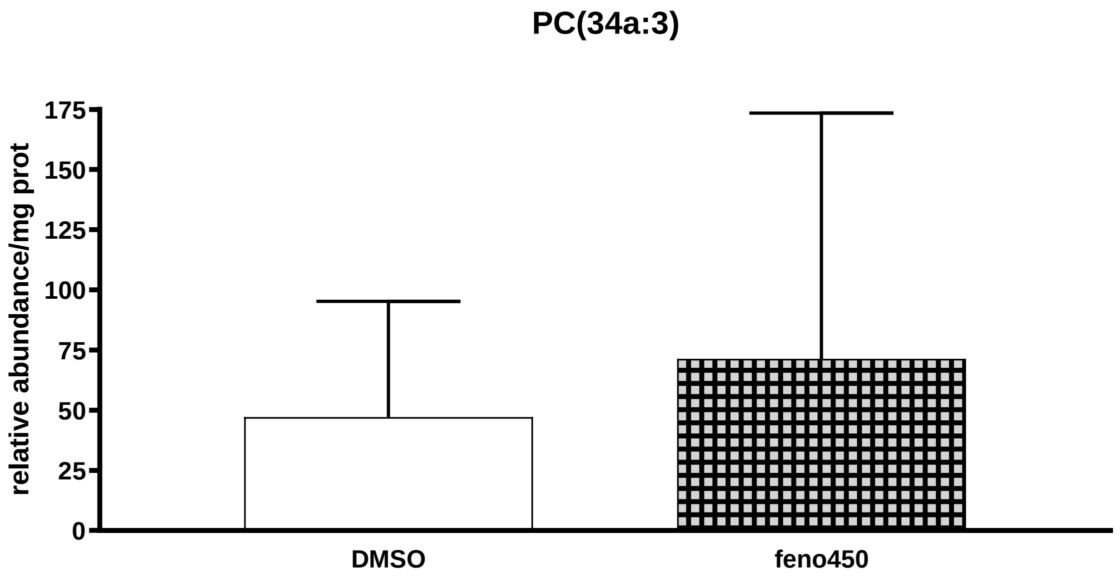
PC(34a:3)
relative abundance/mg prot
175
150
125
100
75
50
25
0
DMSO
feno450

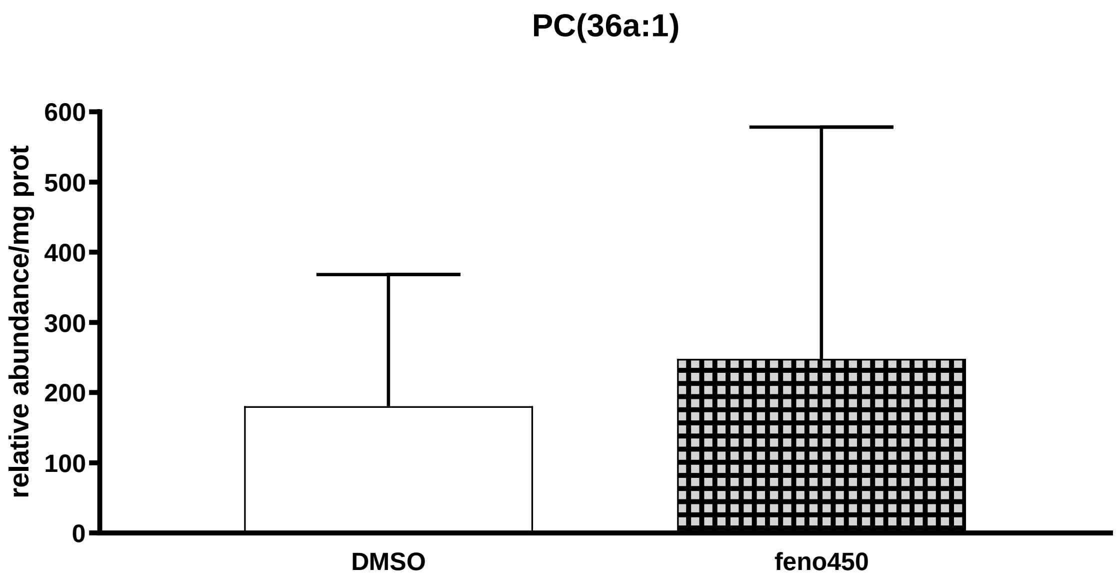
PC(36a:1)
relative abundance/mg prot
600
500
400
300
200
100
0
DMSO
feno450

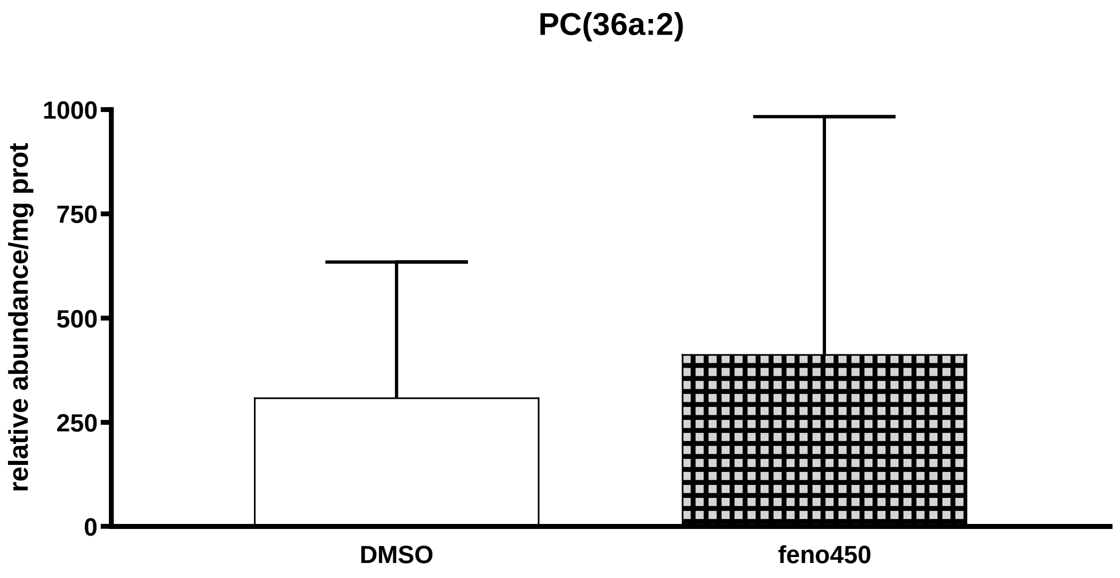
PC(36a:2)
relative abundance/mg prot
1000
750
500
250
0
DMSO
feno450

PC(36a:3)

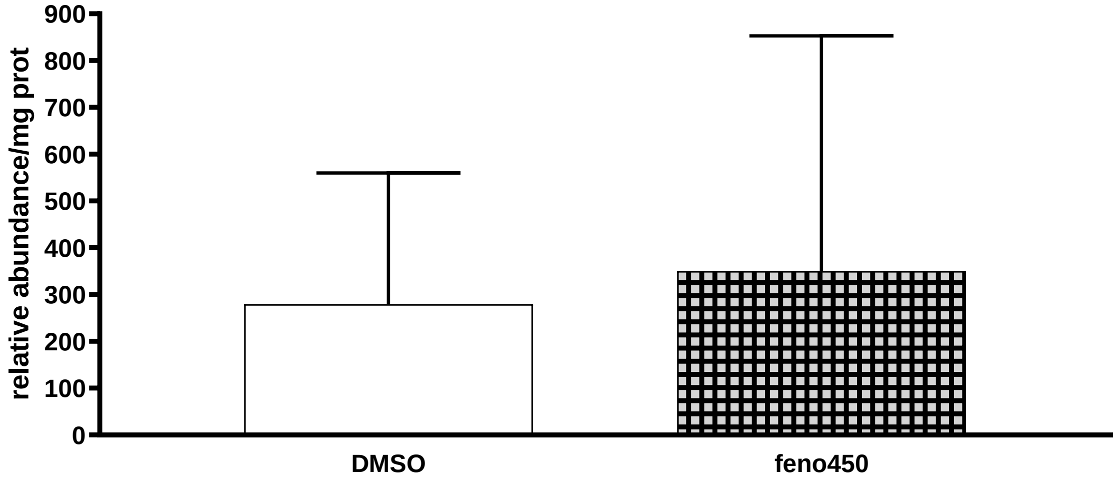

PC(36a:4)

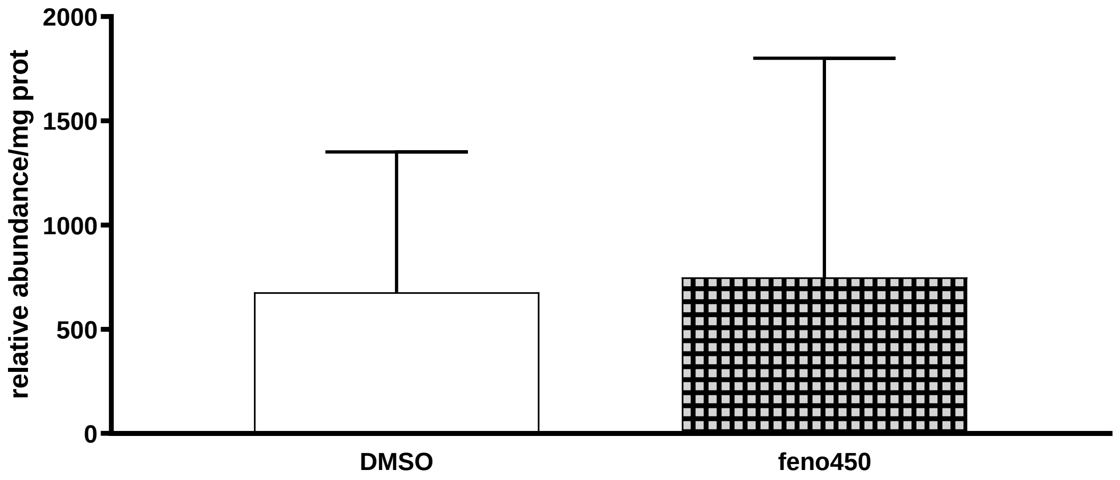

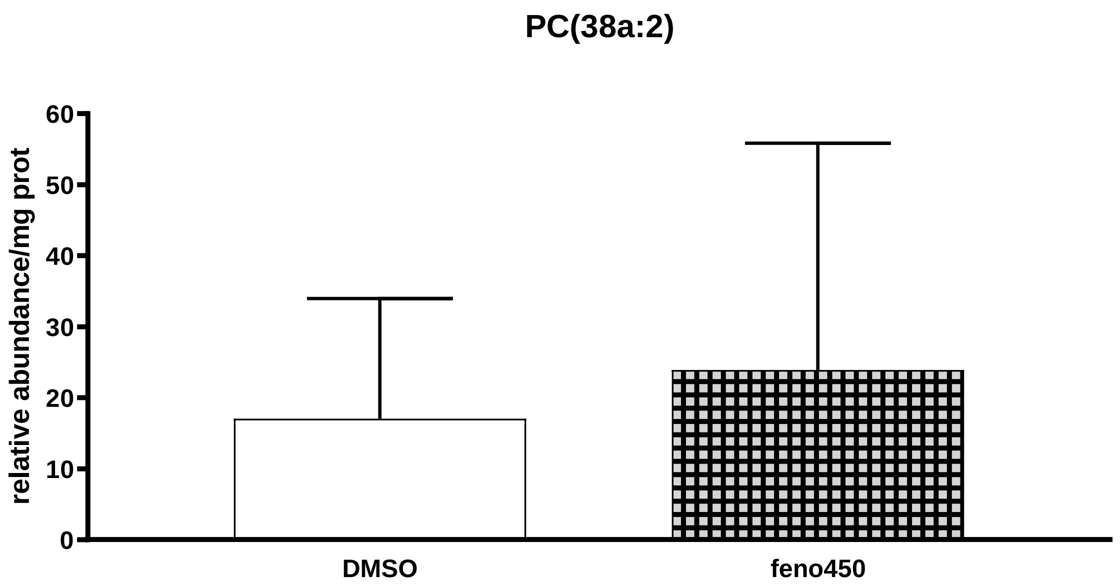
PC(38a:2)
relative abundance/mg prot
60
50
40
30
20
10
0
DMSO
feno450

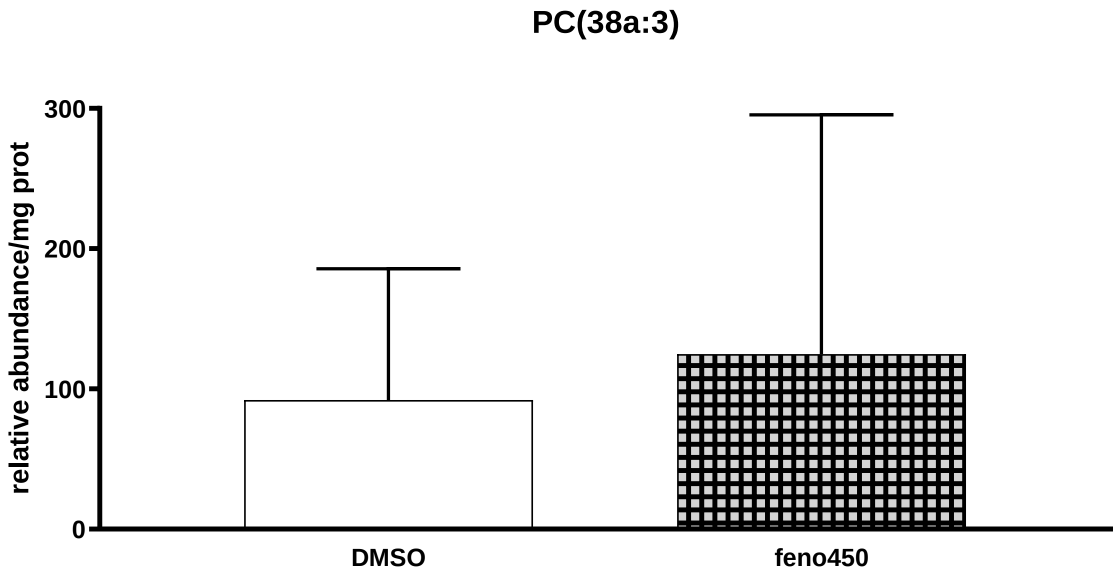
PC(38a:3)
relative abundance/mg prot
300
200
100
0
DMSO
feno450

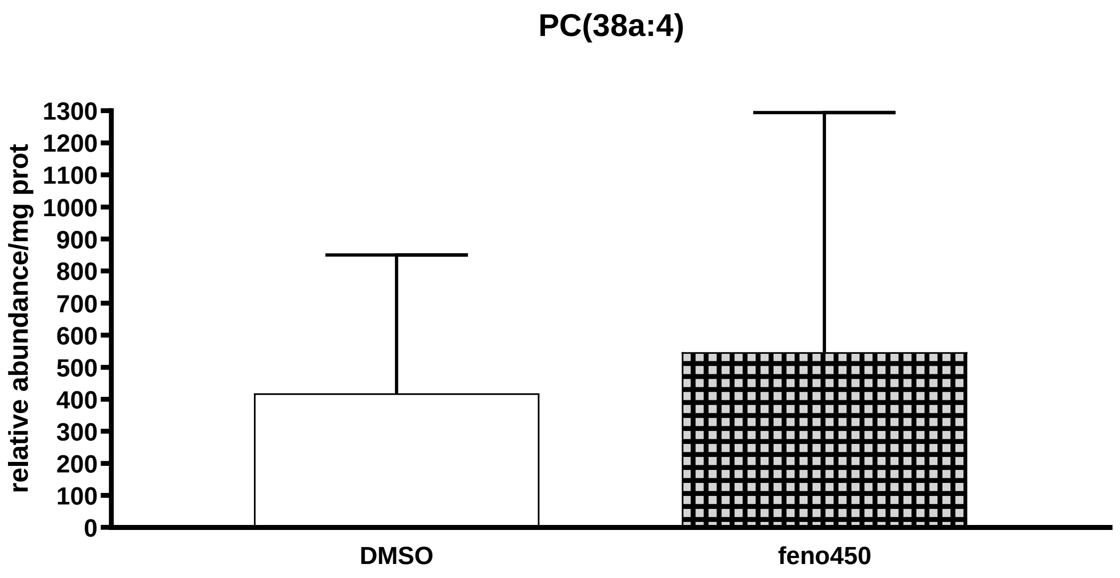
PC(38a:4)
relative abundance/mg prot
0
100
200
300
400
500
600
700
800
900
1000
1100
1200
1300
DMSO
feno450

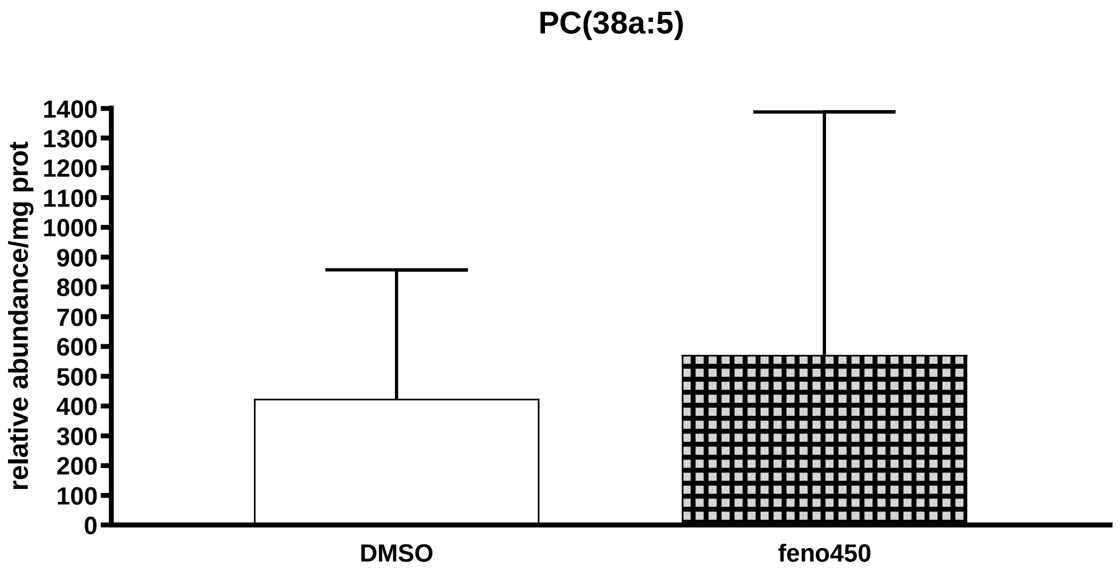
PC(38a:5)
relative abundance/mg prot
0
100
200
300
400
500
600
700
800
900
1000
1100
1200
1300
1400
DMSO
feno450

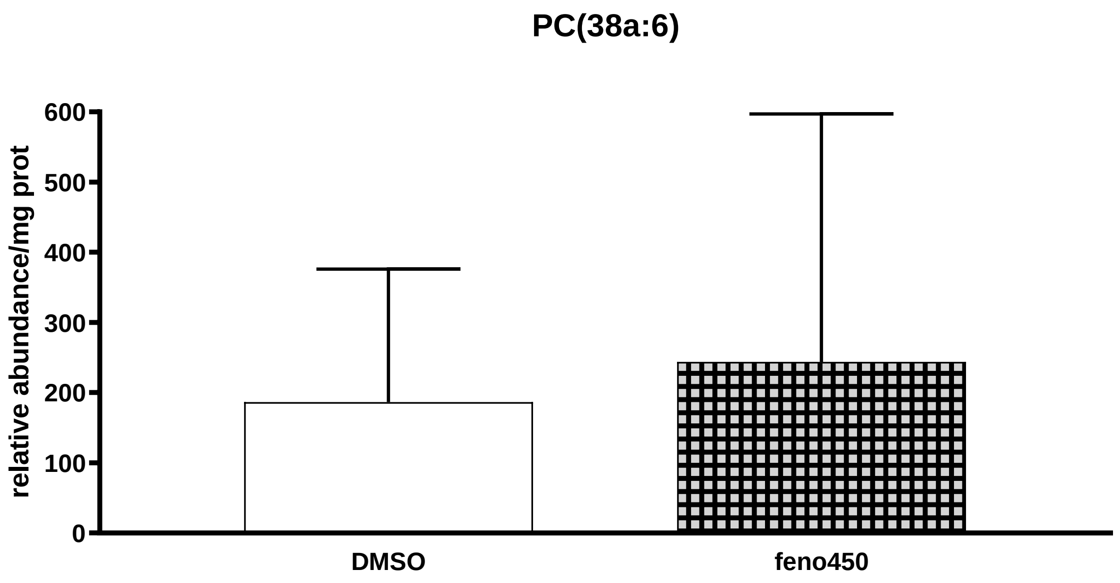
PC(38a:6)
relative abundance/mg prot
600
500
400
300
200
100
0
DMSO
feno450

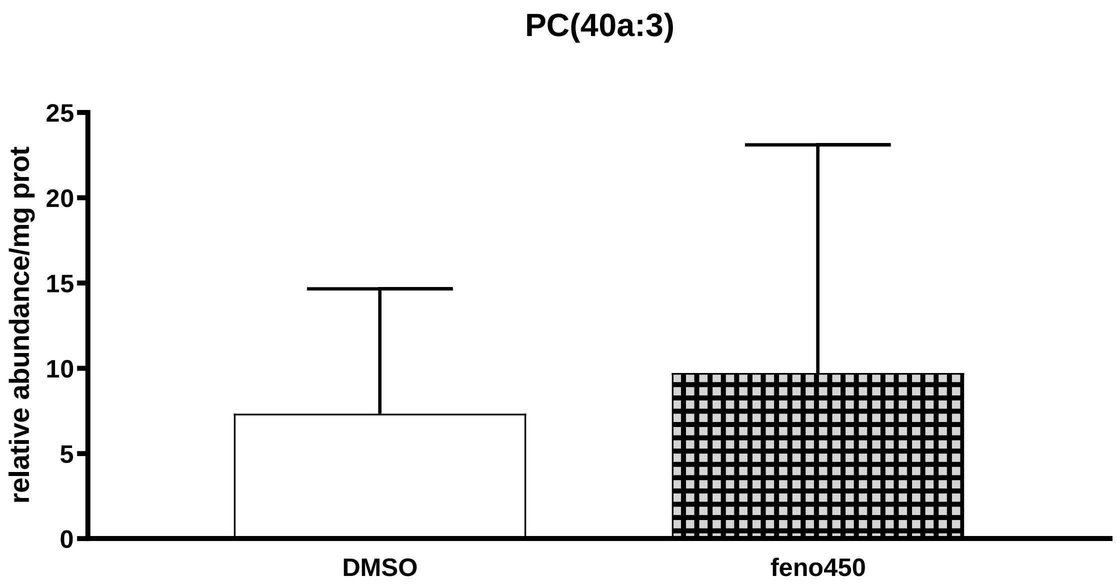
PC(40a:3)
relative abundance/mg prot
25
20
15
10
5
0
DMSO
feno450

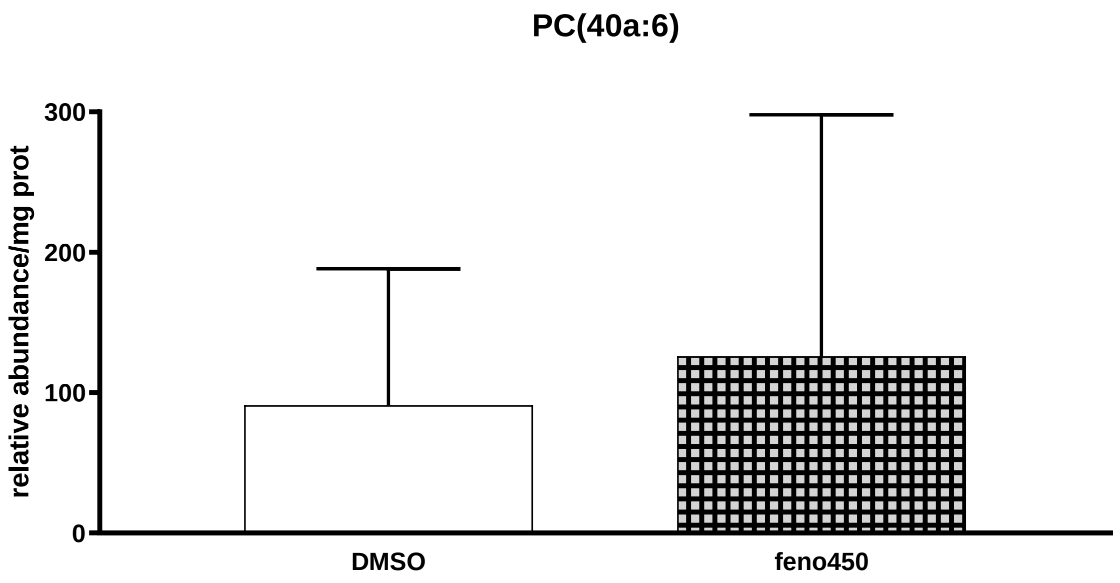
PC(40a:6)
300
200
100
0
relative abundance/mg prot
DMSO
feno450

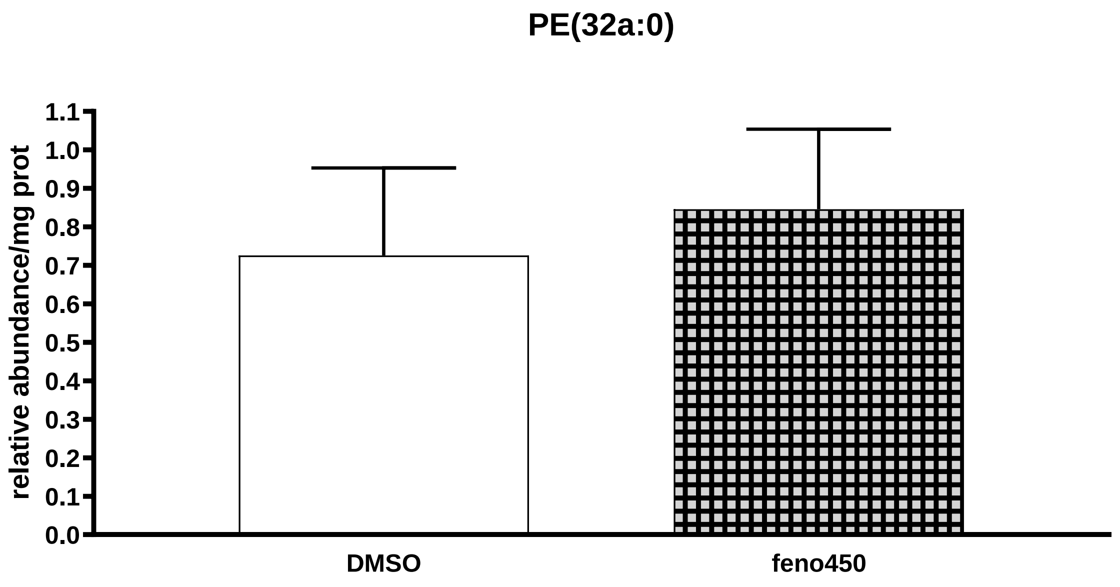
PE(32a:0)
1.1
1.0
0.9
0.8
0.7
0.6
0.5
0.4
0.3
0.2
0.1
0.0
relative abundance/mg prot
DMSO
feno450

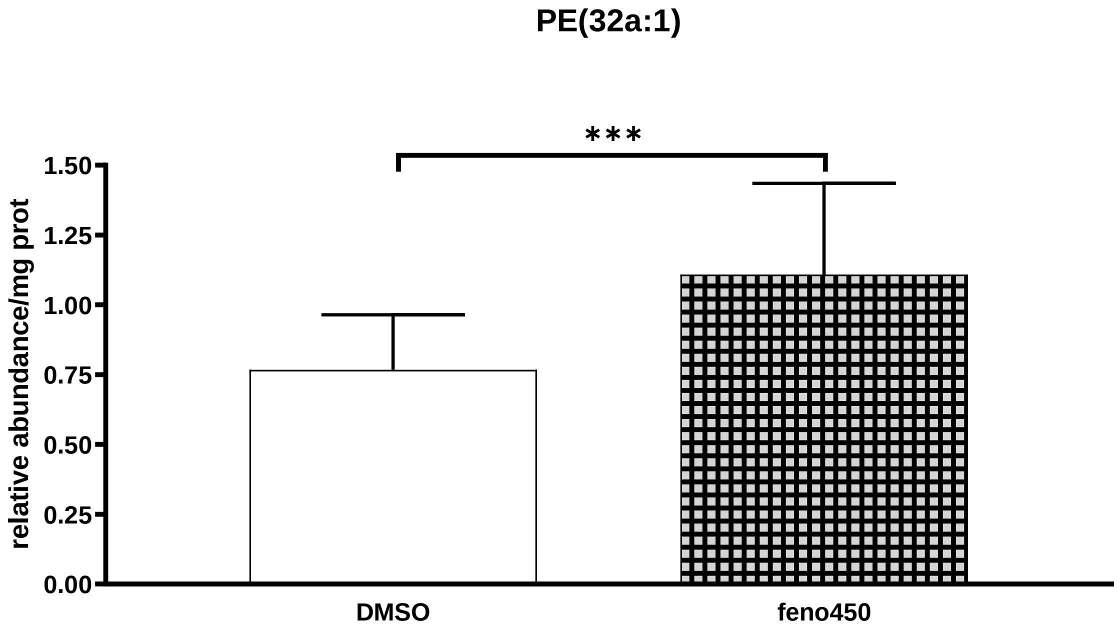
PE(32a:1)
***
1.50
1.25
1.00
0.75
0.50
0.25
0.00
relative abundance/mg prot
DMSO
feno450

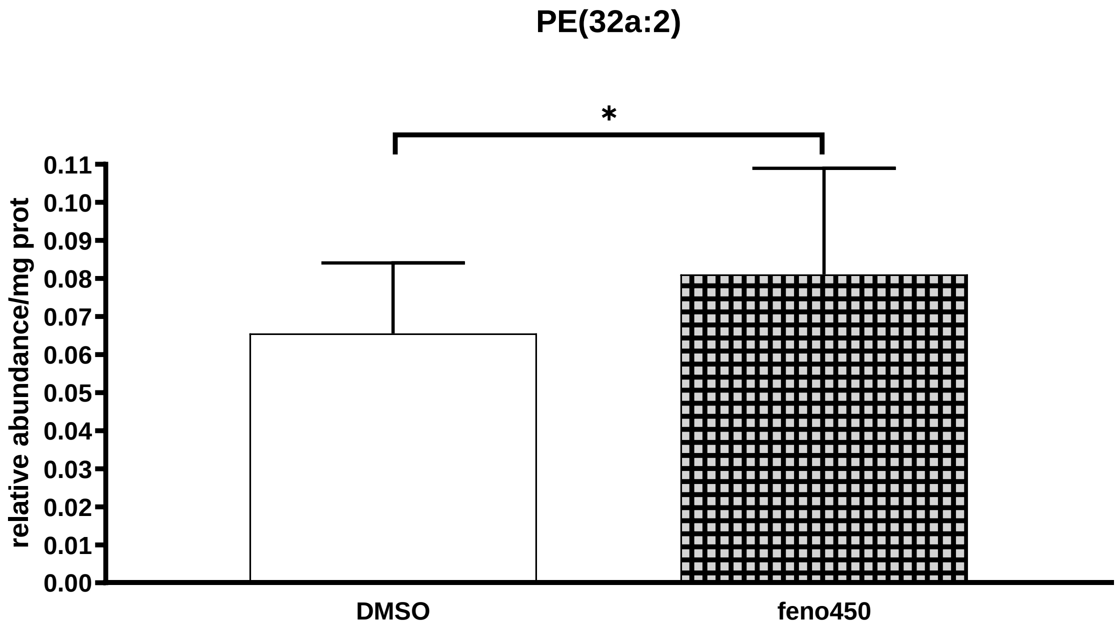
PE(32a:2)
*
0.11
0.10
0.09
0.08
0.07
0.06
0.05
0.04
0.03
0.02
0.01
0.00
relative abundance/mg prot
DMSO
feno450

PE(34a:0)

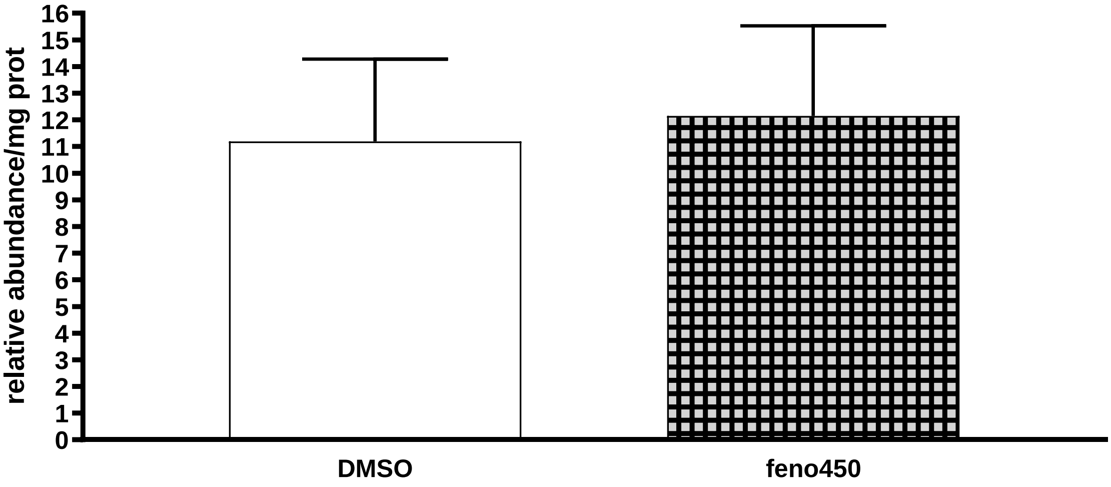

PE(34a:1)

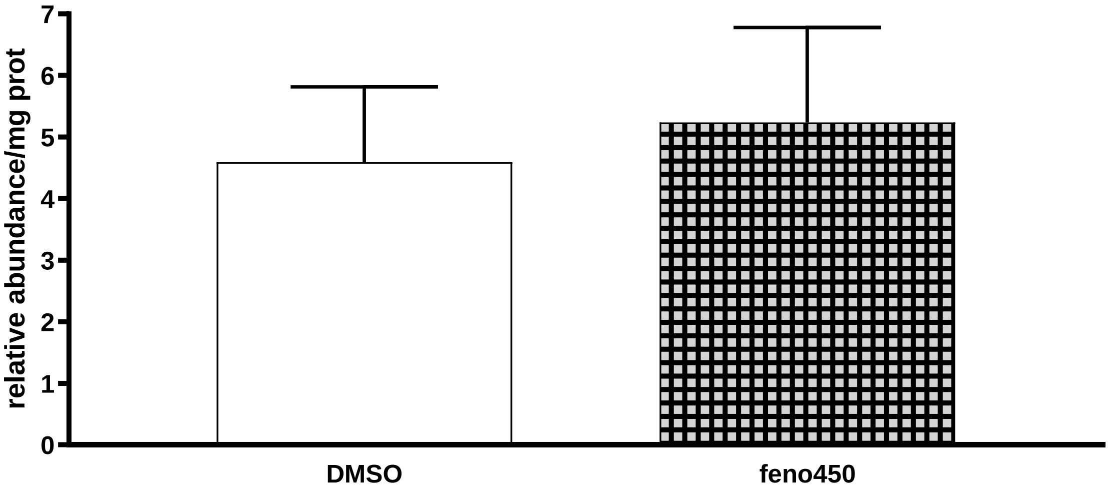

PE(36a:1)

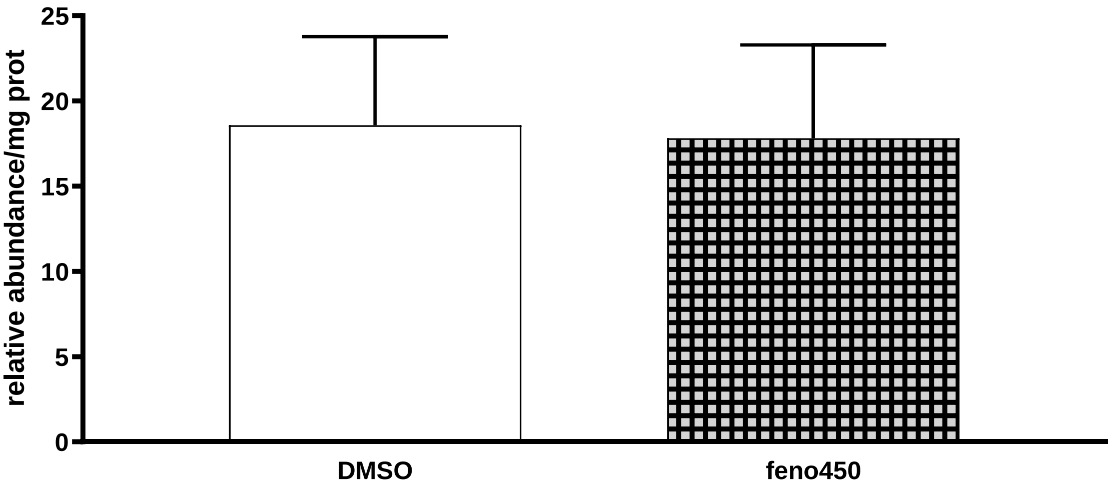

PE(36a:2)

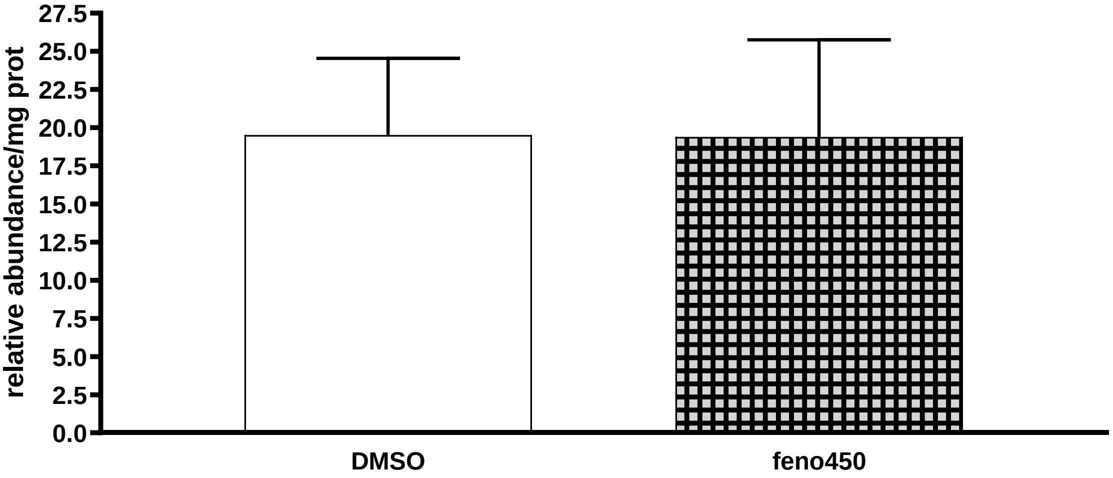

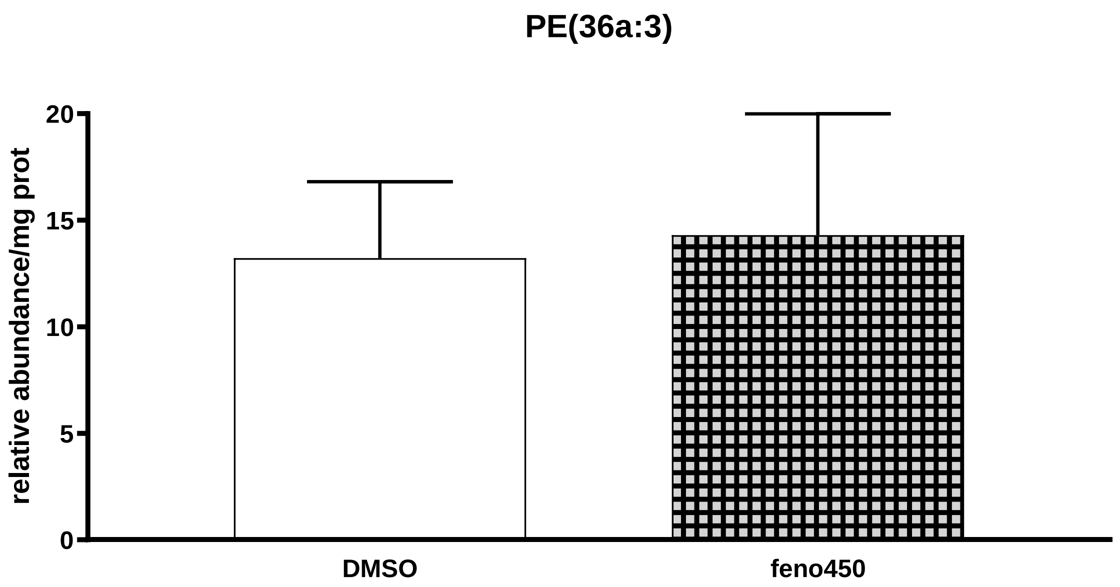
PE(36a:3)
relative abundance/mg prot
0
5
10
15
20
DMSO
feno450

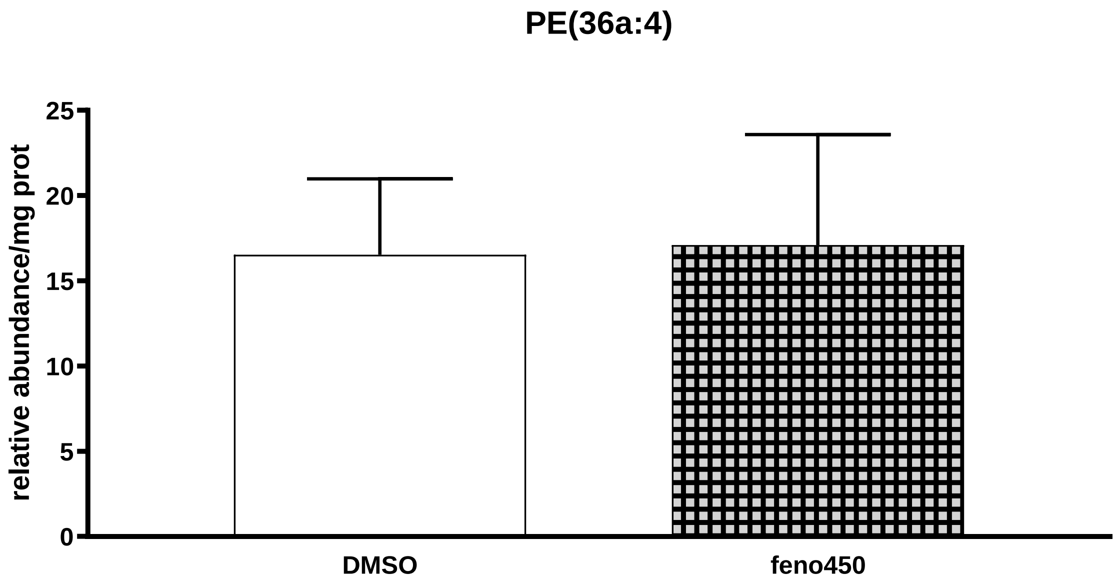
PE(36a:4)
relative abundance/mg prot
0
5
10
15
20
25
DMSO
feno450

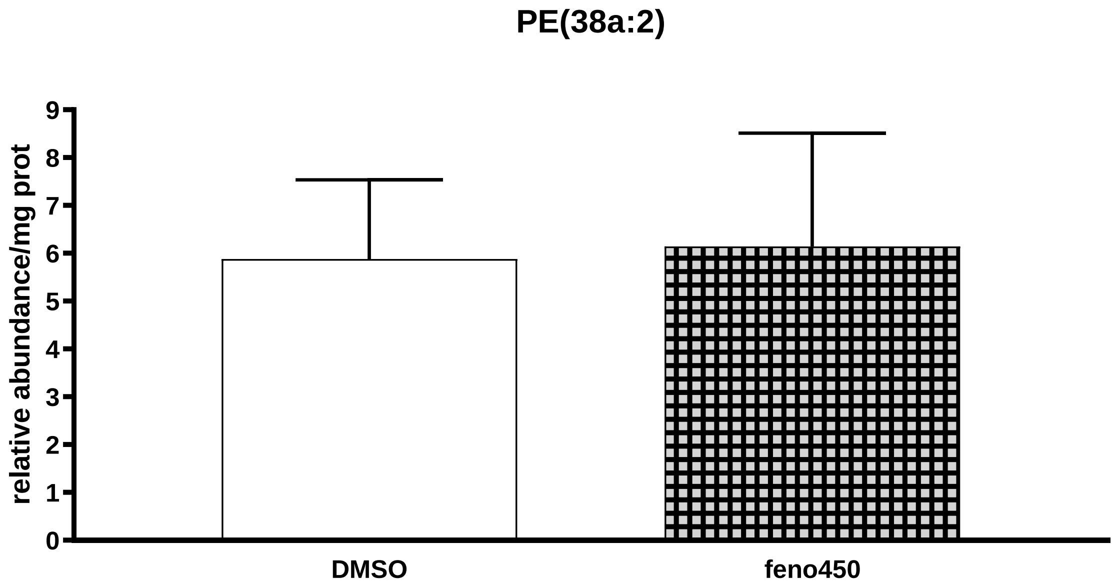
PE(38a:2)
relative abundance/mg prot
0
1
2
3
4
5
6
7
8
9
DMSO
feno450

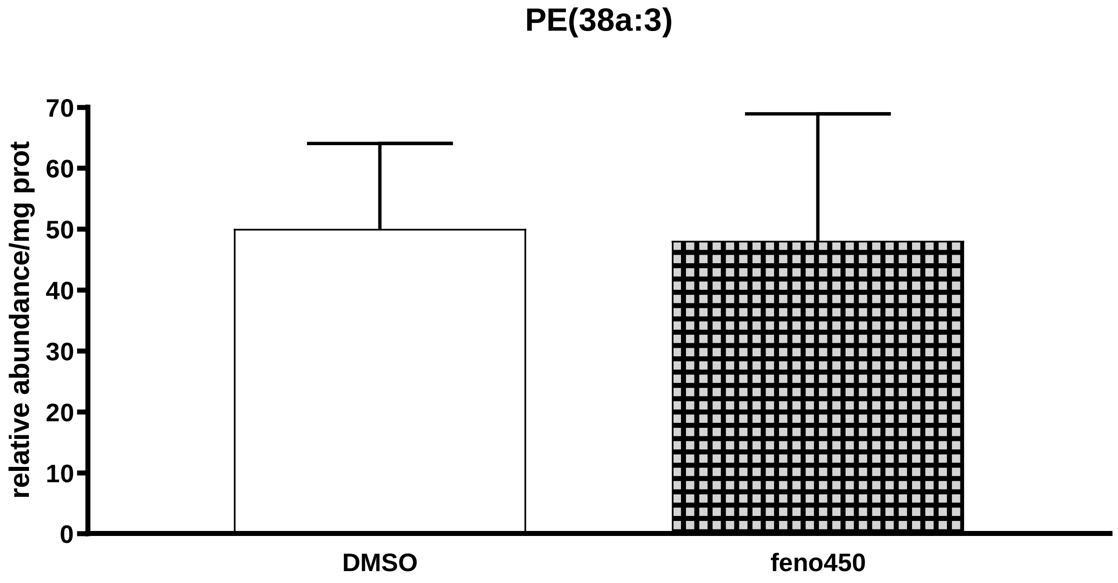
PE(38a:3)
relative abundance/mg prot
0
10
20
30
40
50
60
70
DMSO
feno450

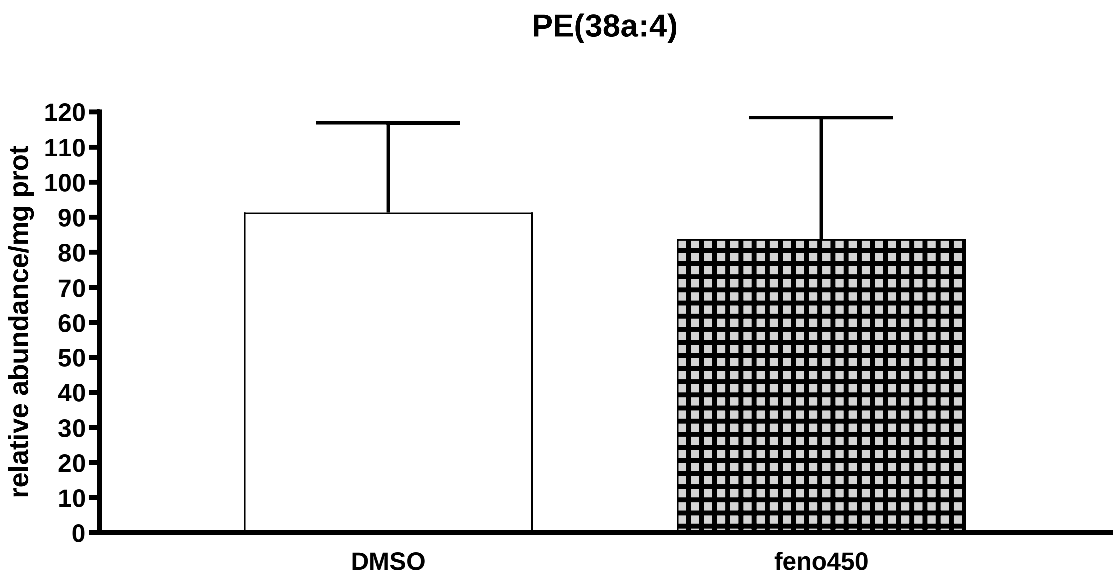
PE(38a:4)
relative abundance/mg prot
0
10
20
30
40
50
60
70
80
90
100
110
120
DMSO
feno450

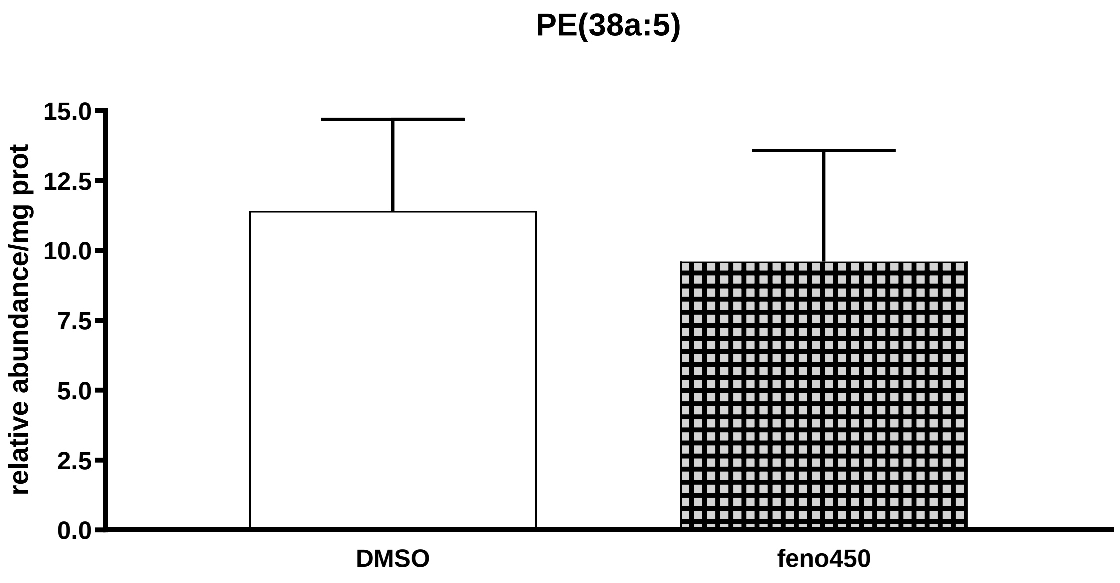
PE(38a:5)
relative abundance/mg prot
0.0
2.5
5.0
7.5
10.0
12.5
15.0
DMSO
feno450

PE(38a:6)

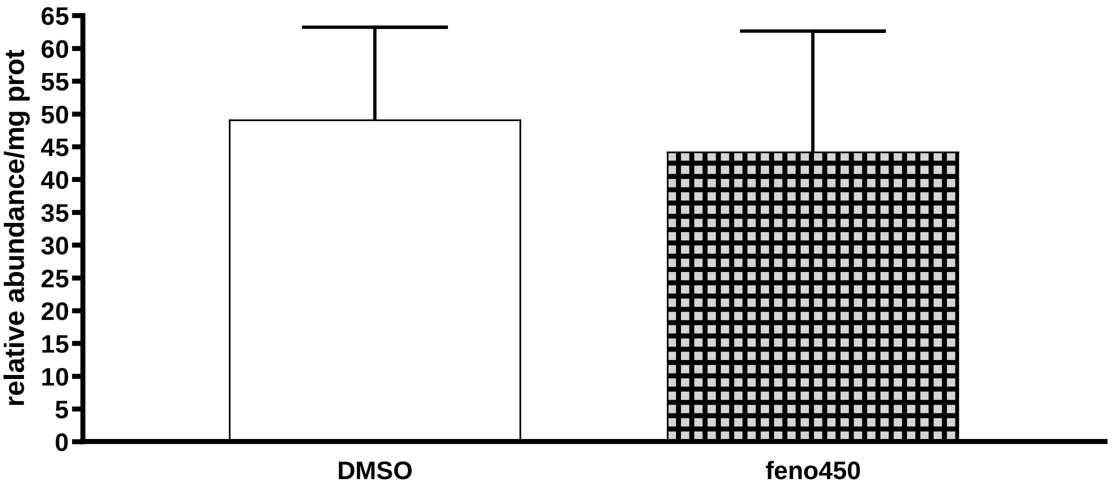

PE(40a:3)

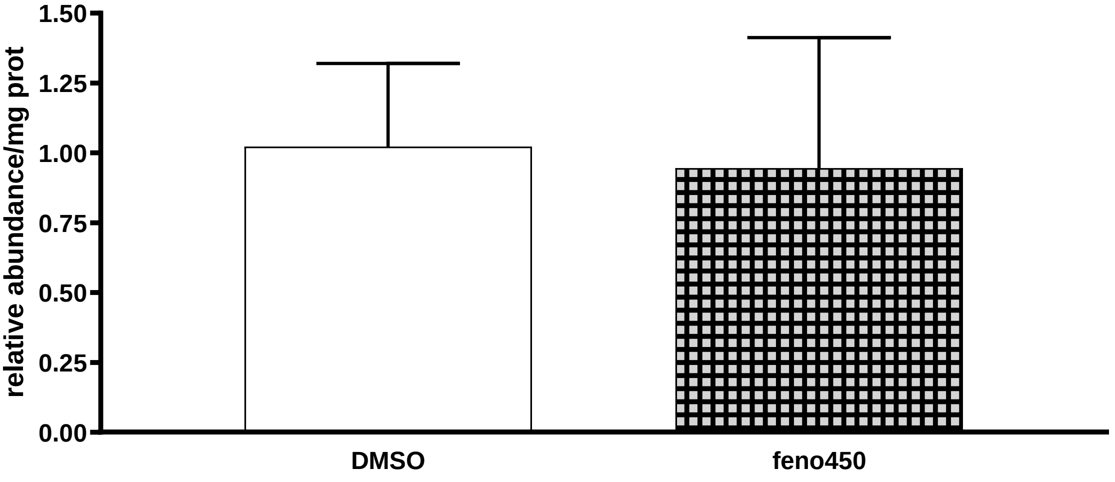

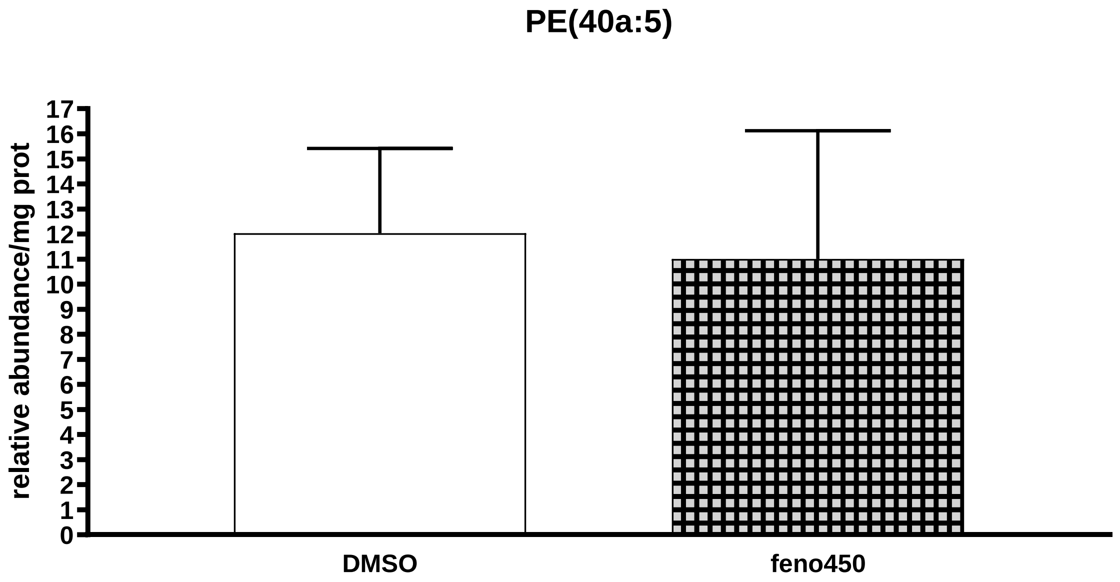
PE(40a:5)
relative abundance/mg prot
17
16
15
14
13
12
11
10
9
8
7
6
5
4
3
2
1
0
DMSO
feno450

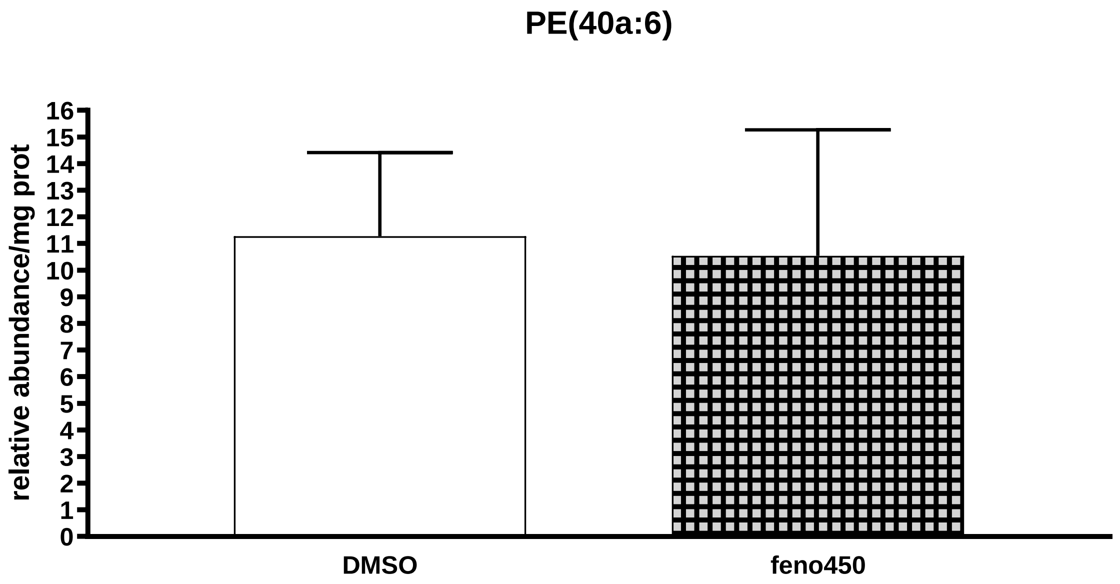
PE(40a:6)
relative abundance/mg prot
16
15
14
13
12
11
10
9
8
7
6
5
4
3
2
1
0
DMSO
feno450

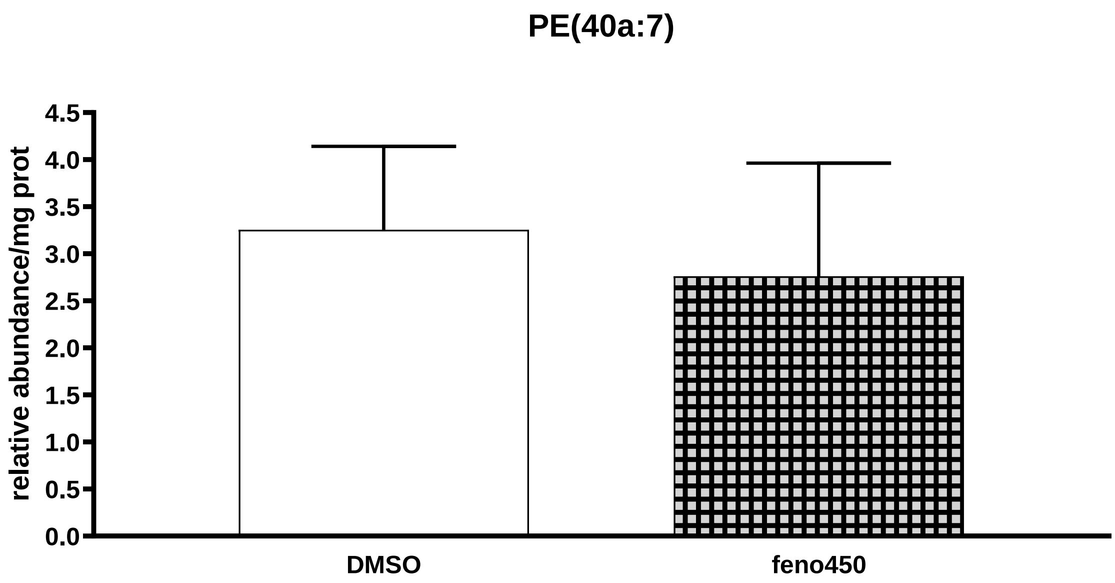
PE(40a:7)
relative abundance/mg prot
4.5
4.0
3.5
3.0
2.5
2.0
1.5
1.0
0.5
0.0
DMSO
feno450

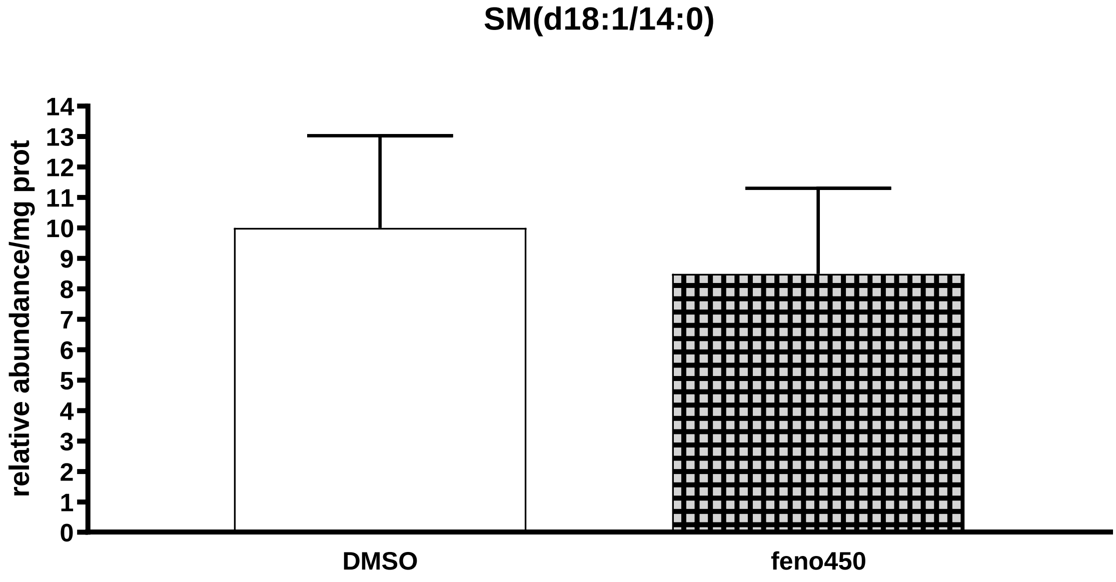
SM(d18:1/14:0)
relative abundance/mg prot
14
13
12
11
10
9
8
7
6
5
4
3
2
1
0
DMSO
feno450

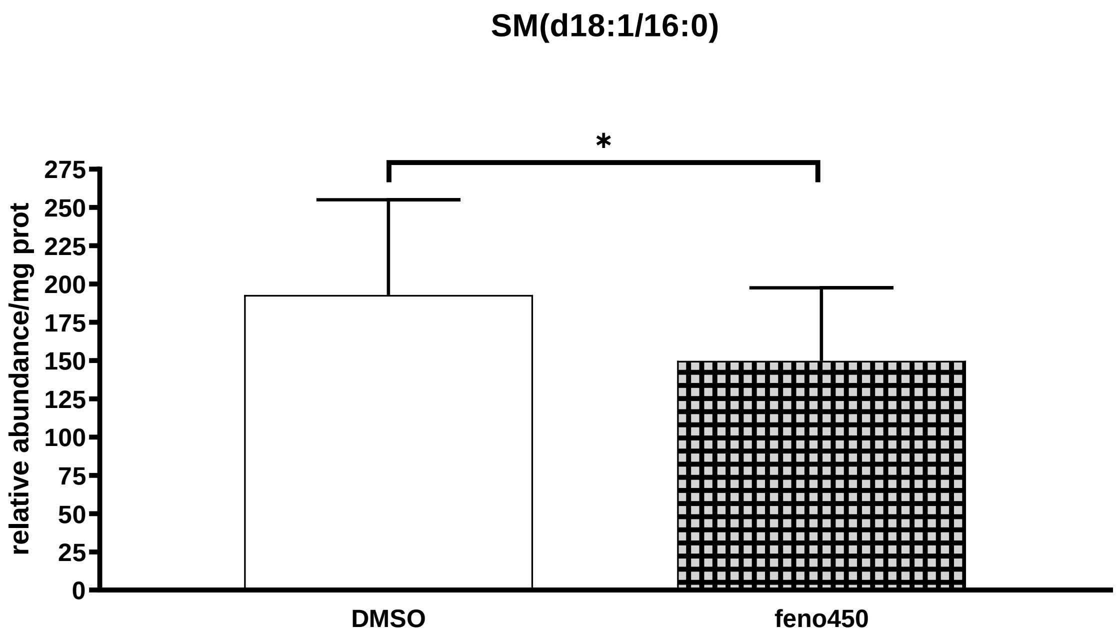
SM(d18:1/16:0)
*
relative abundance/mg prot
275
250
225
200
175
150
125
100
75
50
25
0
DMSO
feno450

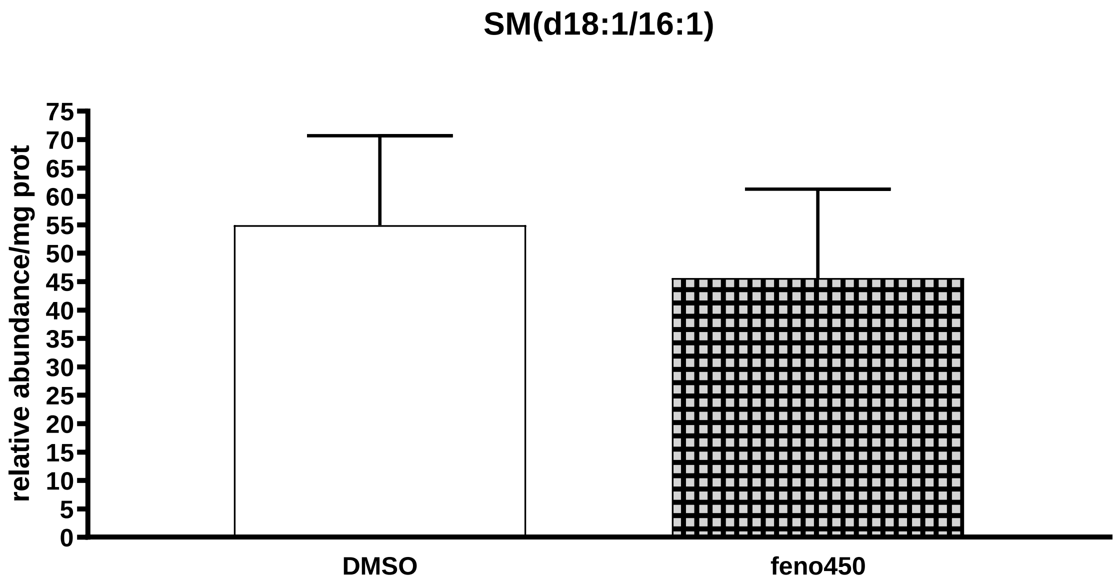
SM(d18:1/16:1)
relative abundance/mg prot
75
70
65
60
55
50
45
40
35
30
25
20
15
10
5
0
DMSO
feno450

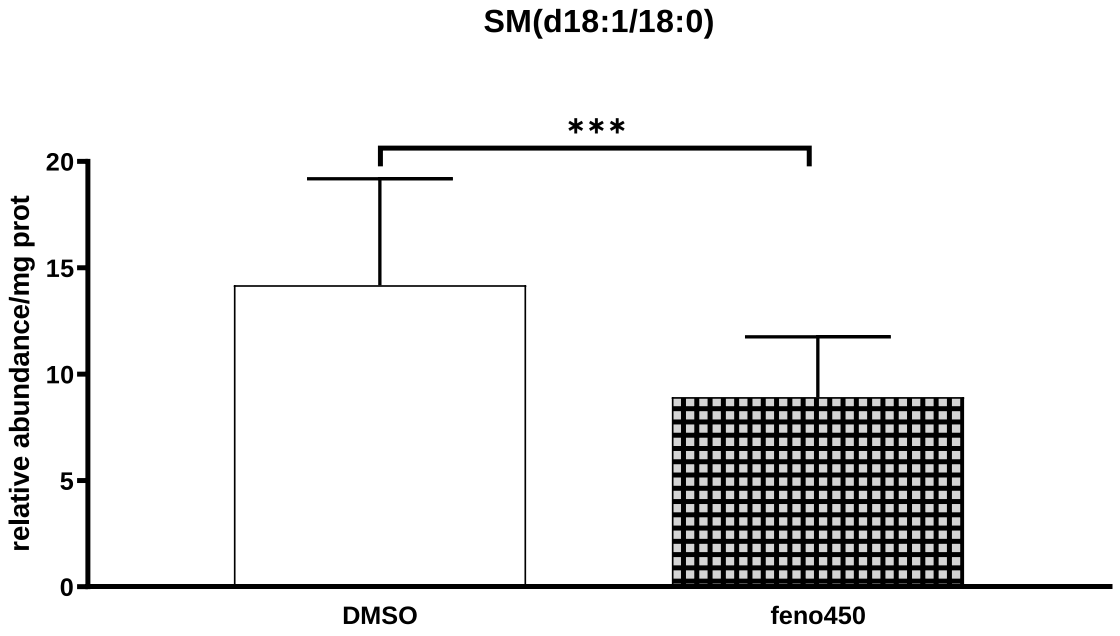
SM(d18:1/18:0)
***
relative abundance/mg prot
20
15
10
5
0
DMSO
feno450

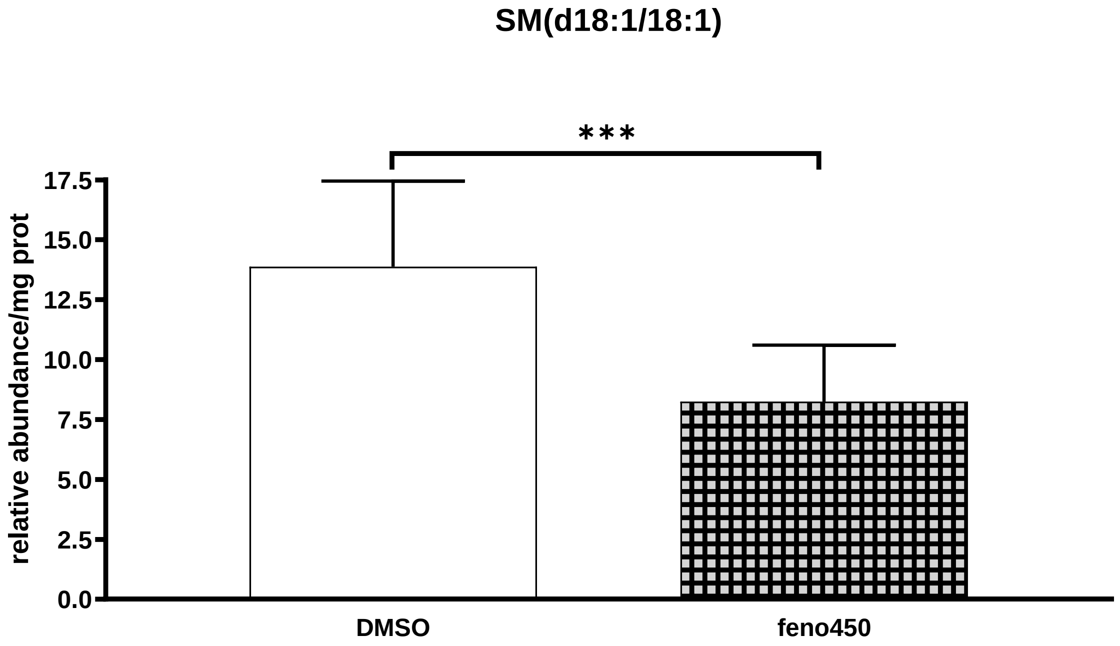
SM(d18:1/18:1)
***
relative abundance/mg prot
17.5
15.0
12.5
10.0
7.5
5.0
2.5
0.0
DMSO
feno450

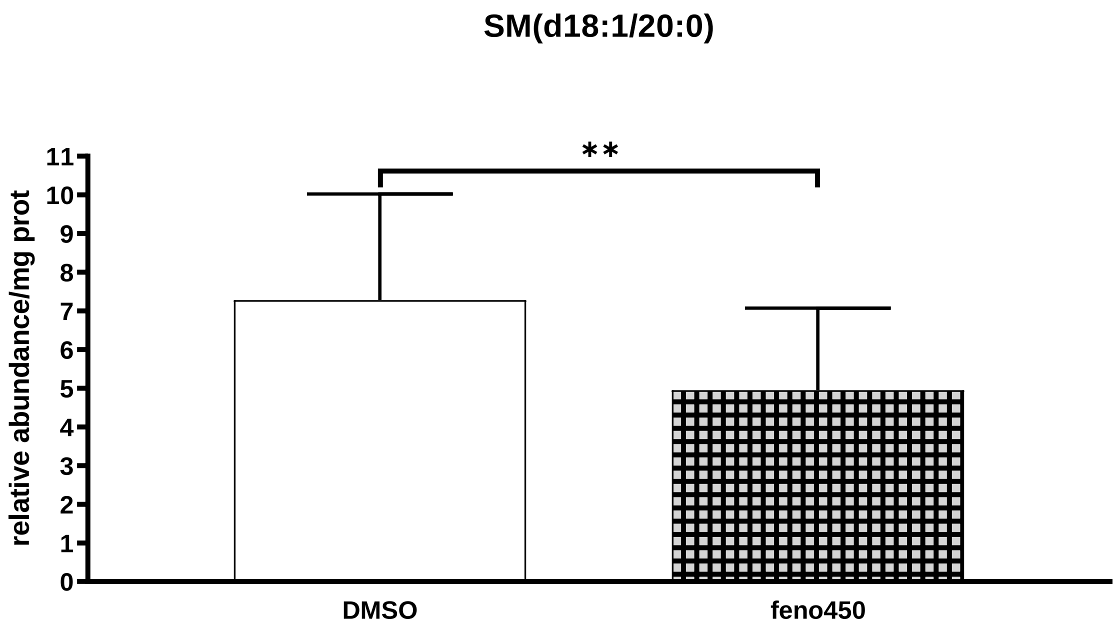
SM(d18:1/20:0)
**
relative abundance/mg prot
0
1
2
3
4
5
6
7
8
9
10
11
DMSO
feno450

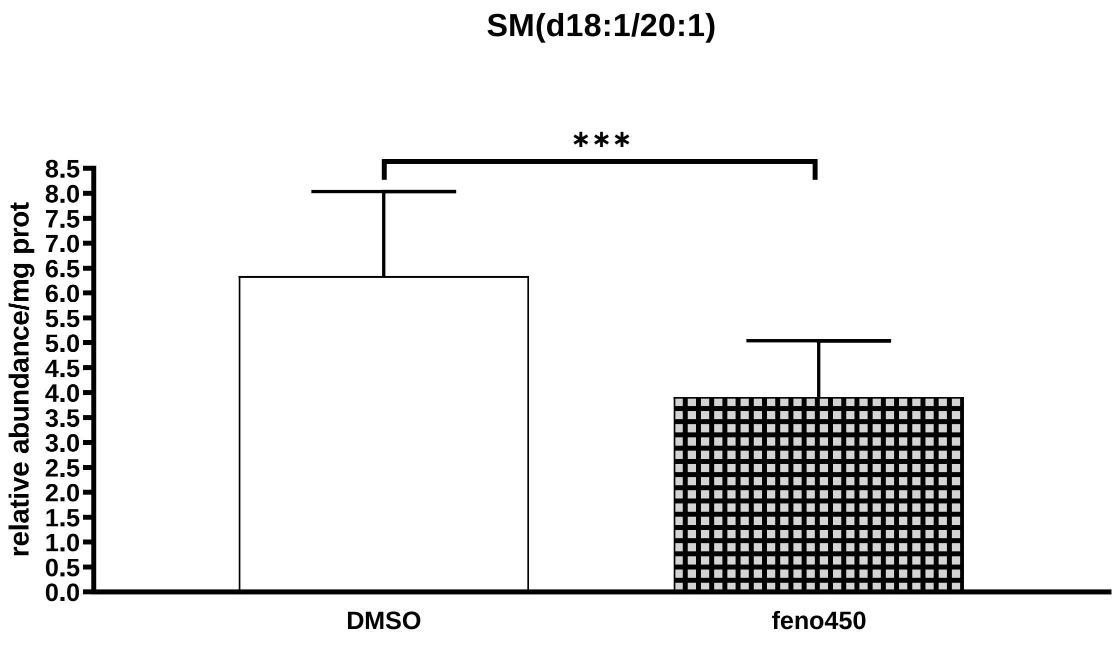
SM(d18:1/20:1)
***
relative abundance/mg prot
0.0
0.5
1.0
1.5
2.0
2.5
3.0
3.5
4.0
4.5
5.0
5.5
6.0
6.5
7.0
7.5
8.0
8.5
DMSO
feno450

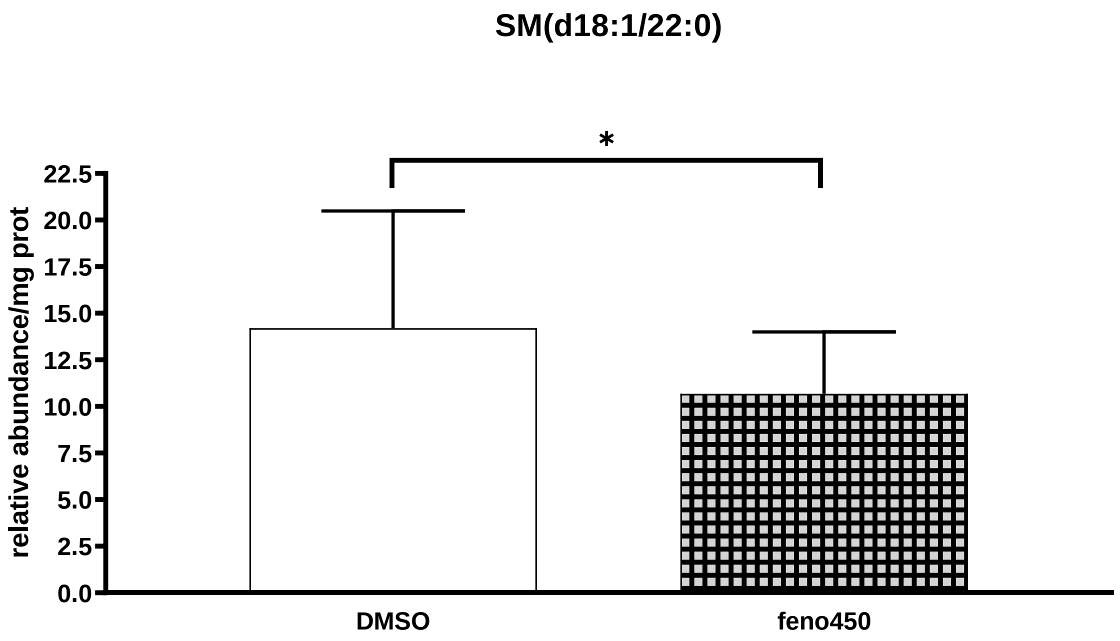
SM(d18:1/22:0)
*
relative abundance/mg prot
22.5
20.0
17.5
15.0
12.5
10.0
7.5
5.0
2.5
0.0
DMSO
feno450

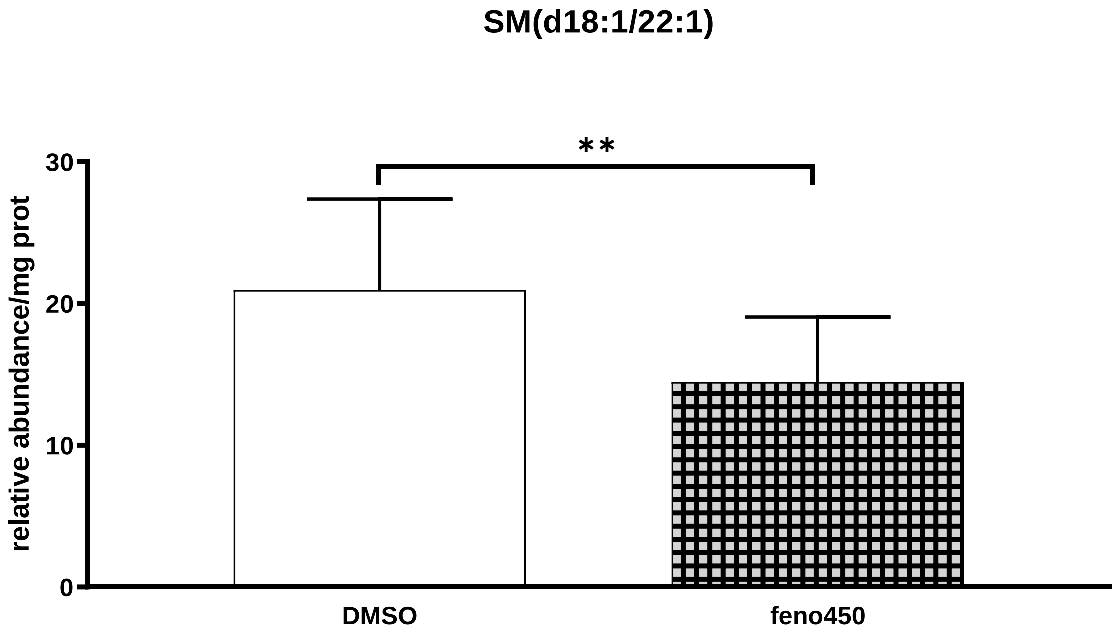
SM(d18:1/22:1)
**
relative abundance/mg prot
30
20
10
0
DMSO
feno450

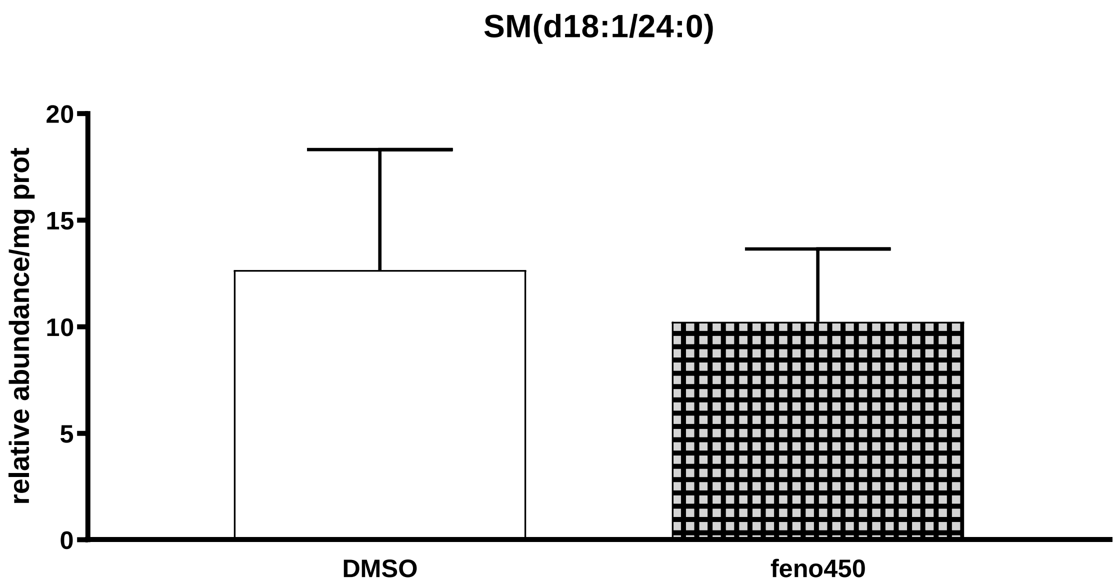

SM(d18:1/24:0)
relative abundance/mg prot
20
15
10
5
0
DMSO
feno450

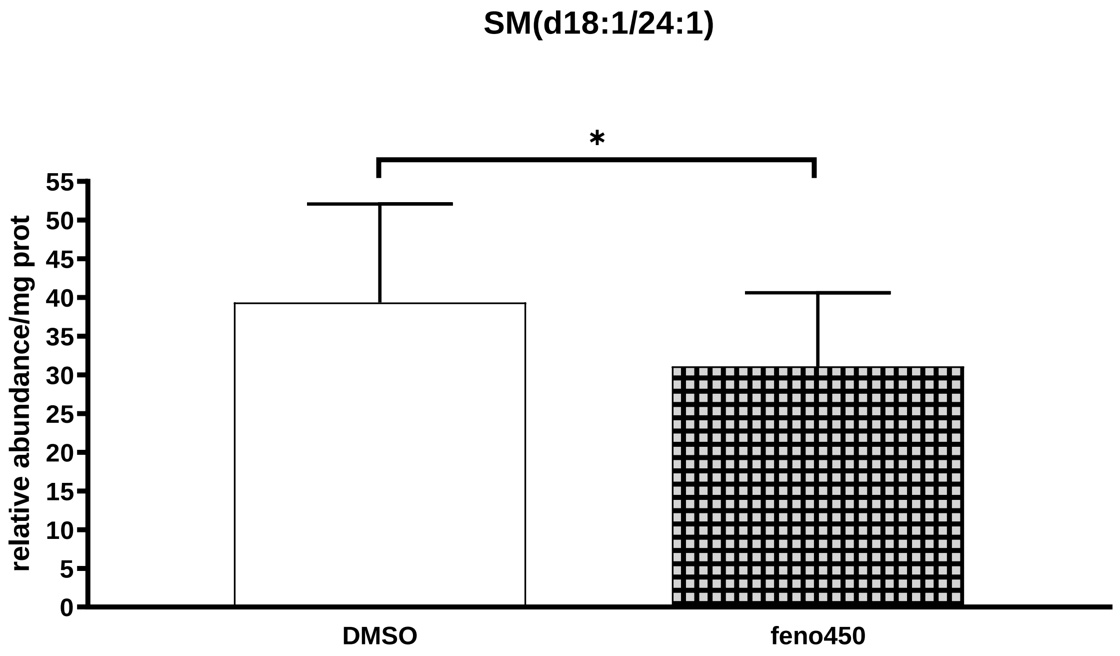

SM(d18:1/24:1)
*
relative abundance/mg prot
55
50
45
40
35
30
25
20
15
10
5
0
DMSO
feno450

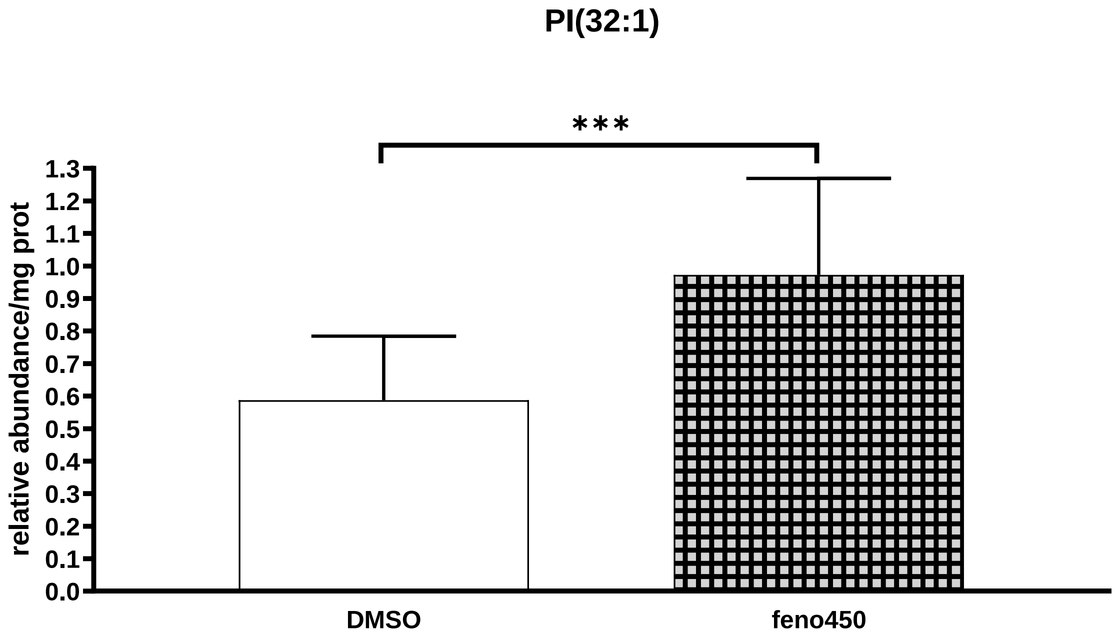
PI(32:1)
***
relative abundance/mg prot
1.3
1.2
1.1
1.0
0.9
0.8
0.7
0.6
0.5
0.4
0.3
0.2
0.1
0.0
DMSO
feno450

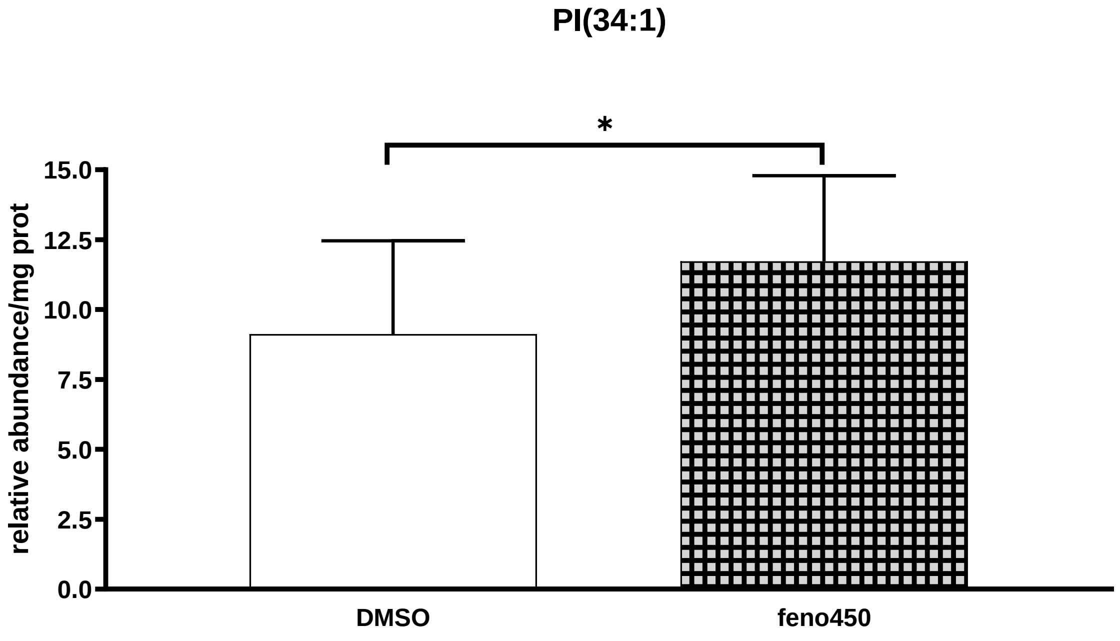
PI(34:1)
*
relative abundance/mg prot
15.0
12.5
10.0
7.5
5.0
2.5
0.0
DMSO
feno450

PI(34:2)

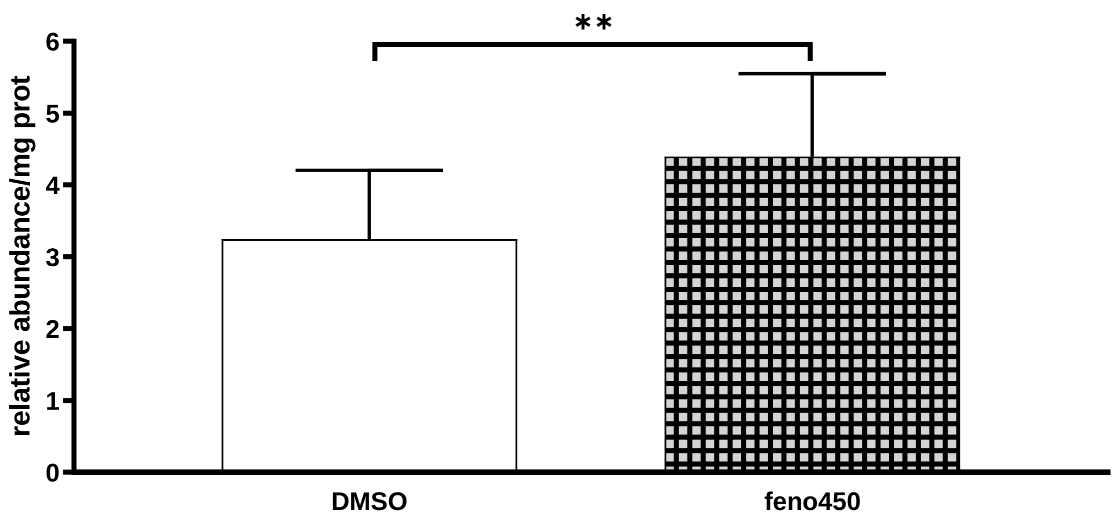
**
6
5
4
3
2
1
0
relative abundance/mg prot
DMSO
feno450

PI(36:0)

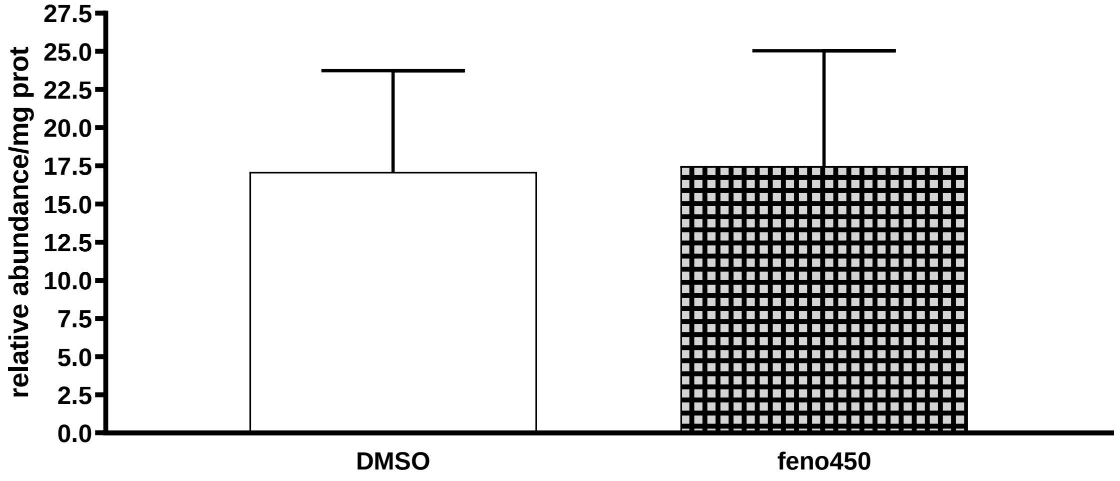
27.5
25.0
22.5
20.0
17.5
15.0
12.5
10.0
7.5
5.0
2.5
0.0
relative abundance/mg prot
DMSO
feno450

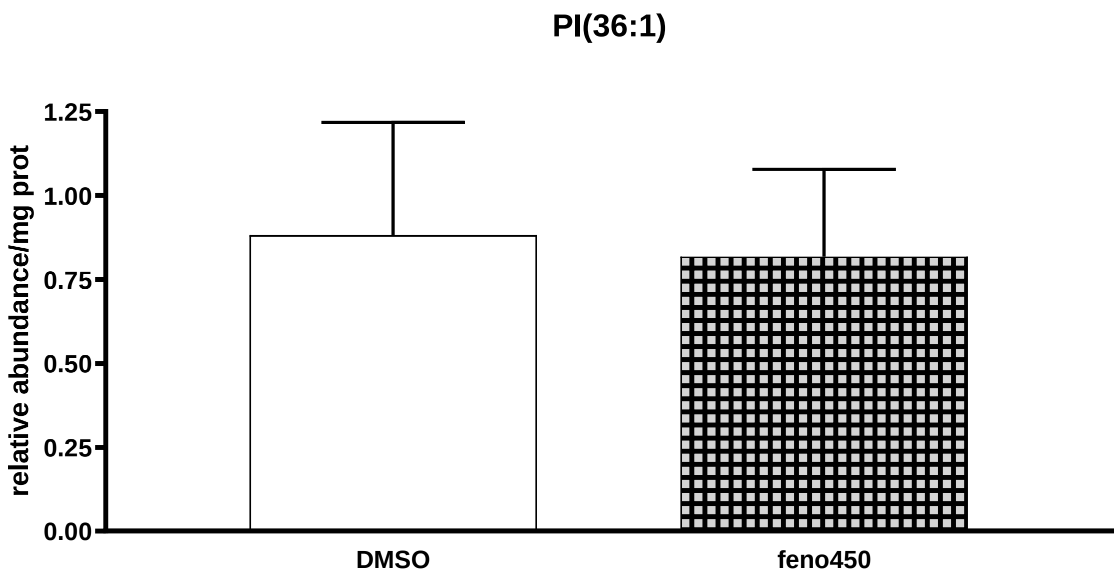
PI(36:1)
relative abundance/mg prot
1.25
1.00
0.75
0.50
0.25
0.00
DMSO
feno450

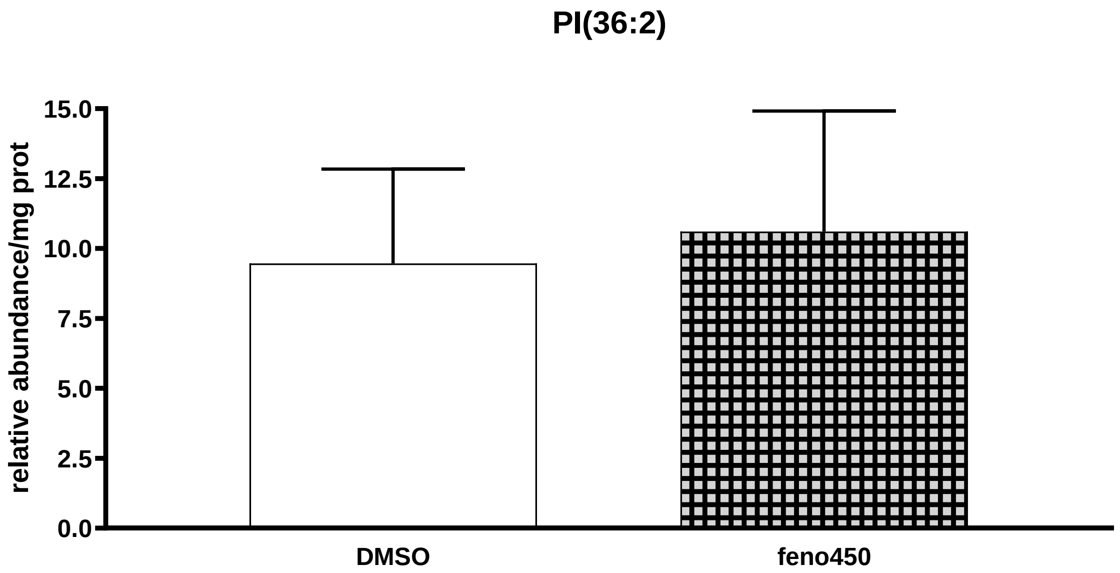
PI(36:2)
relative abundance/mg prot
15.0
12.5
10.0
7.5
5.0
2.5
0.0
DMSO
feno450

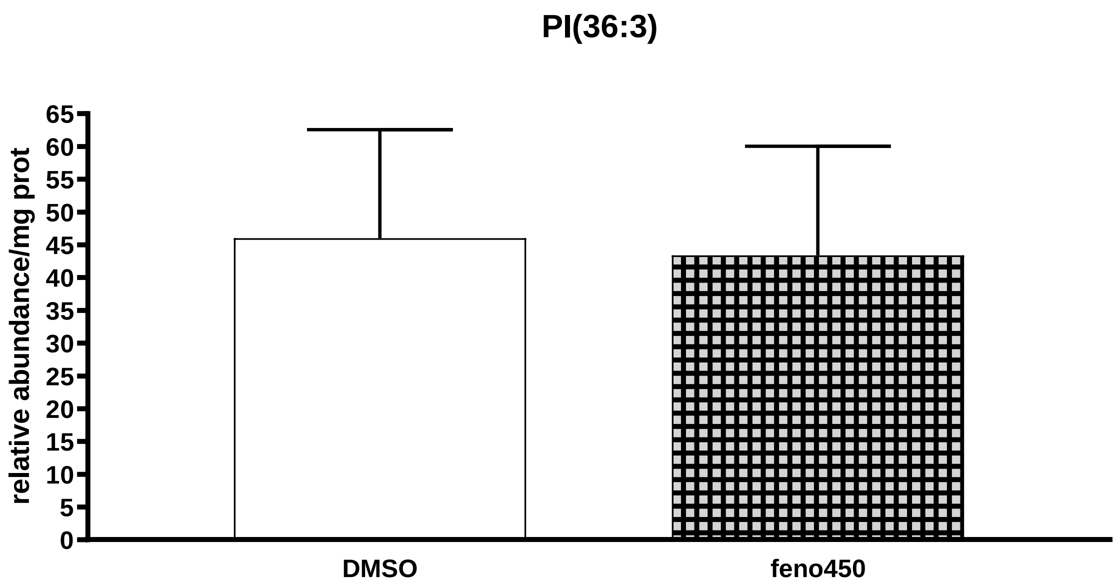
PI(36:3)
relative abundance/mg prot
65
60
55
50
45
40
35
30
25
20
15
10
5
0
DMSO
feno450

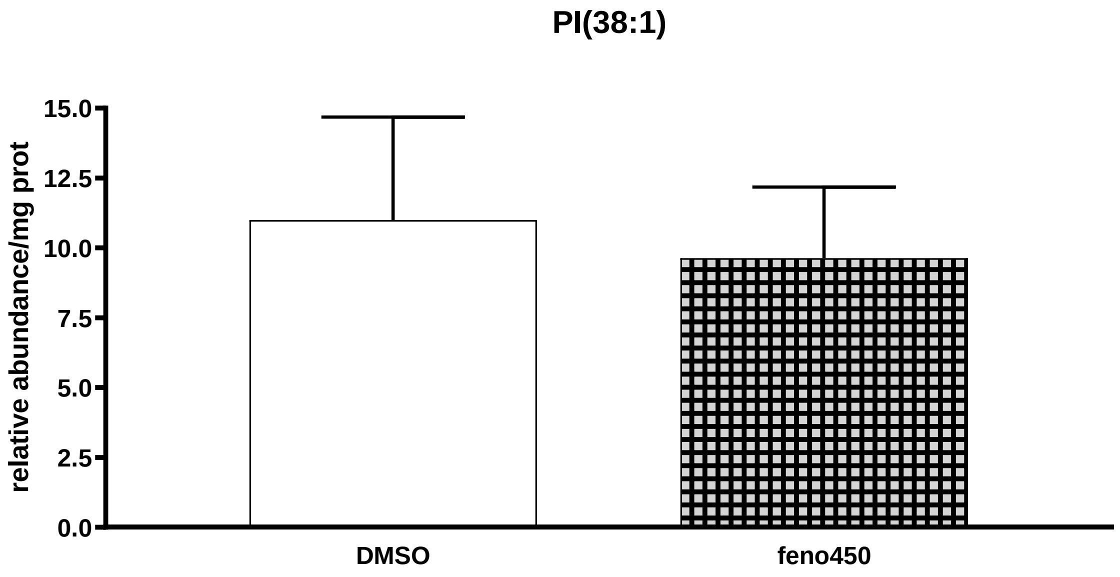
PI(38:1)
relative abundance/mg prot
15.0
12.5
10.0
7.5
5.0
2.5
0.0
DMSO
feno450

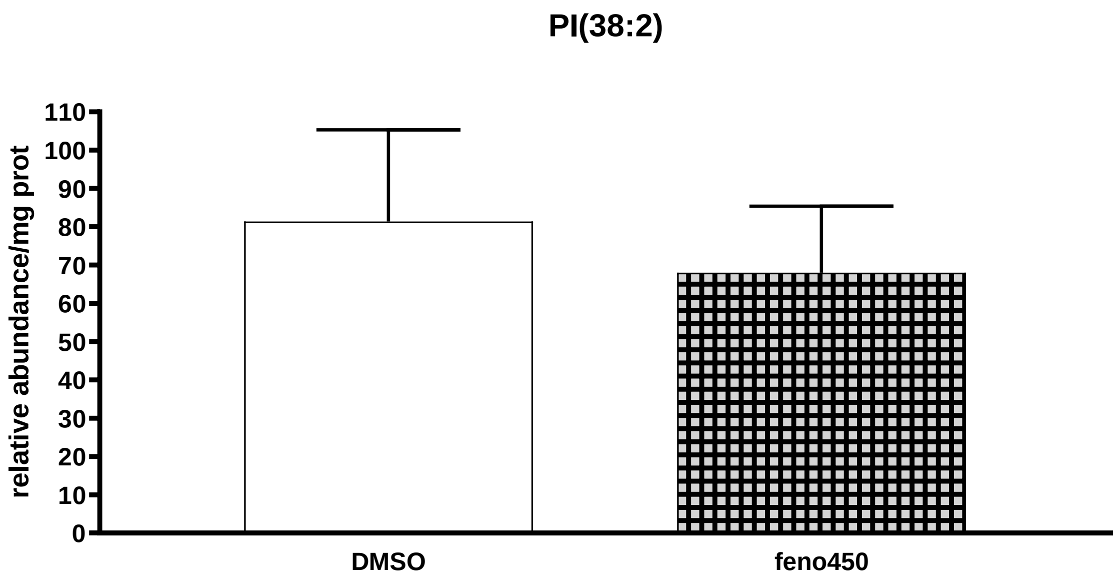
PI(38:2)
relative abundance/mg prot
110
100
90
80
70
60
50
40
30
20
10
0
DMSO
feno450

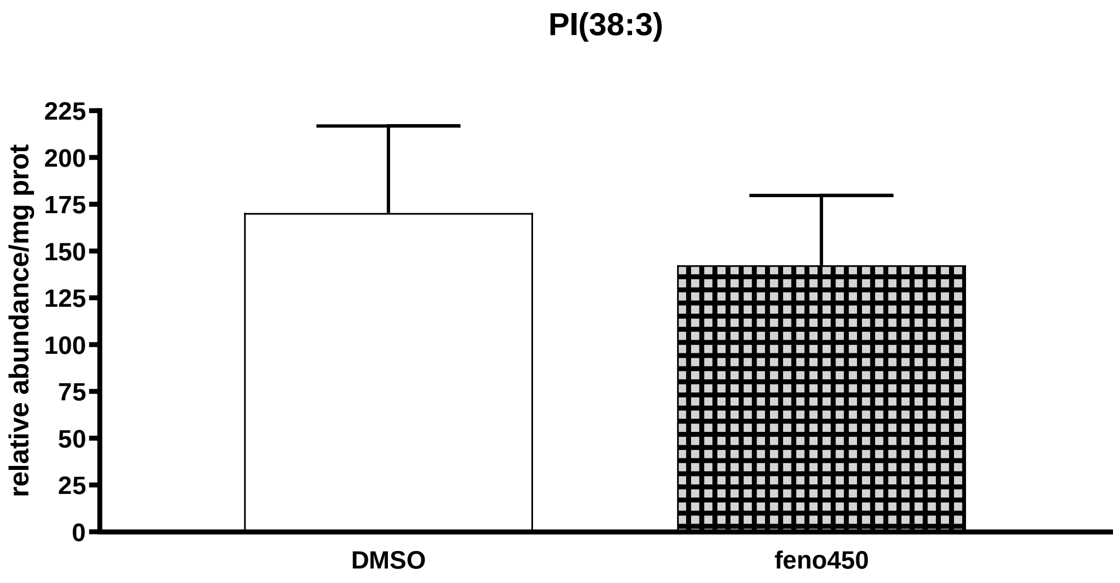
PI(38:3)
relative abundance/mg prot
225
200
175
150
125
100
75
50
25
0
DMSO
feno450

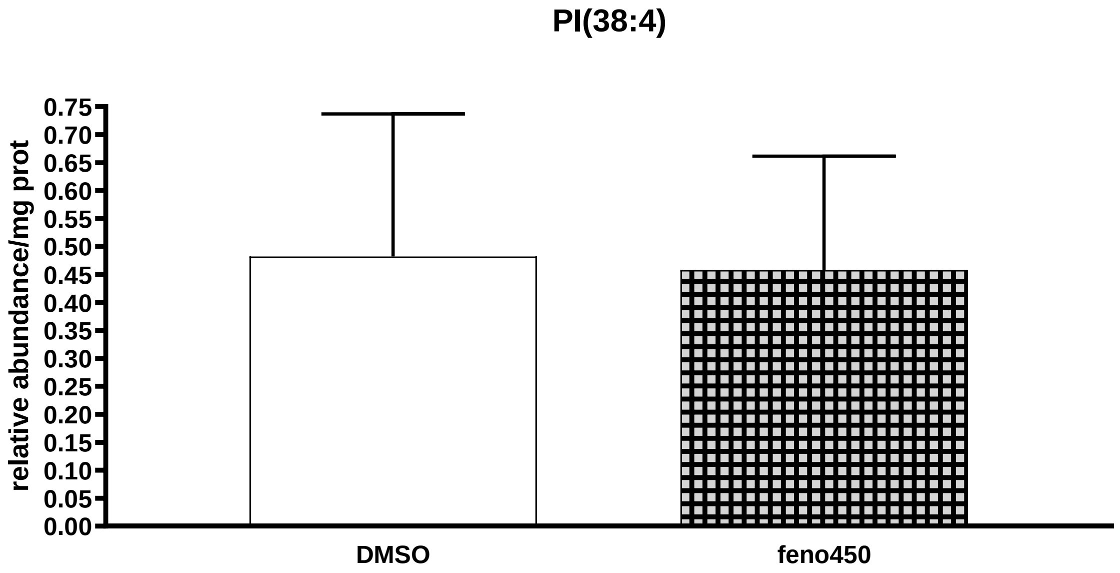
PI(38:4)
relative abundance/mg prot
0.75
0.70
0.65
0.60
0.55
0.50
0.45
0.40
0.35
0.30
0.25
0.20
0.15
0.10
0.05
0.00
DMSO
feno450

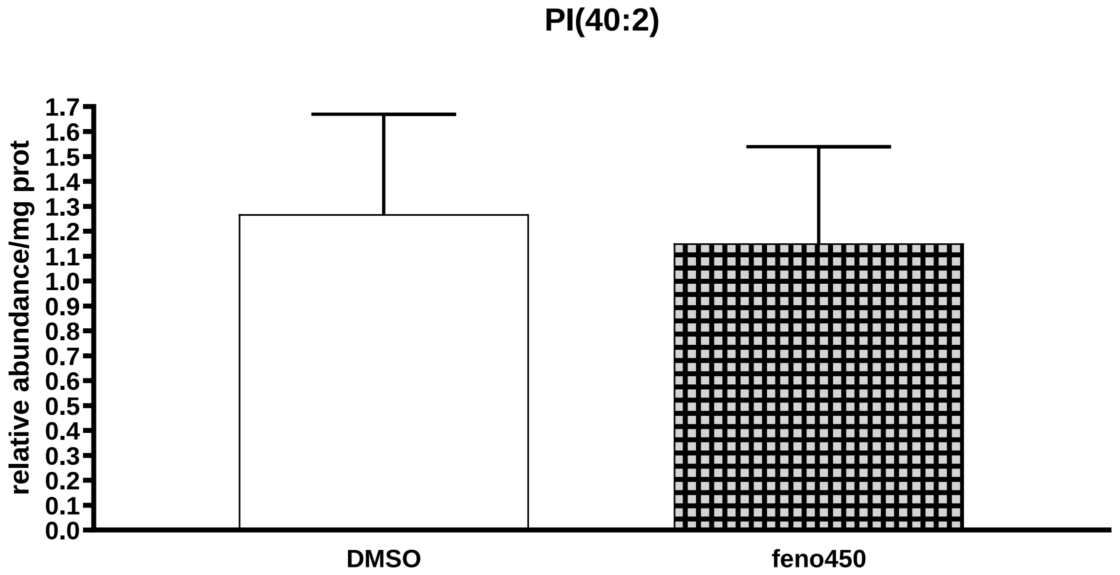
PI(40:2)
relative abundance/mg prot
1.7
1.6
1.5
1.4
1.3
1.2
1.1
1.0
0.9
0.8
0.7
0.6
0.5
0.4
0.3
0.2
0.1
0.0
DMSO
feno450

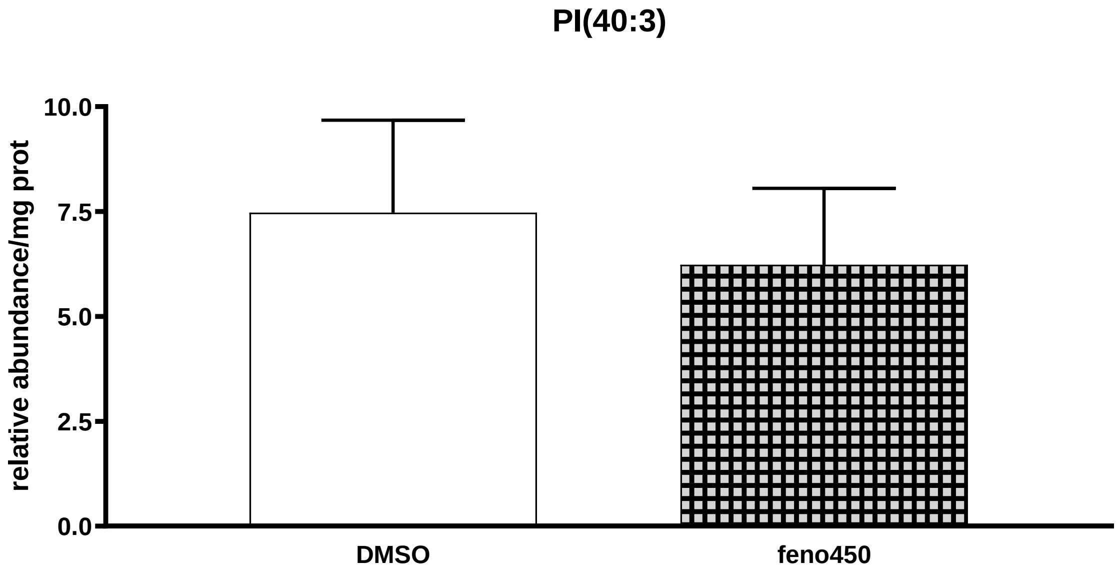
PI(40:3)
relative abundance/mg prot
10.0
7.5
5.0
2.5
0.0
DMSO
feno450

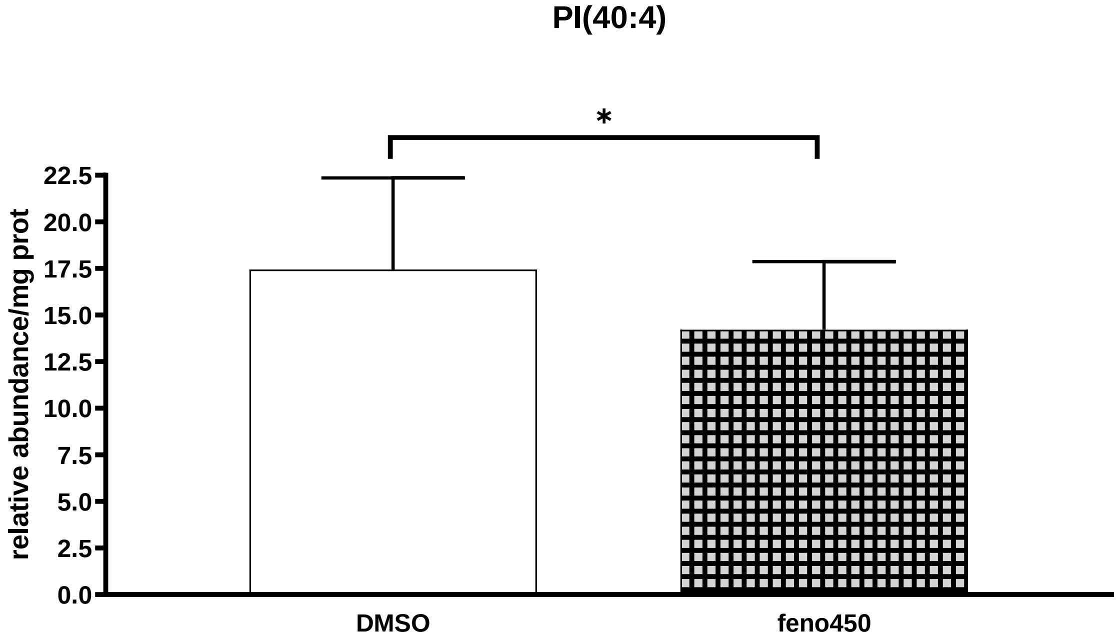
PI(40:4)
*
relative abundance/mg prot
22.5
20.0
17.5
15.0
12.5
10.0
7.5
5.0
2.5
0.0
DMSO
feno450

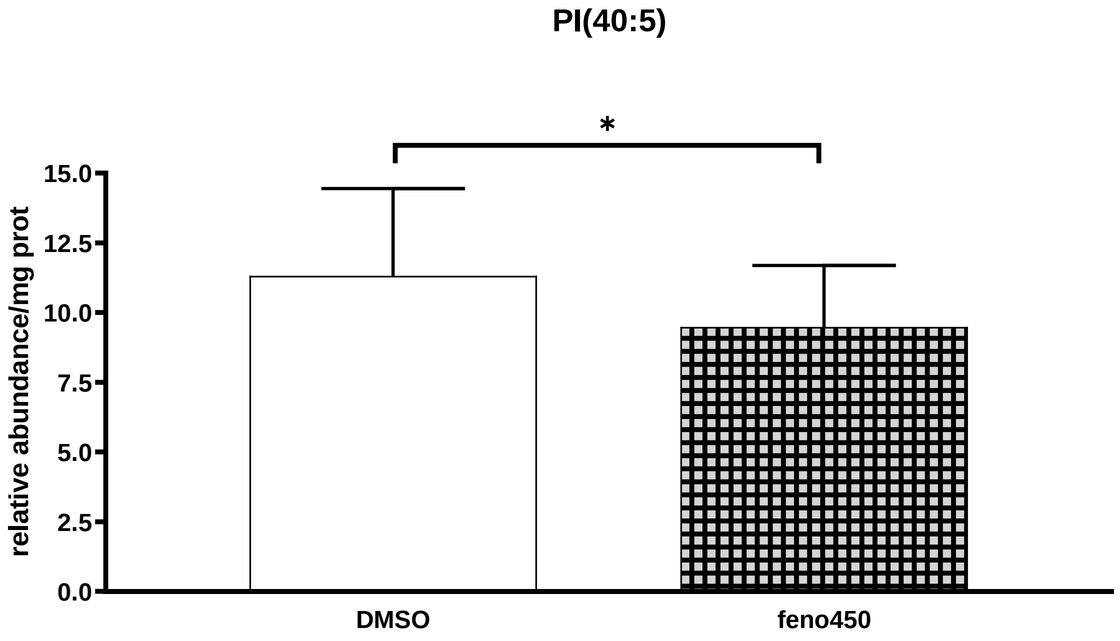
PI(40:5)
*
relative abundance/mg prot
15.0
12.5
10.0
7.5
5.0
2.5
0.0
DMSO
feno450

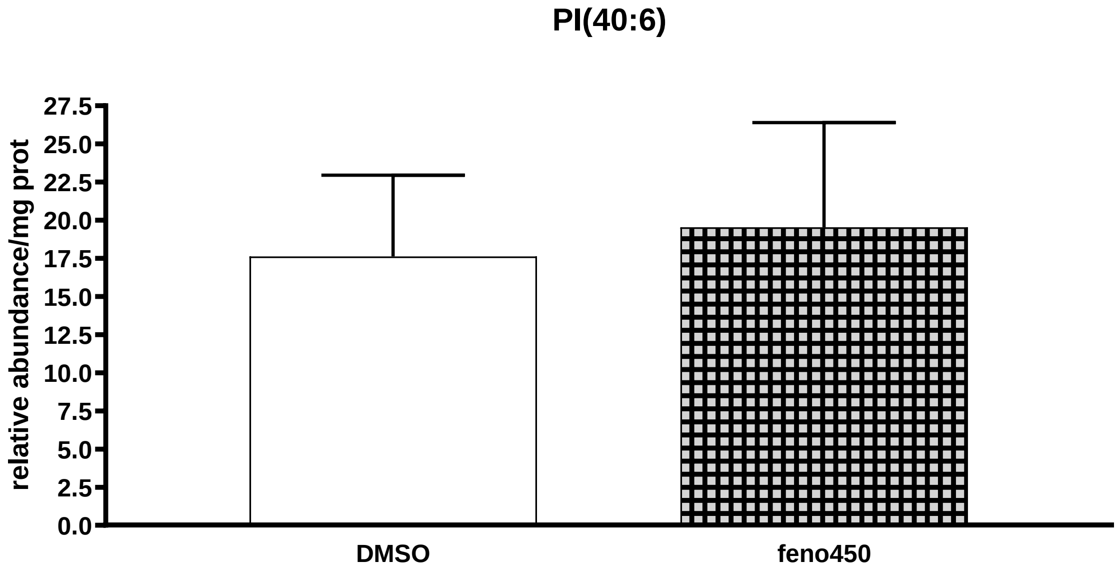
PI(40:6)
relative abundance/mg prot
27.5
25.0
22.5
20.0
17.5
15.0
12.5
10.0
7.5
5.0
2.5
0.0
DMSO
feno450

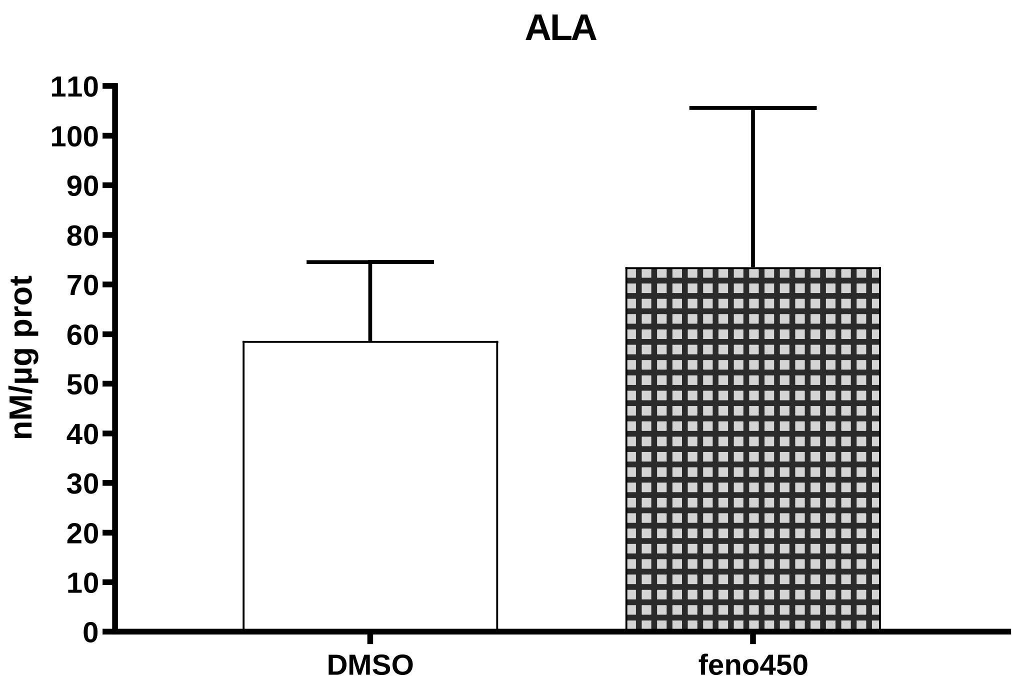
ALA
nM/µg prot
110
100
90
80
70
60
50
40
30
20
10
0
DMSO
feno450

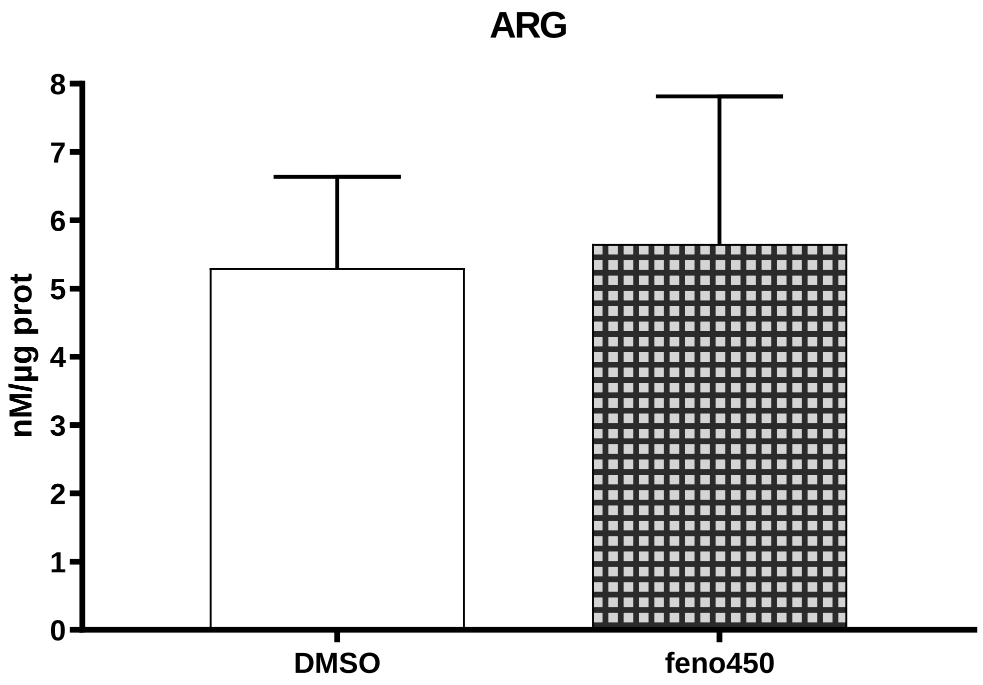
ARG
nM/µg prot
8
7
6
5
4
3
2
1
0
DMSO
feno450

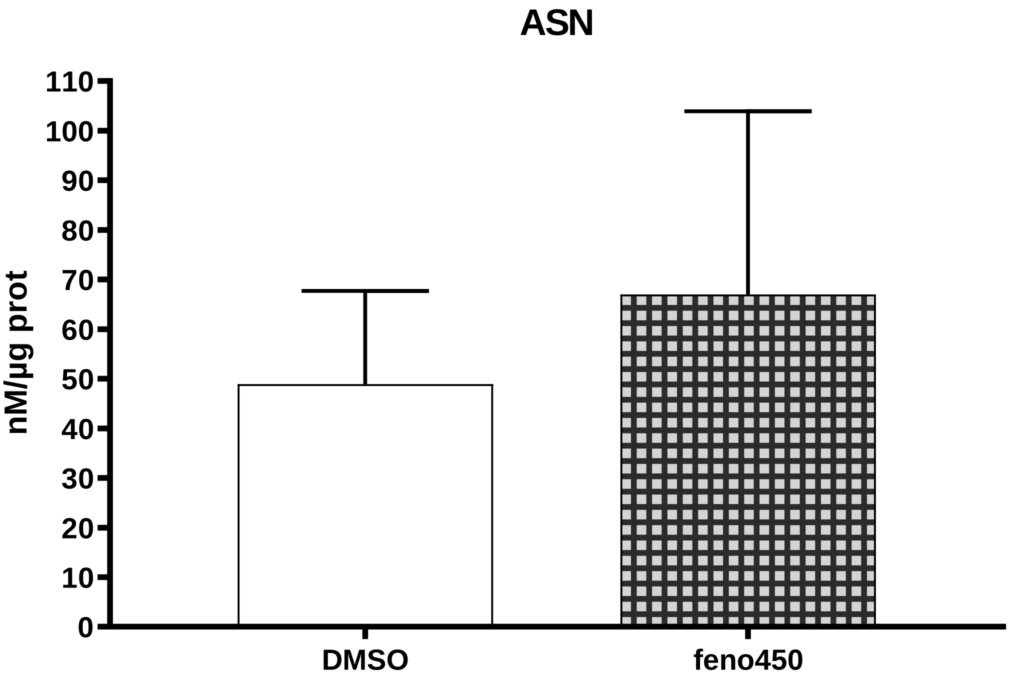

ASN
nM/µg prot
110
100
90
80
70
60
50
40
30
20
10
0
DMSO
feno450

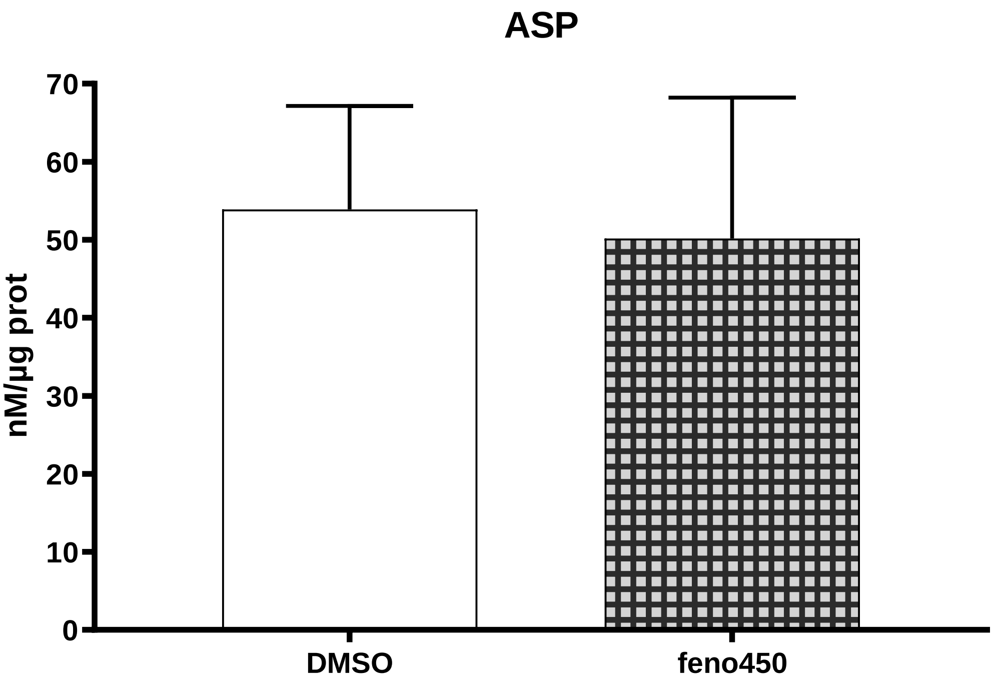

ASP
nM/µg prot
70
60
50
40
30
20
10
0
DMSO
feno450

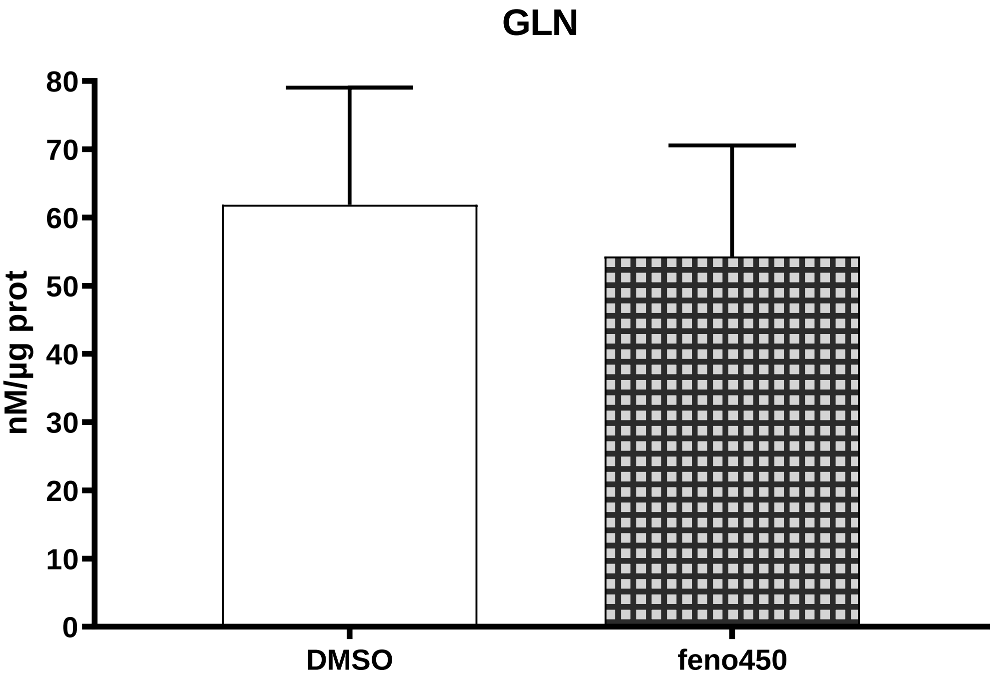
GLN
nM/µg prot
0
10
20
30
40
50
60
70
80
DMSO
feno450

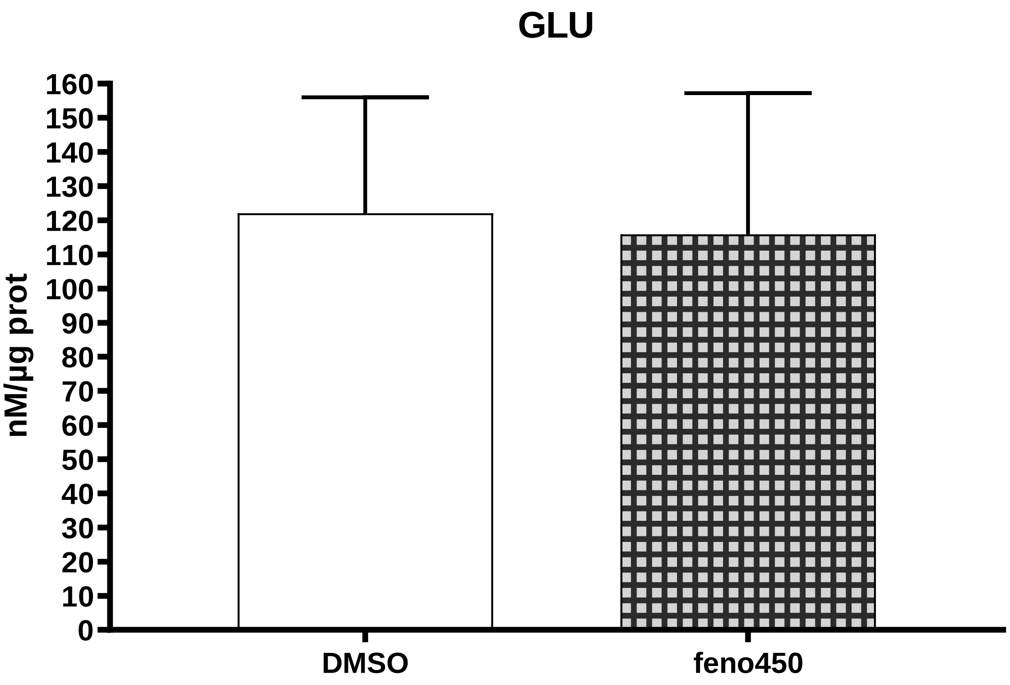
GLU
nM/µg prot
0
10
20
30
40
50
60
70
80
90
100
110
120
130
140
150
160
DMSO
feno450

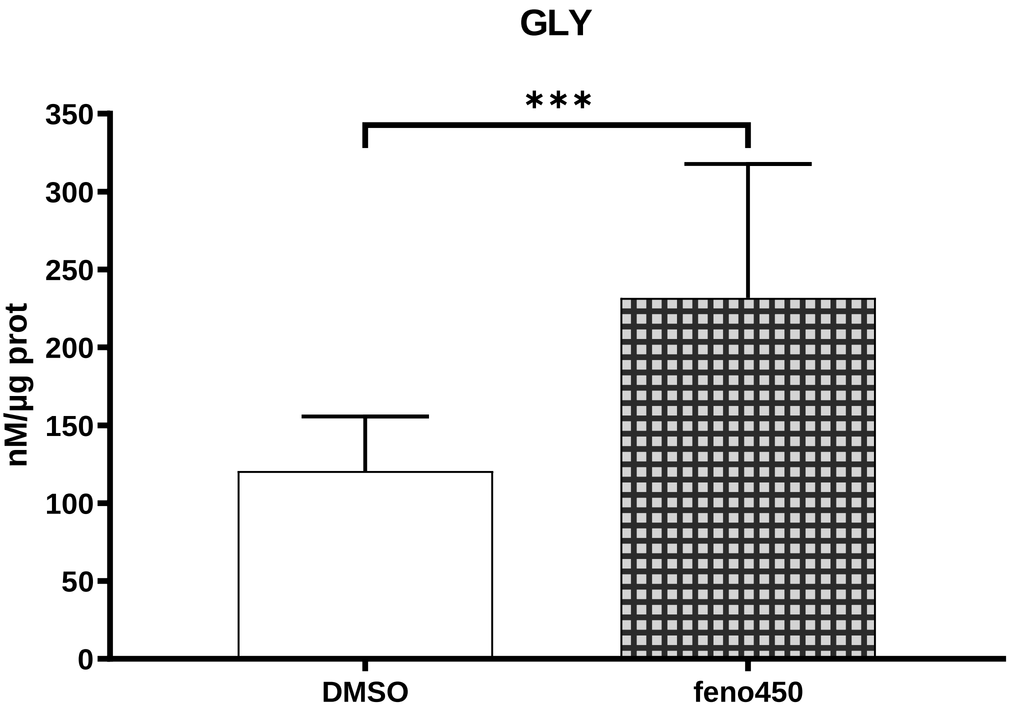
GLY
***
350
300
250
200
150
100
50
0
nM/µg prot
DMSO
feno450

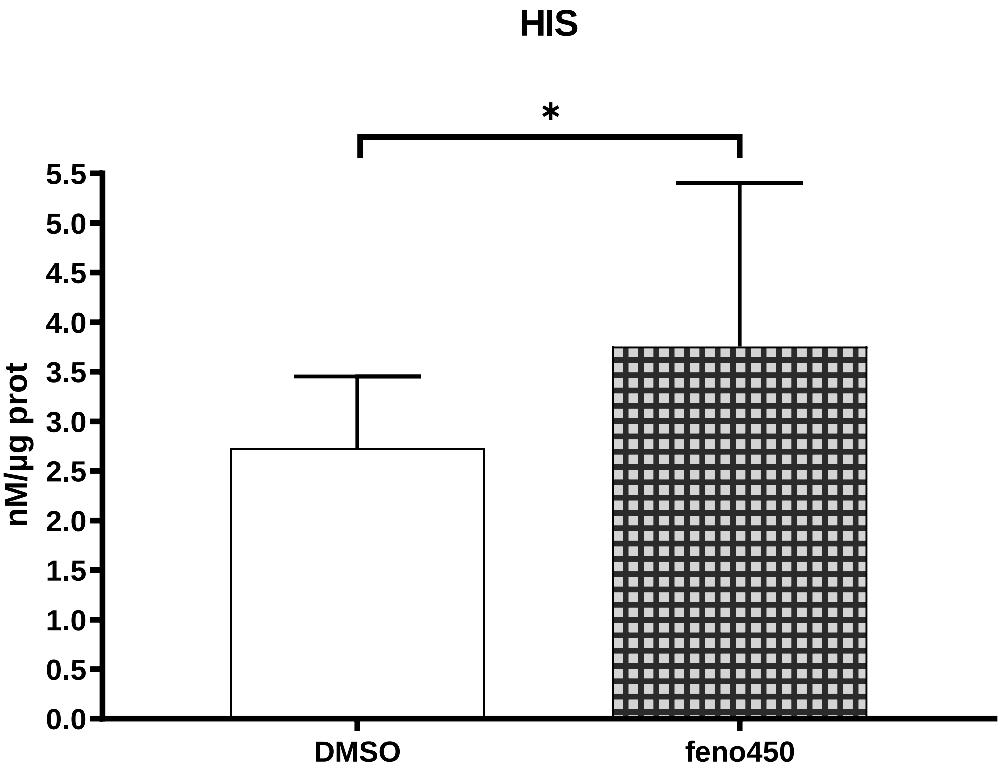
HIS
*
5.5
5.0
4.5
4.0
3.5
3.0
2.5
2.0
1.5
1.0
0.5
0.0
nM/µg prot
DMSO
feno450

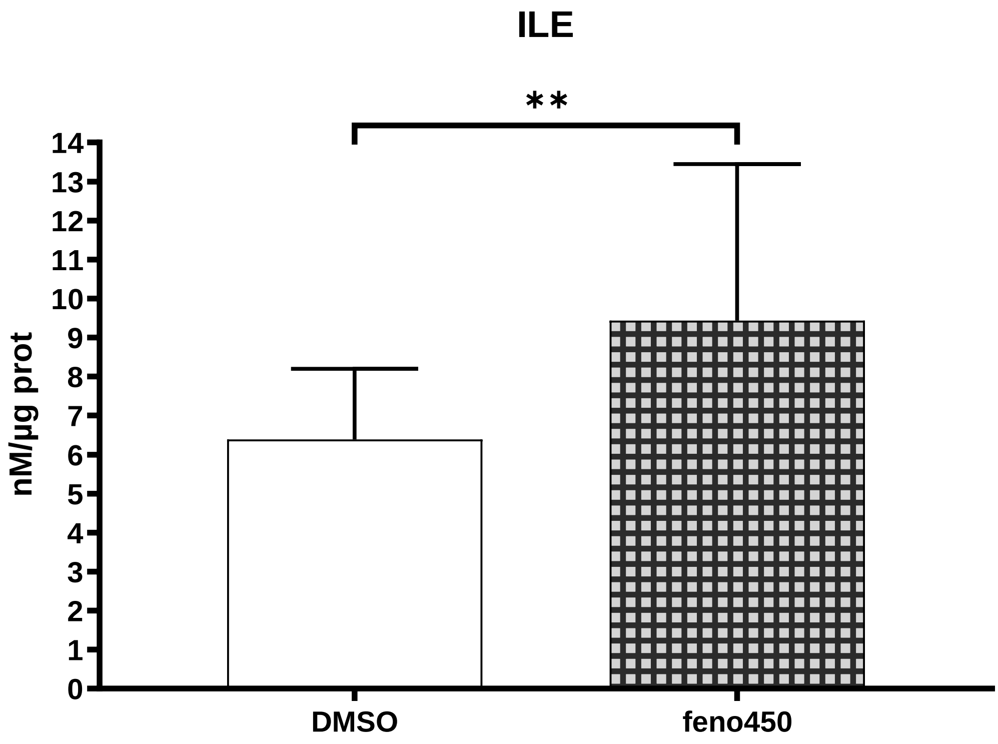
ILE
**
nM/µg prot
0
1
2
3
4
5
6
7
8
9
10
11
12
13
14
DMSO
feno450

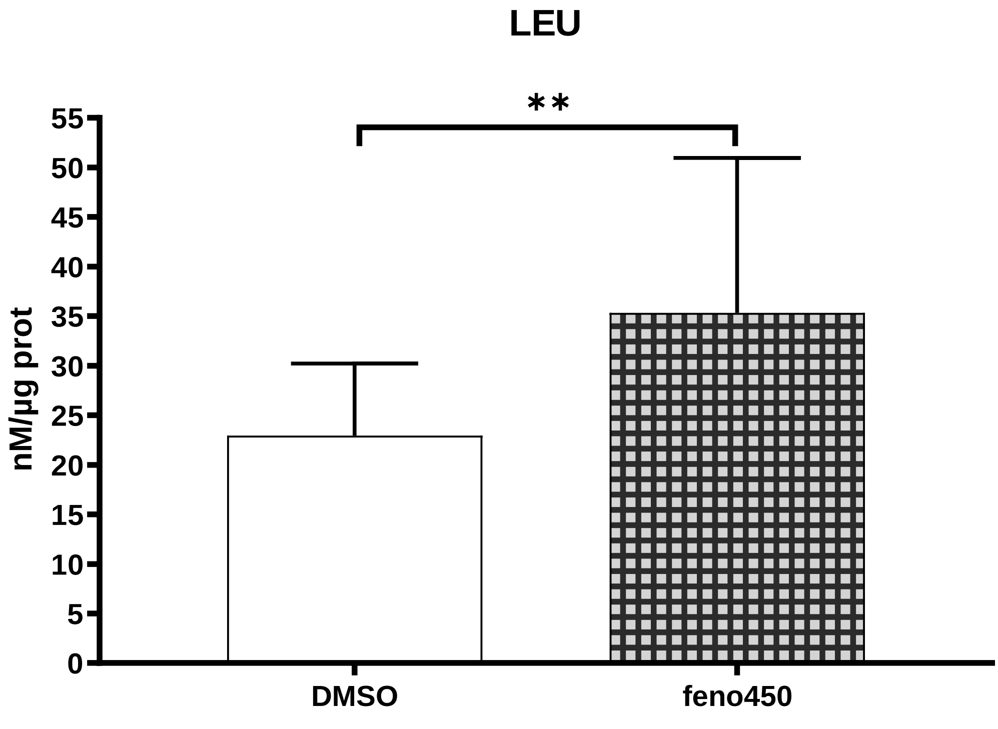
LEU
**
nM/µg prot
0
5
10
15
20
25
30
35
40
45
50
55
DMSO
feno450

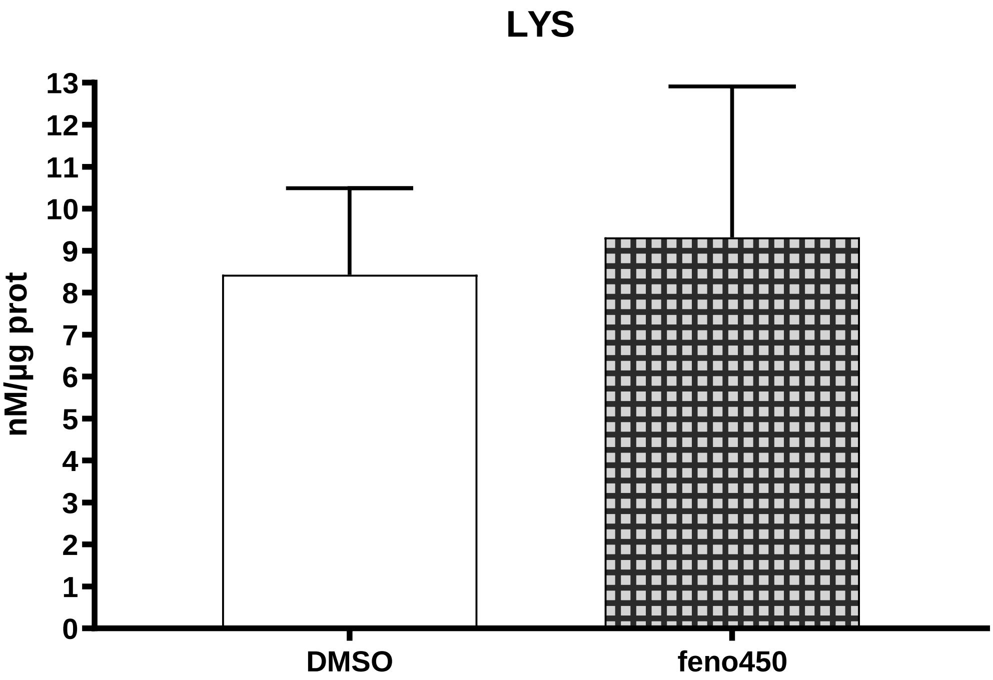
LYS
nM/µg prot
13
12
11
10
9
8
7
6
5
4
3
2
1
0
DMSO
feno450

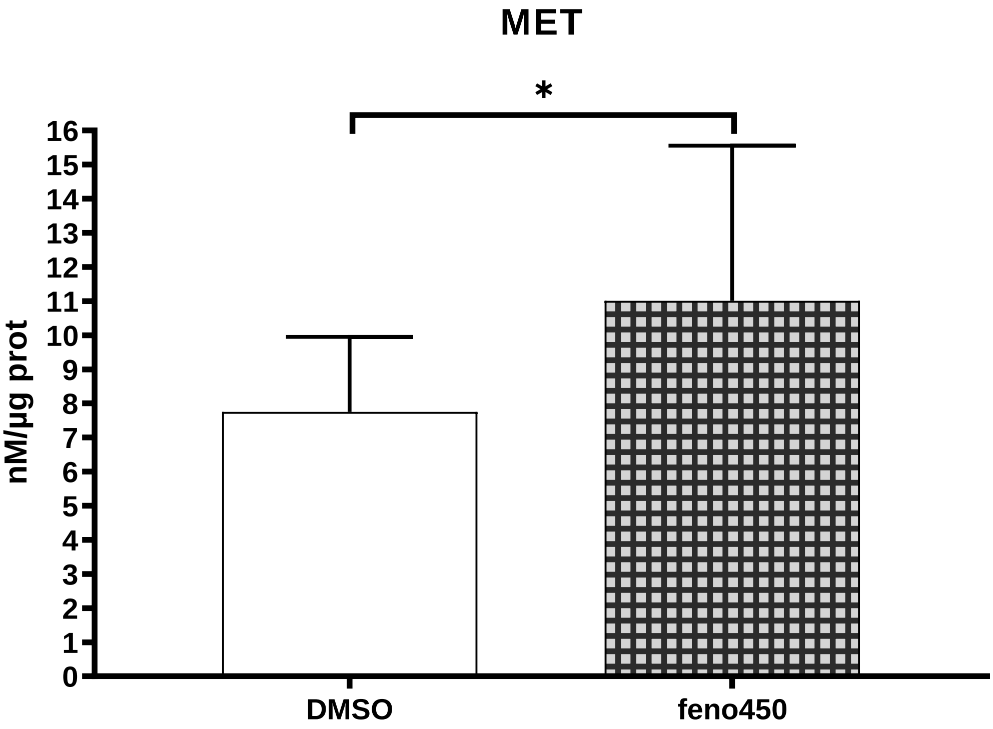
MET
*
nM/µg prot
16
15
14
13
12
11
10
9
8
7
6
5
4
3
2
1
0
DMSO
feno450

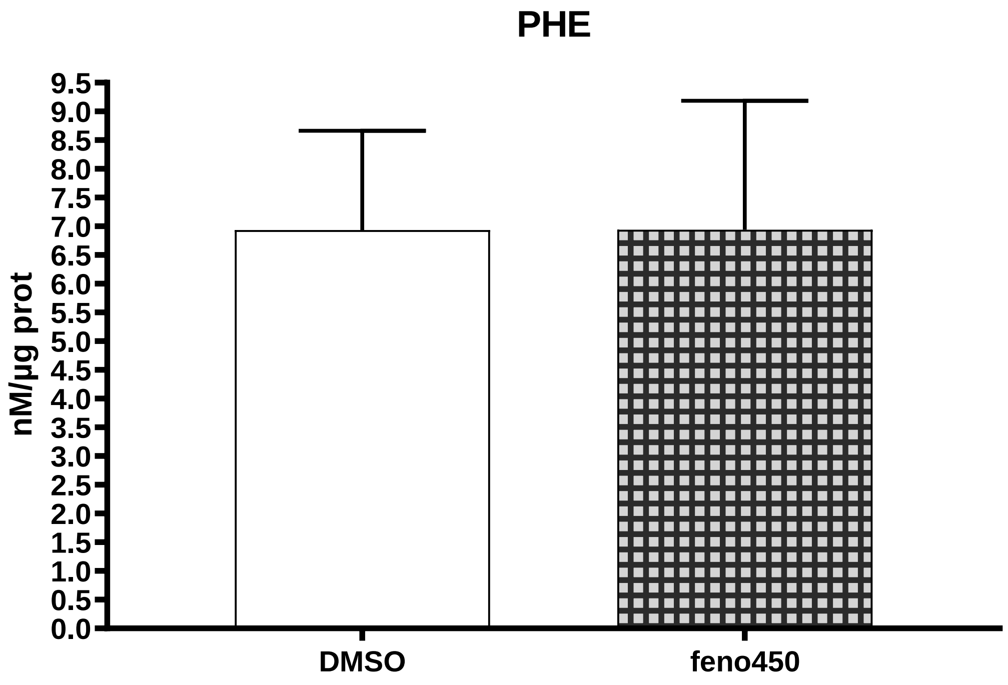
PHE
nM/µg prot
9.5
9.0
8.5
8.0
7.5
7.0
6.5
6.0
5.5
5.0
4.5
4.0
3.5
3.0
2.5
2.0
1.5
1.0
0.5
0.0
DMSO
feno450

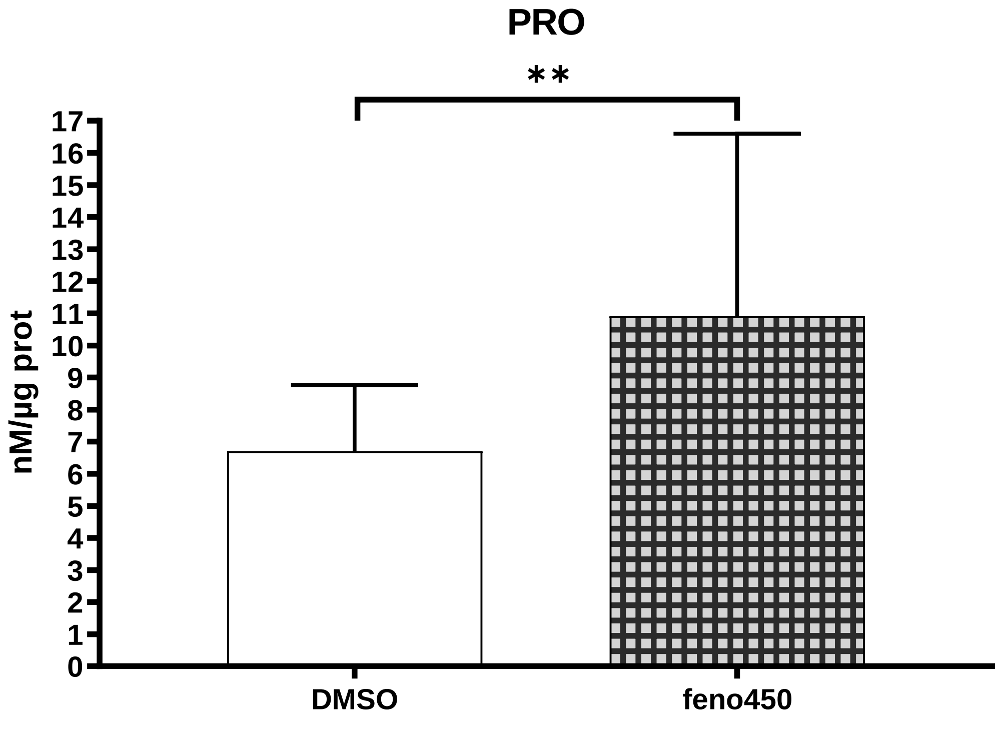
PRO
**
nM/µg prot
17
16
15
14
13
12
11
10
9
8
7
6
5
4
3
2
1
0
DMSO
feno450

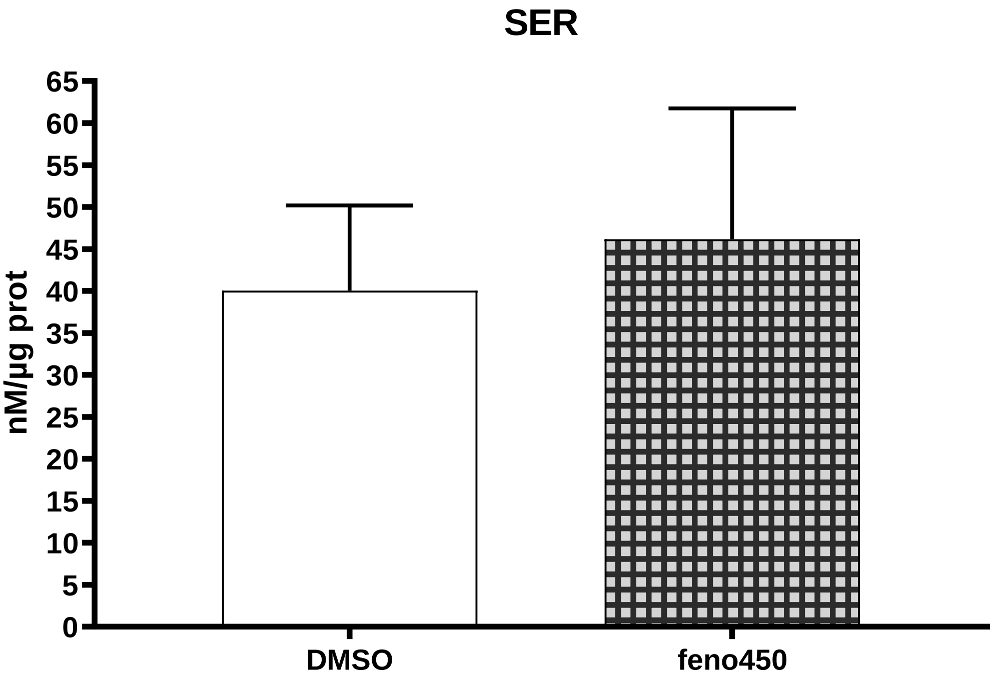
SER
65
60
55
50
45
40
35
30
25
20
15
10
5
0
nM/µg prot
DMSO
feno450

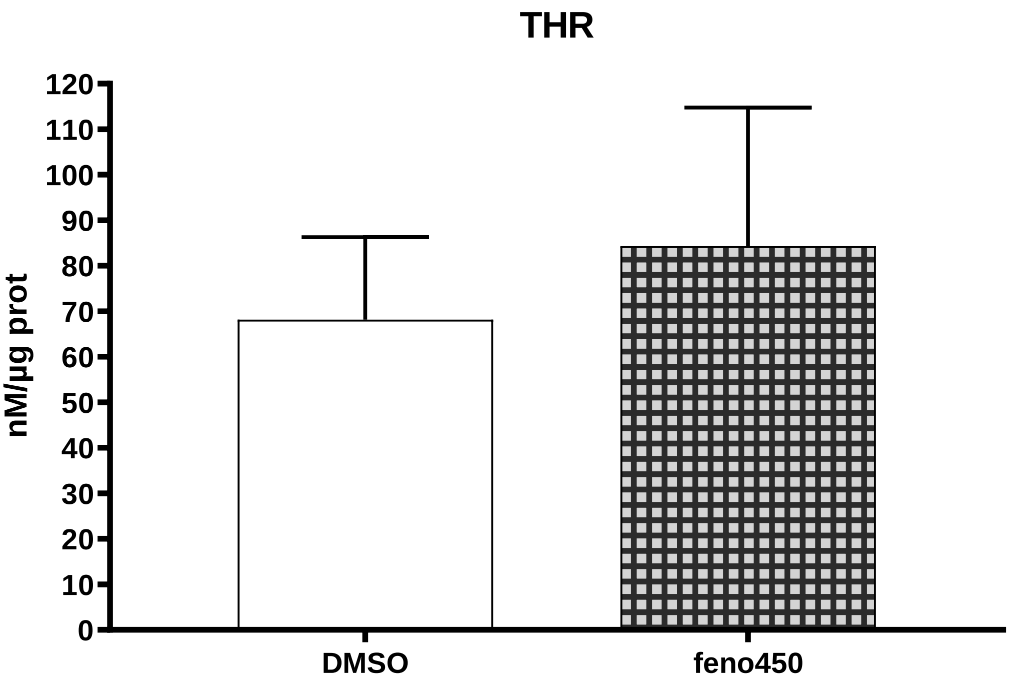
THR
120
110
100
90
80
70
60
50
40
30
20
10
0
nM/µg prot
DMSO
feno450

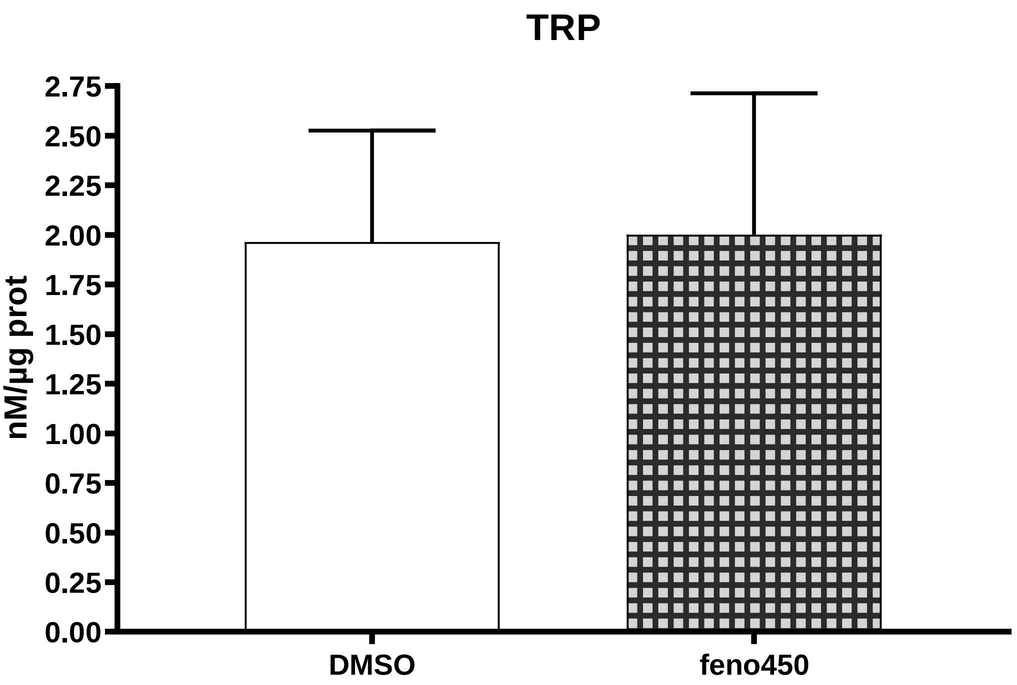
TRP
nM/µg prot
2.75
2.50
2.25
2.00
1.75
1.50
1.25
1.00
0.75
0.50
0.25
0.00
DMSO
feno450

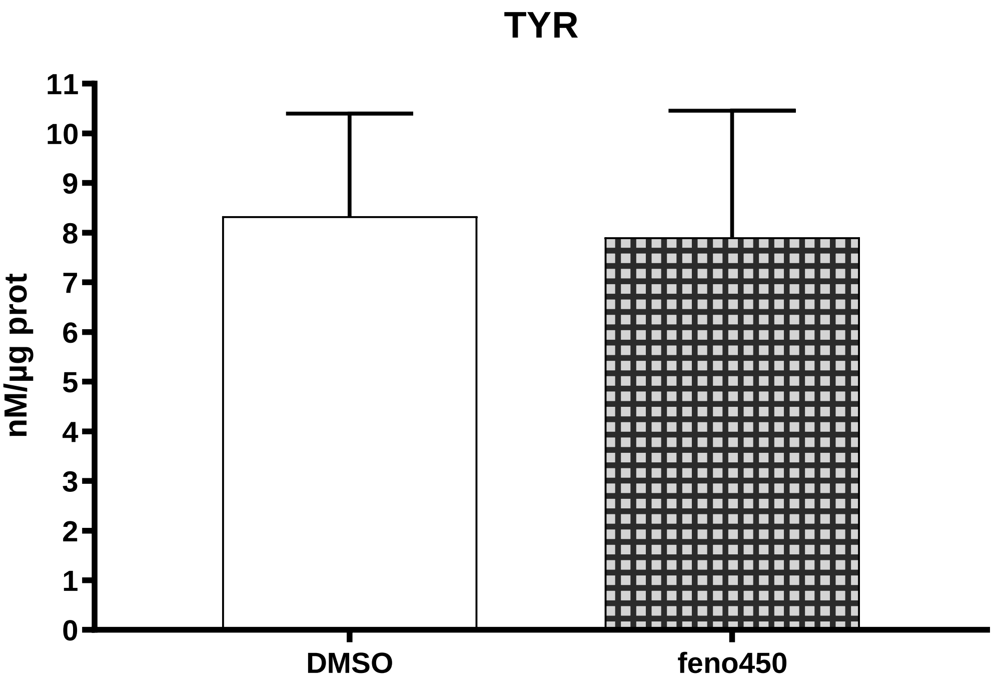
TYR
nM/µg prot
11
10
9
8
7
6
5
4
3
2
1
0
DMSO
feno450

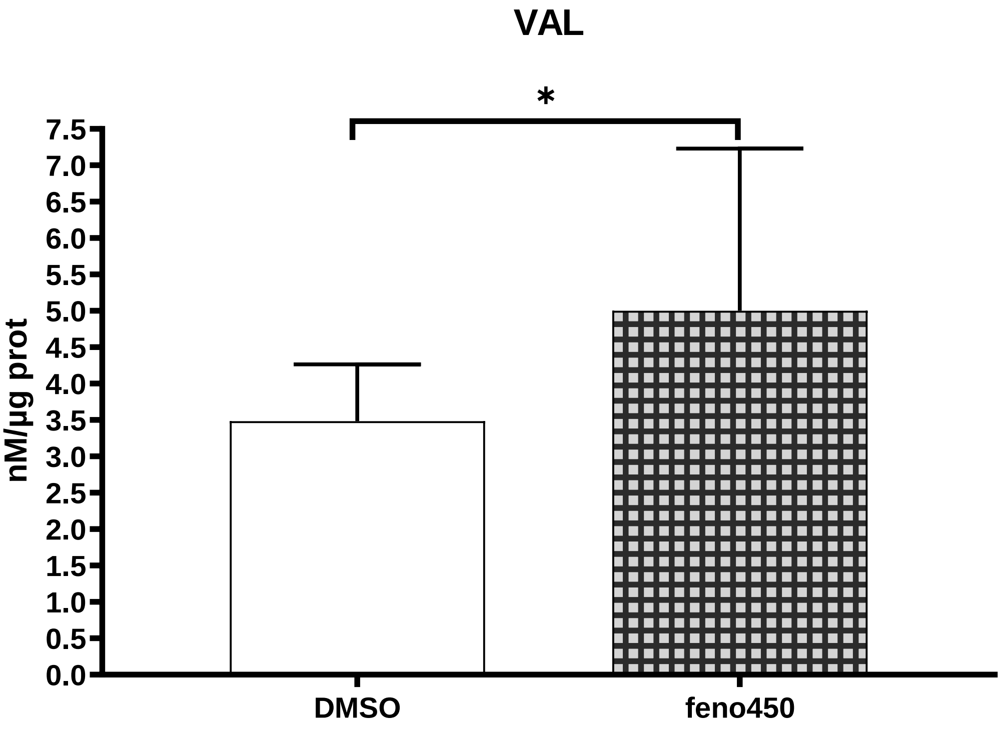
VAL
*
nM/µg prot
7.5
7.0
6.5
6.0
5.5
5.0
4.5
4.0
3.5
3.0
2.5
2.0
1.5
1.0
0.5
0.0
DMSO
feno450

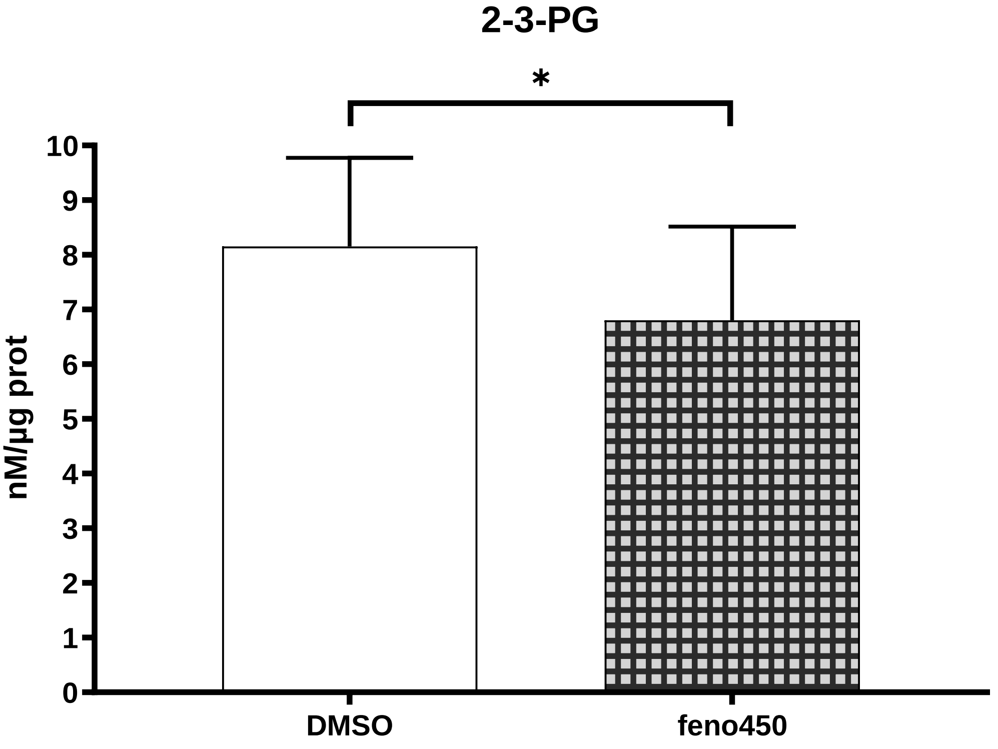
2-3-PG
*
nM/µg prot
10
9
8
7
6
5
4
3
2
1
0
DMSO
feno450

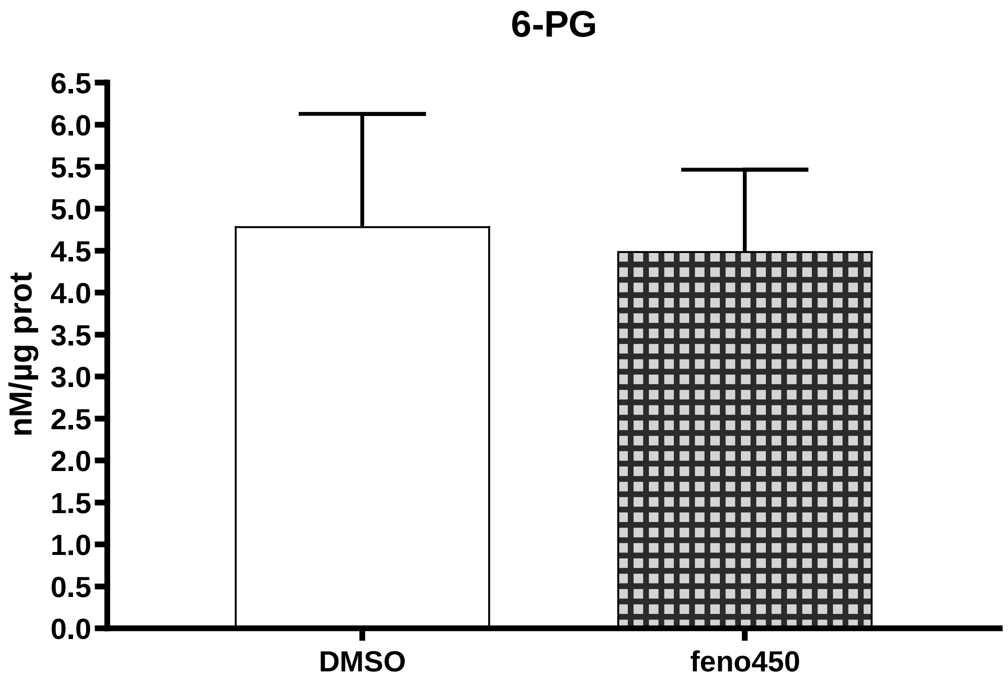
6-PG
nM/µg prot
6.5
6.0
5.5
5.0
4.5
4.0
3.5
3.0
2.5
2.0
1.5
1.0
0.5
0.0
DMSO
feno450

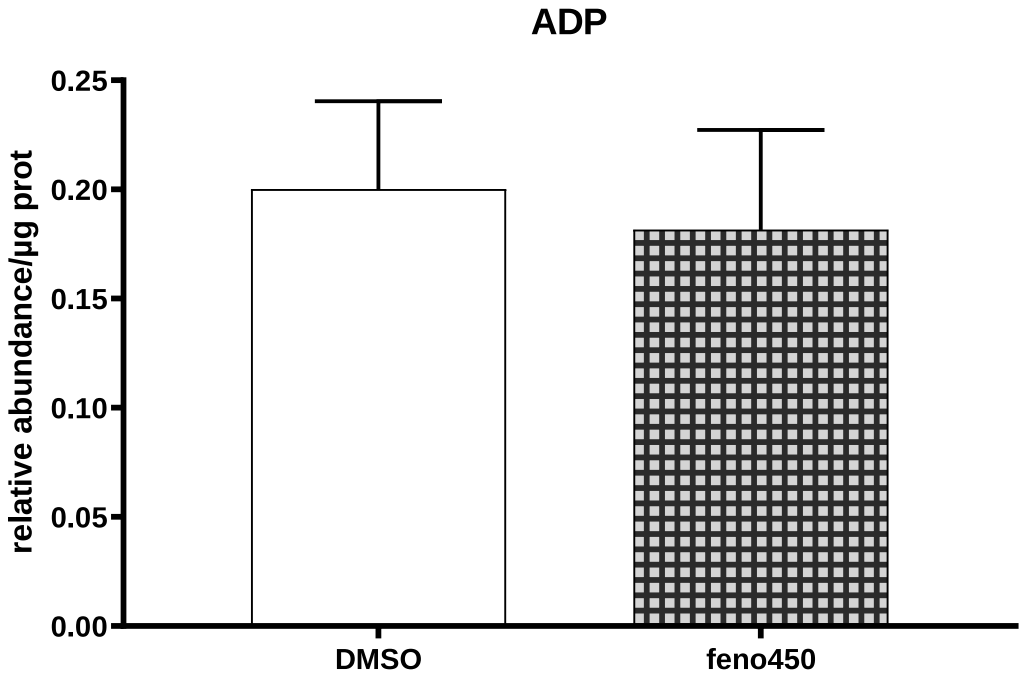
ADP
relative abundance/µg prot
0.25
0.20
0.15
0.10
0.05
0.00
DMSO
feno450

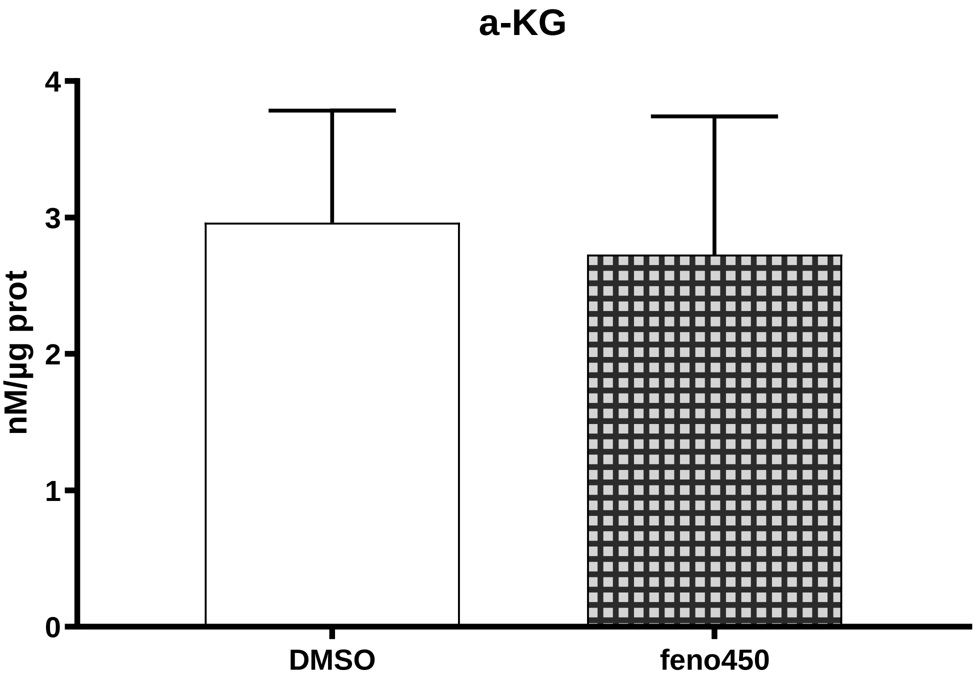
a-KG
nM/µg prot
4
3
2
1
0
DMSO
feno450

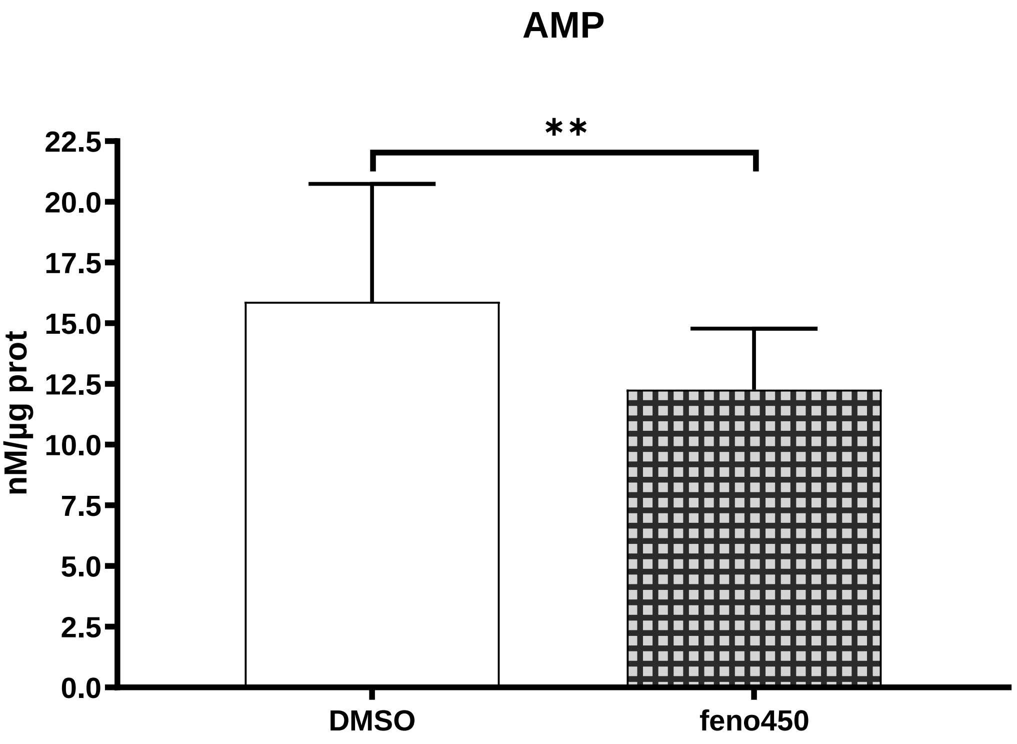
AMP
**
nM/µg prot
22.5
20.0
17.5
15.0
12.5
10.0
7.5
5.0
2.5
0.0
DMSO
feno450

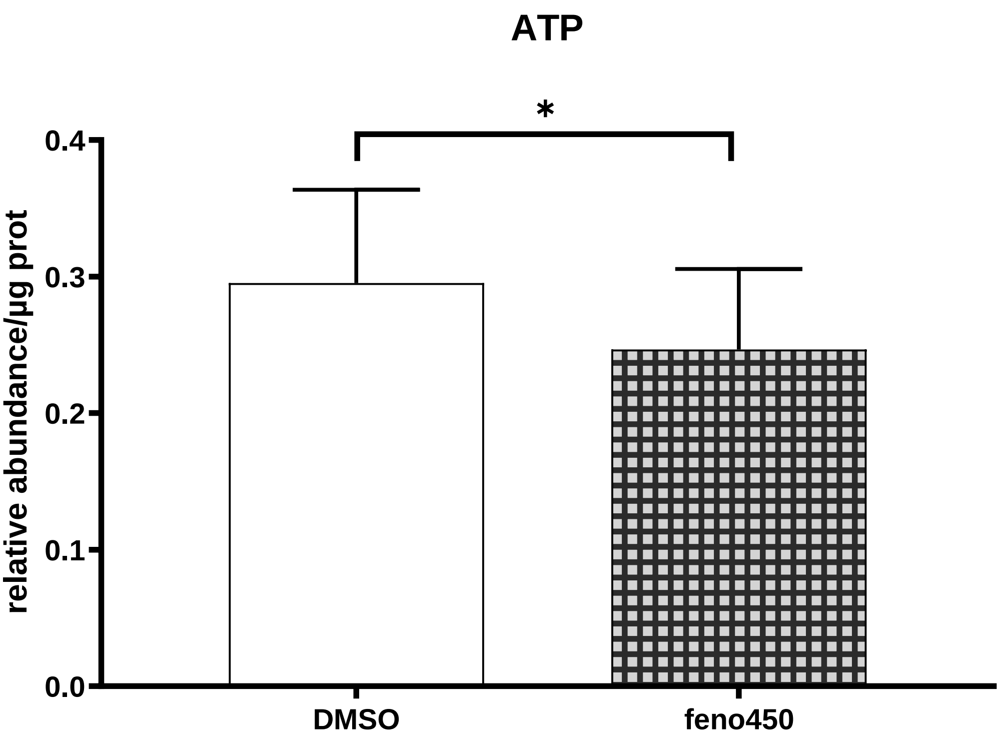
ATP
*
0.4
0.3
0.2
0.1
0.0
relative abundance/µg prot
DMSO
feno450

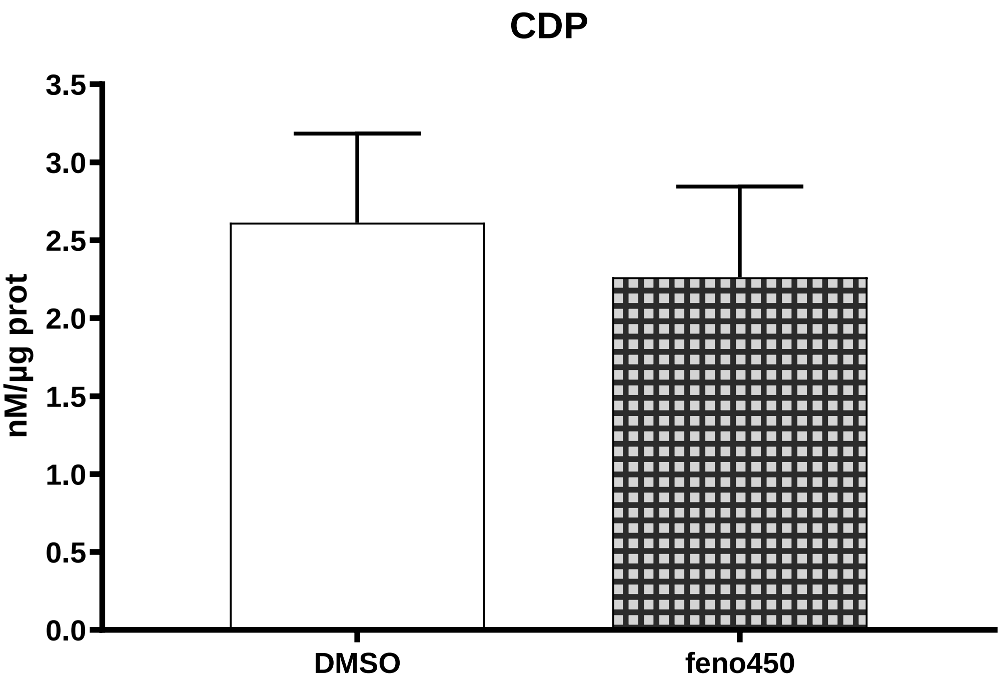
CDP
3.5
3.0
2.5
2.0
1.5
1.0
0.5
0.0
nM/µg prot
DMSO
feno450

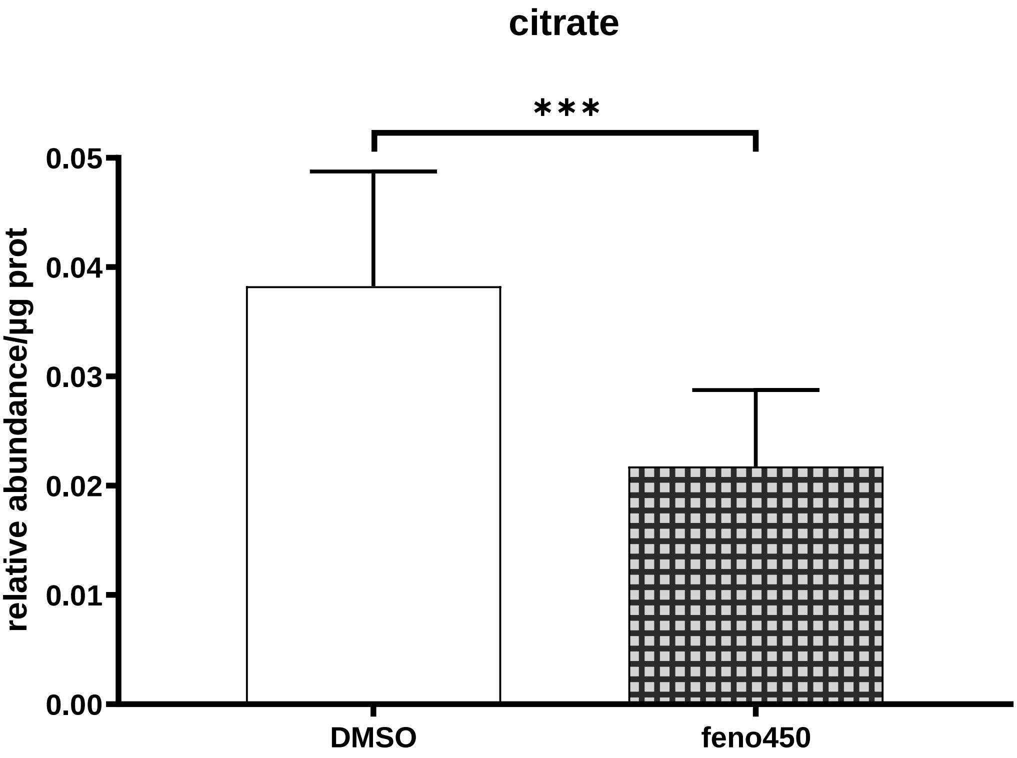
citrate
***
relative abundance/µg prot
0.05
0.04
0.03
0.02
0.01
0.00
DMSO
feno450

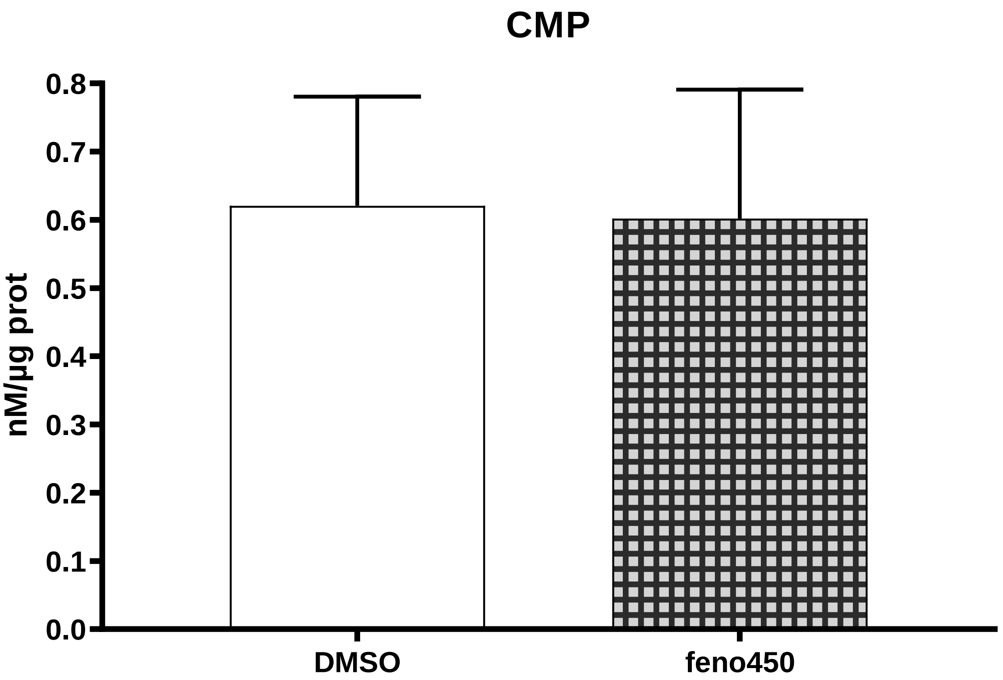
CMP
nM/µg prot
0.8
0.7
0.6
0.5
0.4
0.3
0.2
0.1
0.0
DMSO
feno450

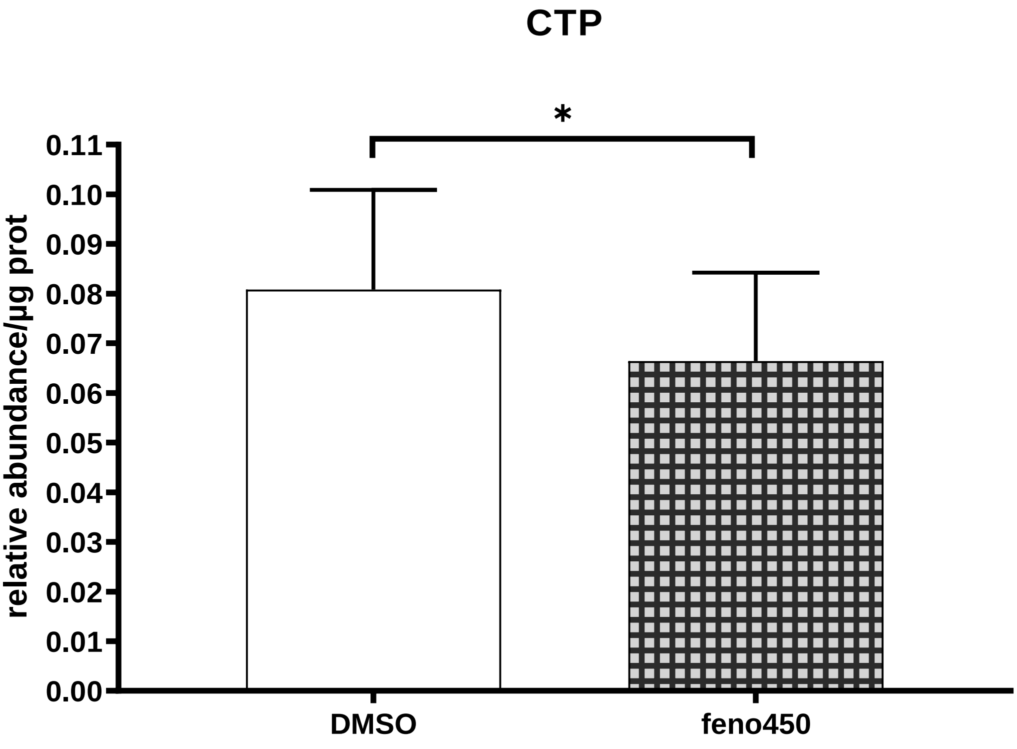
CTP
*
relative abundance/µg prot
0.11
0.10
0.09
0.08
0.07
0.06
0.05
0.04
0.03
0.02
0.01
0.00
DMSO
feno450

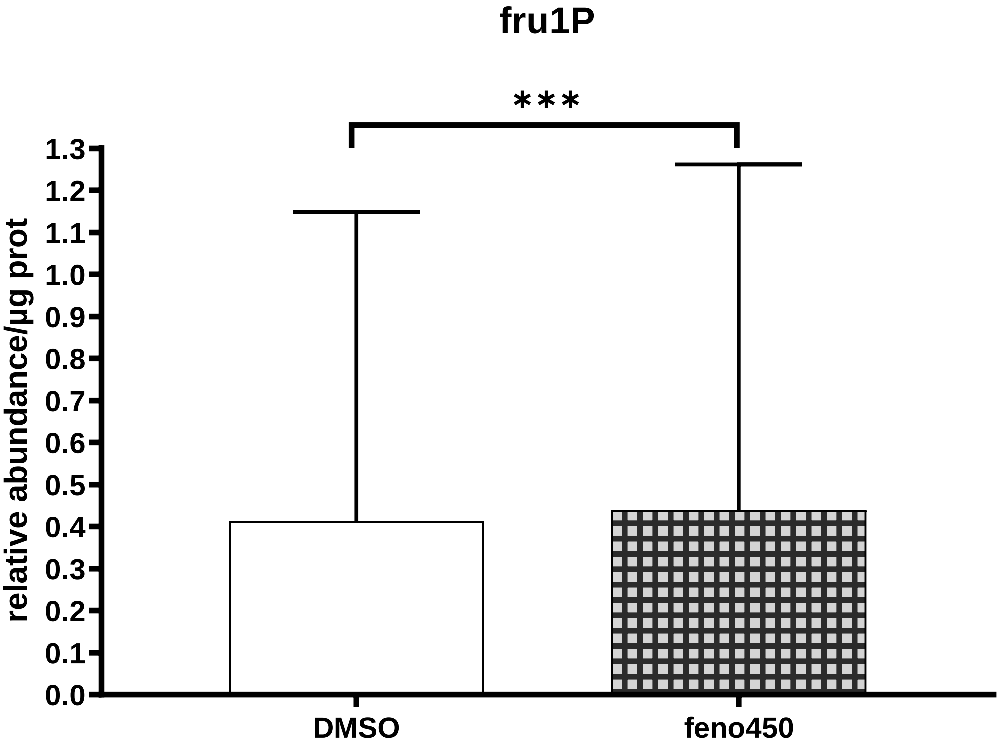
fru1P
***
relative abundance/µg prot
1.3
1.2
1.1
1.0
0.9
0.8
0.7
0.6
0.5
0.4
0.3
0.2
0.1
0.0
DMSO
feno450

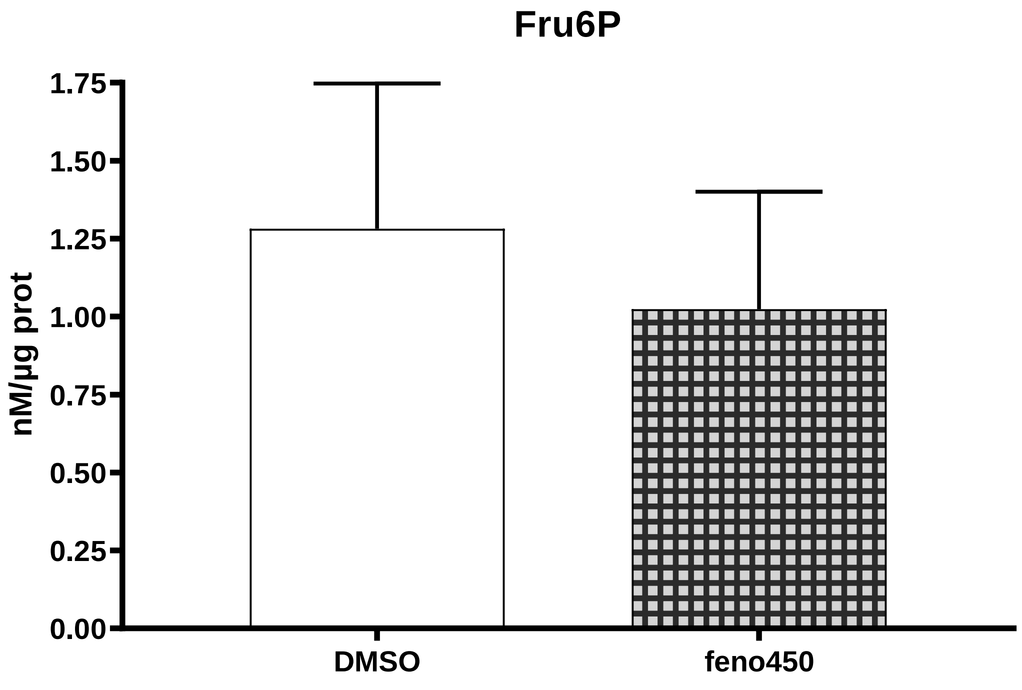
Fru6P
nM/µg prot
1.75
1.50
1.25
1.00
0.75
0.50
0.25
0.00
DMSO
feno450

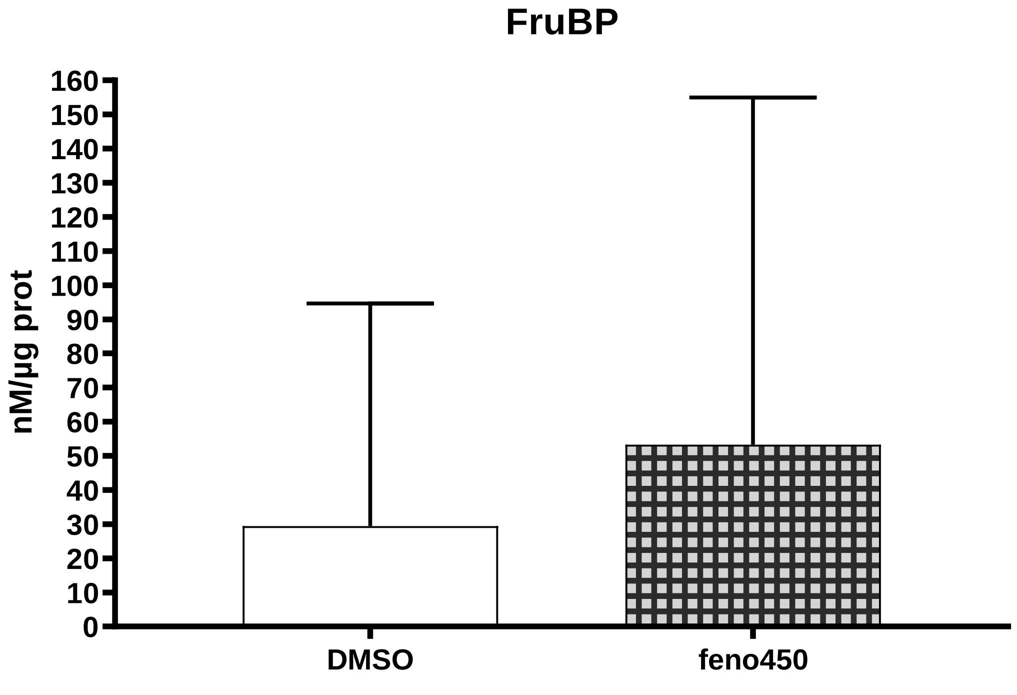
FruBP
nM/µg prot
160
150
140
130
120
110
100
90
80
70
60
50
40
30
20
10
0
DMSO
feno450

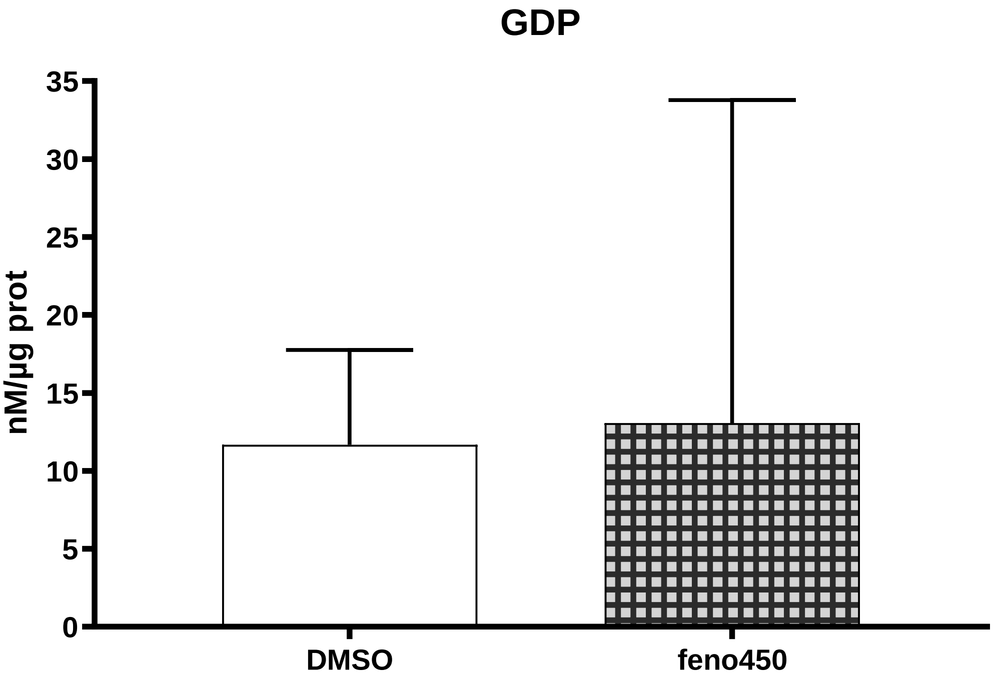
GDP
nM/µg prot
35
30
25
20
15
10
5
0
DMSO
feno450

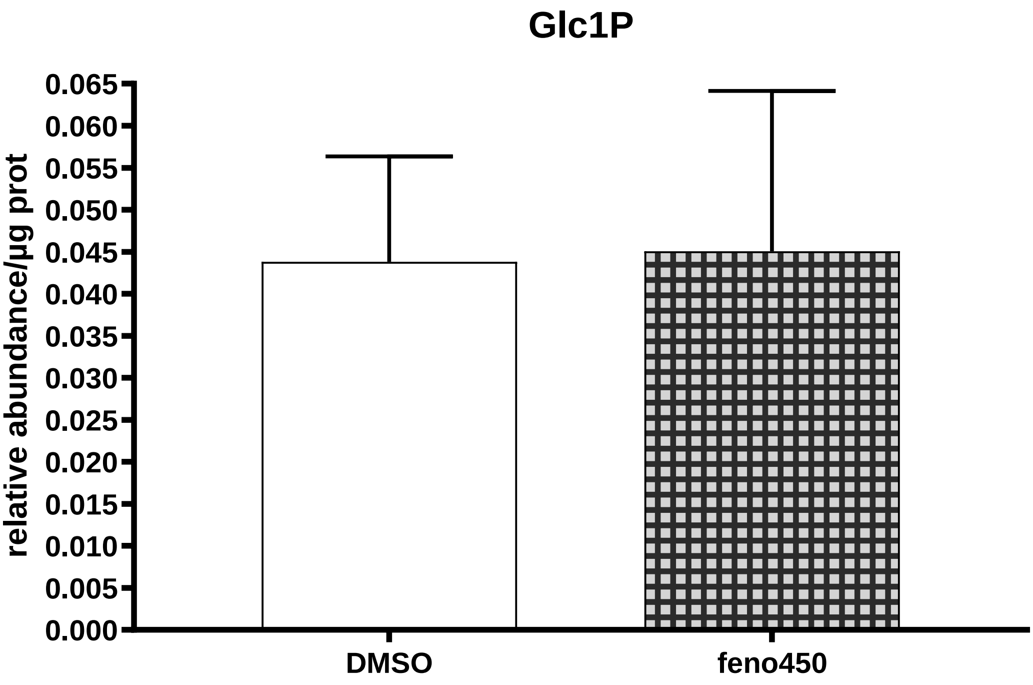
Glc1P
relative abundance/µg prot
0.065
0.060
0.055
0.050
0.045
0.040
0.035
0.030
0.025
0.020
0.015
0.010
0.005
0.000
DMSO
feno450

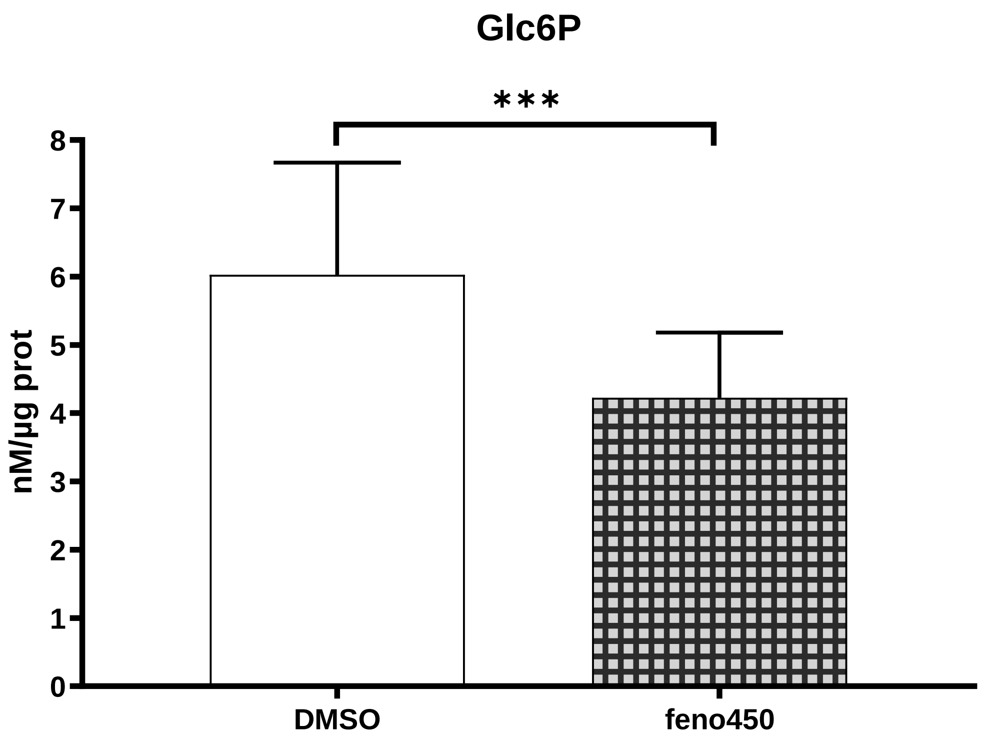
Glc6P
***
8
7
6
5
4
3
2
1
0
nM/µg prot
DMSO
feno450

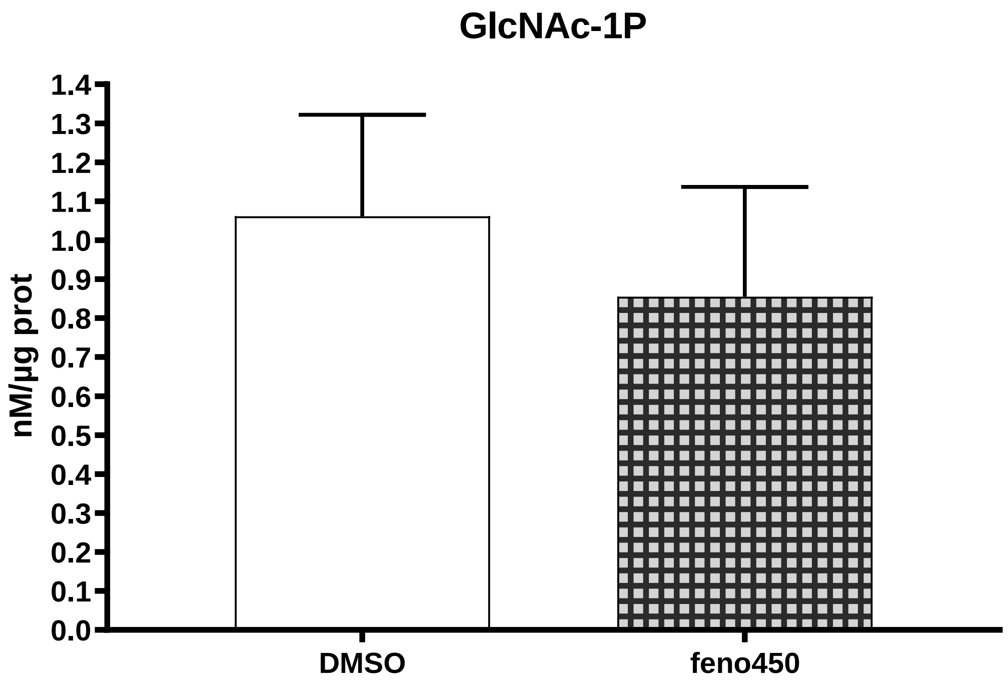
GlcNAc-1P
1.4
1.3
1.2
1.1
1.0
0.9
0.8
0.7
0.6
0.5
0.4
0.3
0.2
0.1
0.0
nM/µg prot
DMSO
feno450

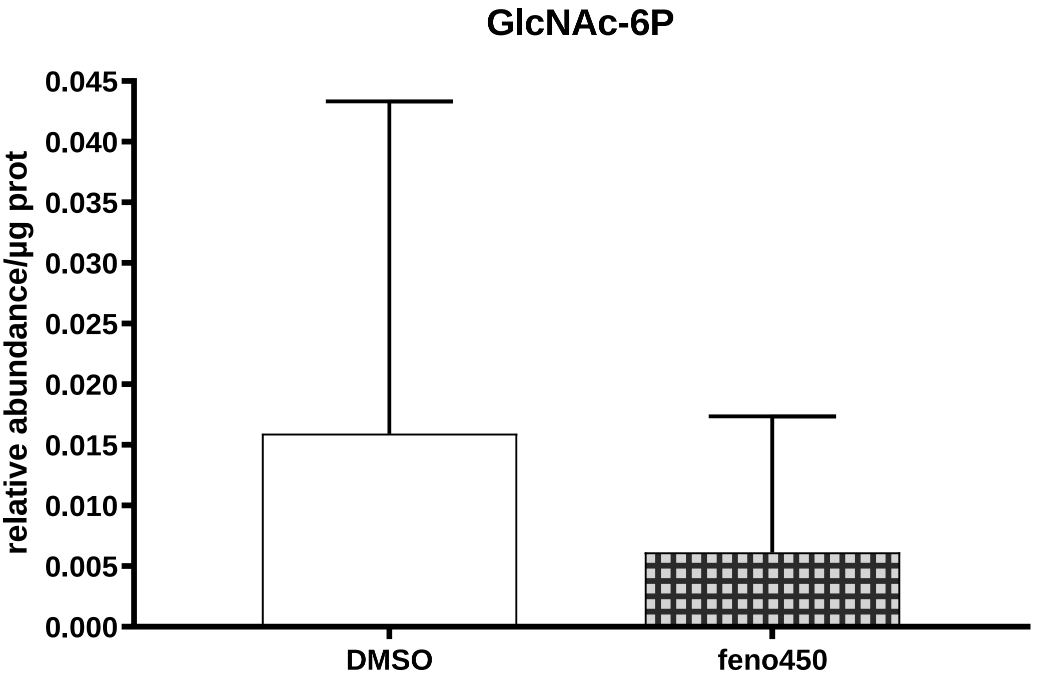
GlcNAc-6P
relative abundance/µg prot
0.045
0.040
0.035
0.030
0.025
0.020
0.015
0.010
0.005
0.000
DMSO
feno450

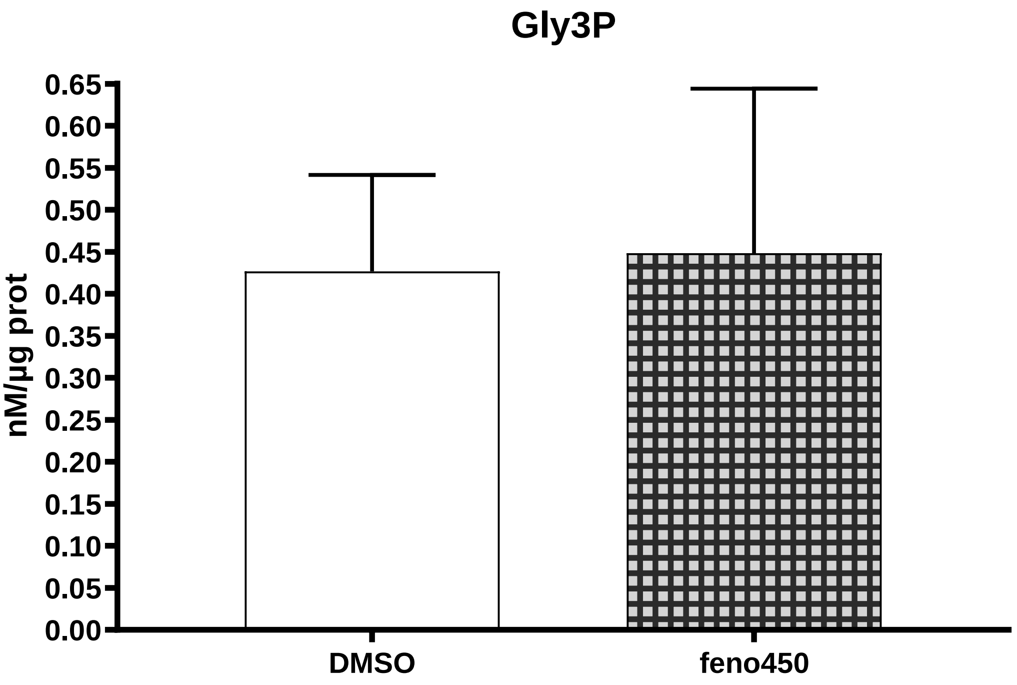
Gly3P
nM/µg prot
0.65
0.60
0.55
0.50
0.45
0.40
0.35
0.30
0.25
0.20
0.15
0.10
0.05
0.00
DMSO
feno450

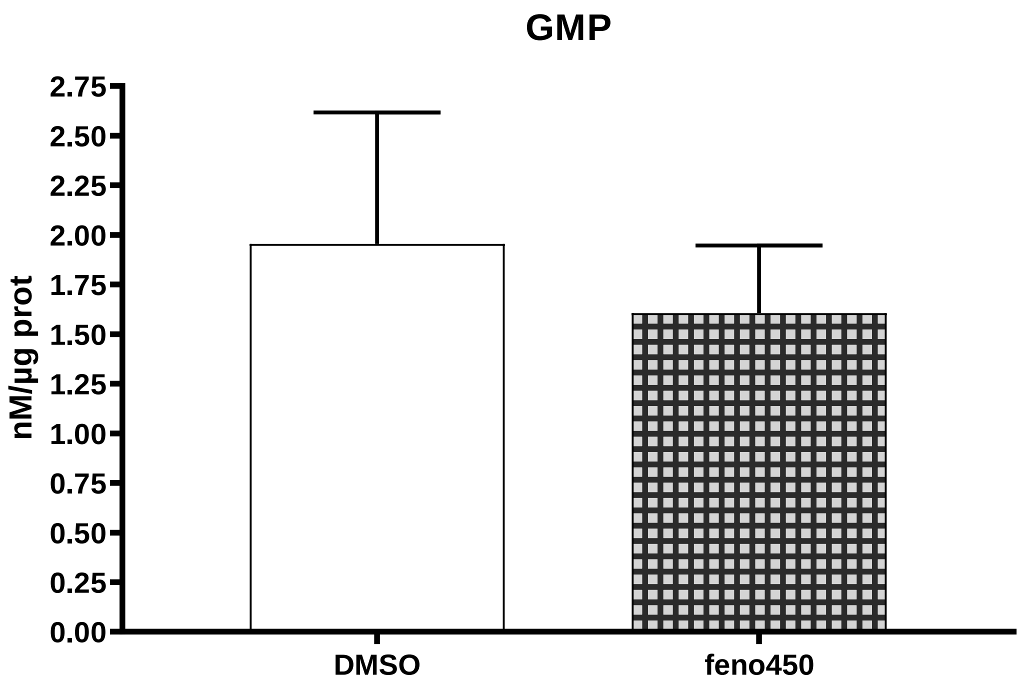
GMP
nM/µg prot
2.75
2.50
2.25
2.00
1.75
1.50
1.25
1.00
0.75
0.50
0.25
0.00
DMSO
feno450

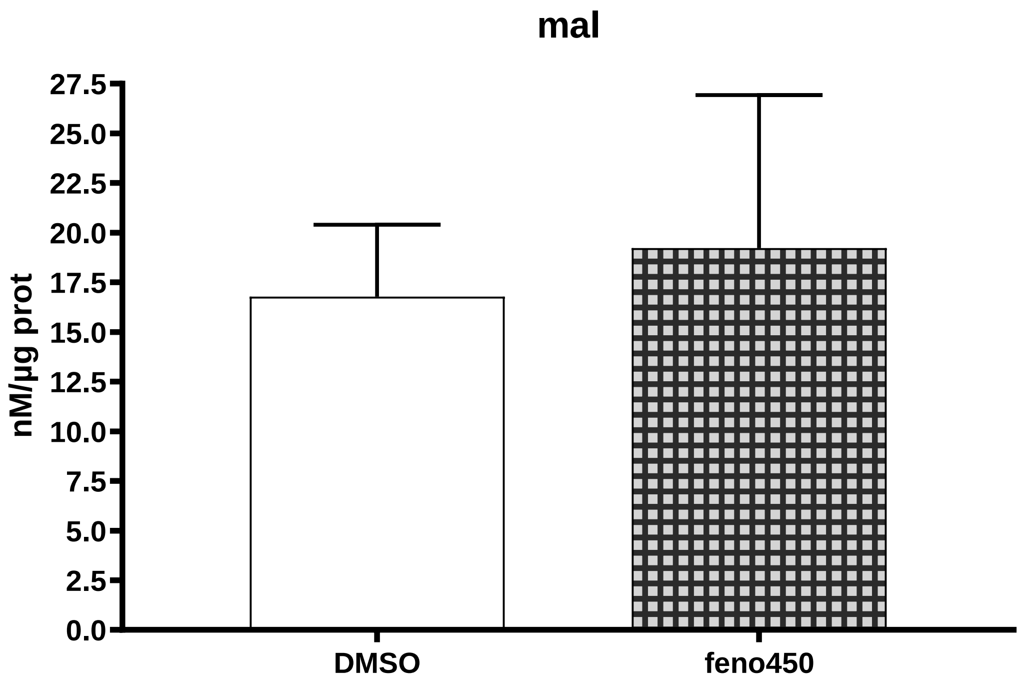
mal
nM/µg prot
27.5
25.0
22.5
20.0
17.5
15.0
12.5
10.0
7.5
5.0
2.5
0.0
DMSO
feno450

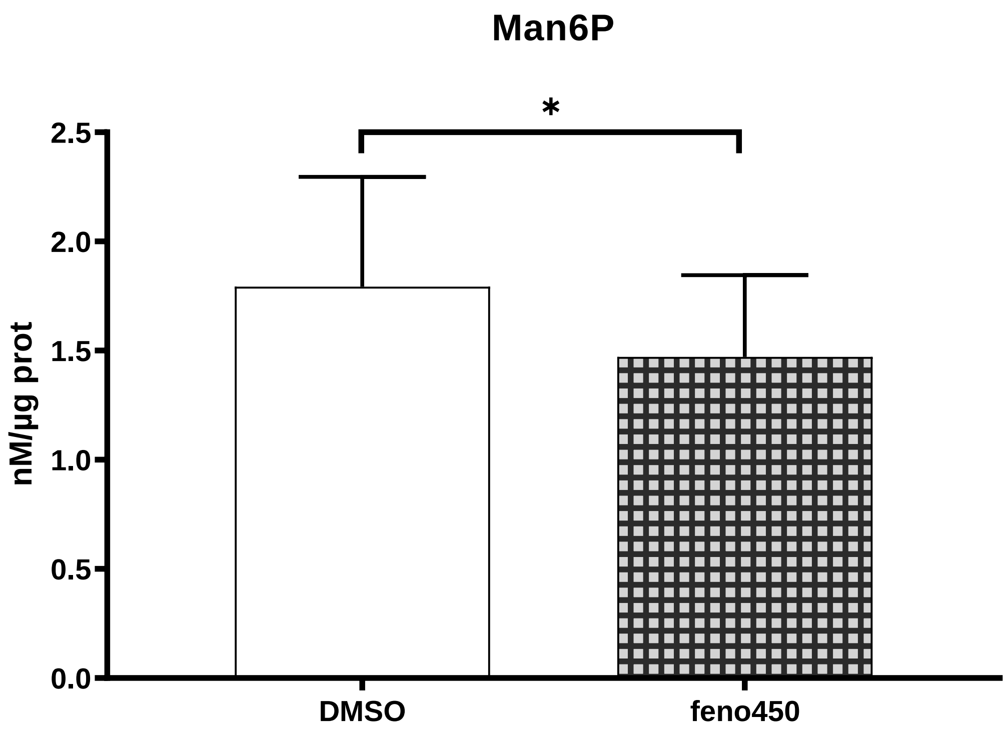
Man6P
*
2.5
2.0
1.5
1.0
0.5
0.0
nM/µg prot
DMSO
feno450

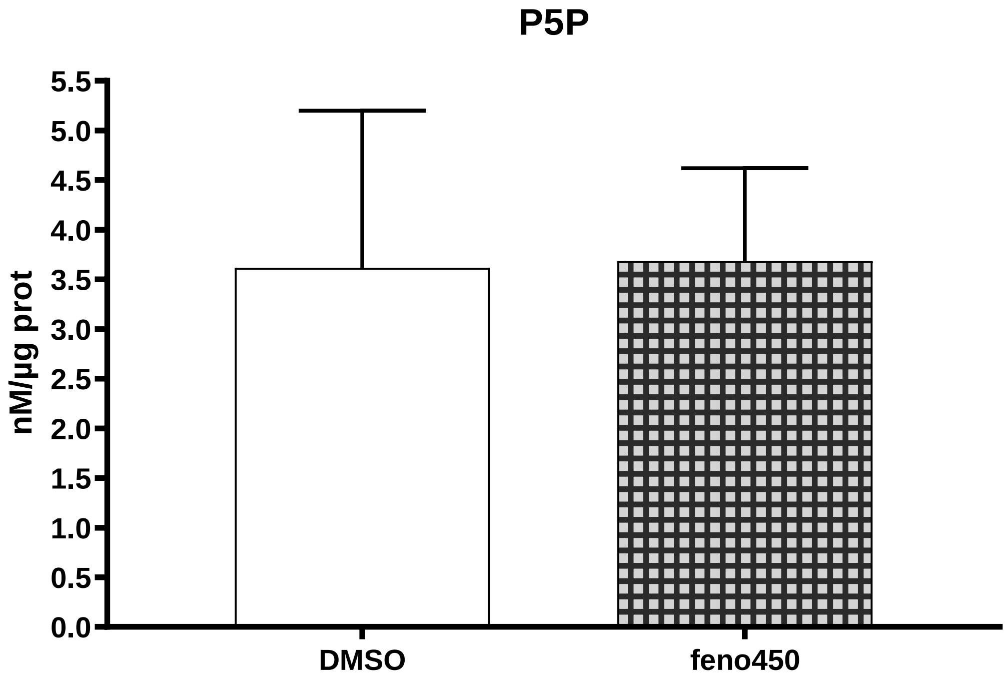
P5P
5.5
5.0
4.5
4.0
3.5
3.0
2.5
2.0
1.5
1.0
0.5
0.0
nM/µg prot
DMSO
feno450

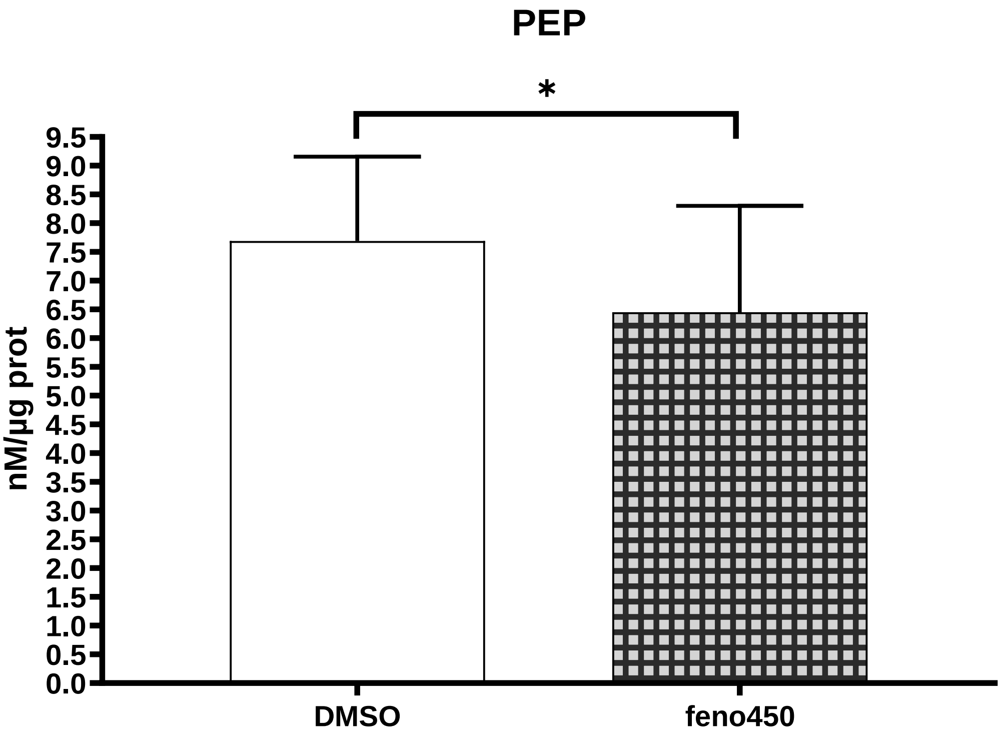
PEP
*
nM/µg prot
9.5
9.0
8.5
8.0
7.5
7.0
6.5
6.0
5.5
5.0
4.5
4.0
3.5
3.0
2.5
2.0
1.5
1.0
0.5
0.0
DMSO
feno450

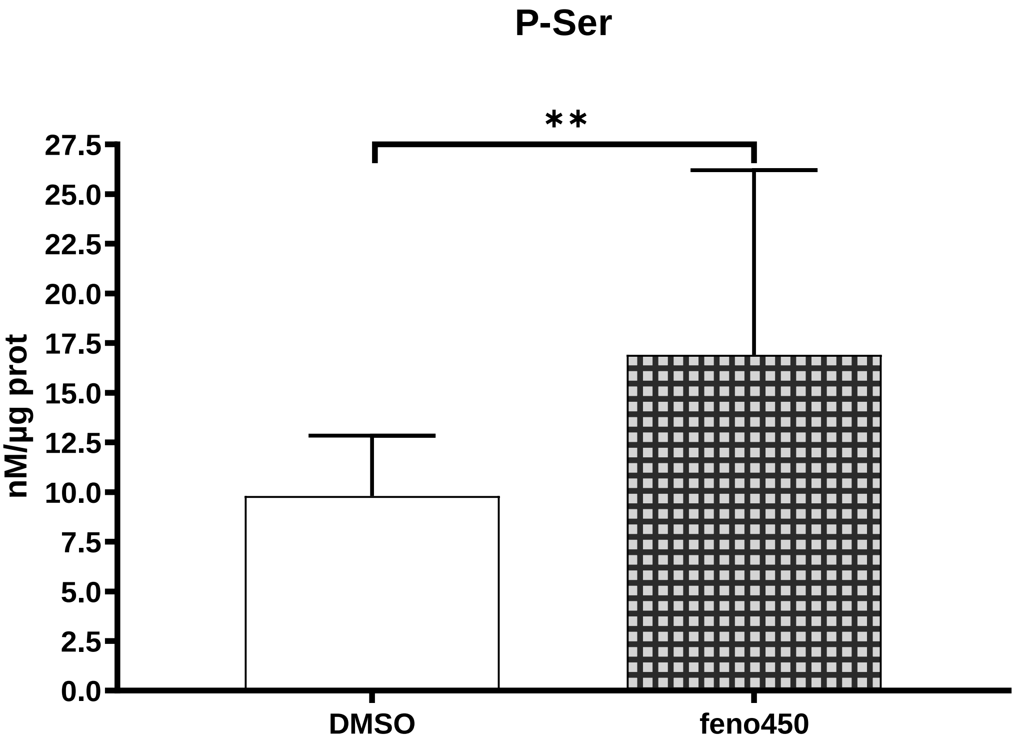
P-Ser
**
nM/µg prot
27.5
25.0
22.5
20.0
17.5
15.0
12.5
10.0
7.5
5.0
2.5
0.0
DMSO
feno450

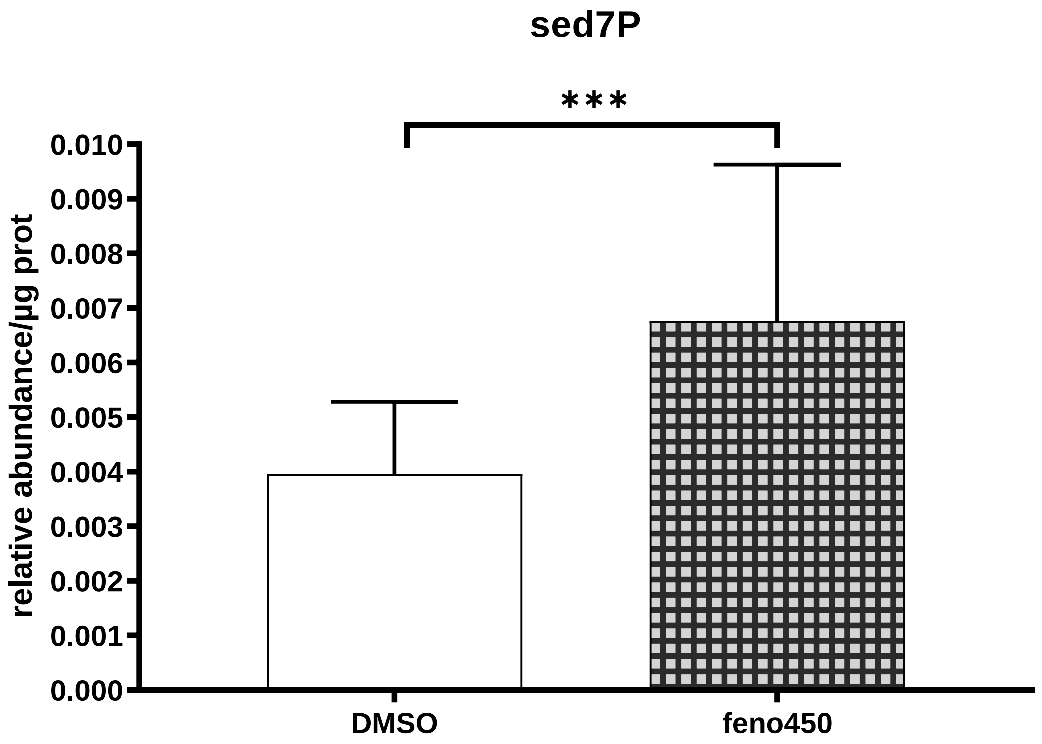
sed7P
***
relative abundance/µg prot
0.010
0.009
0.008
0.007
0.006
0.005
0.004
0.003
0.002
0.001
0.000
DMSO
feno450

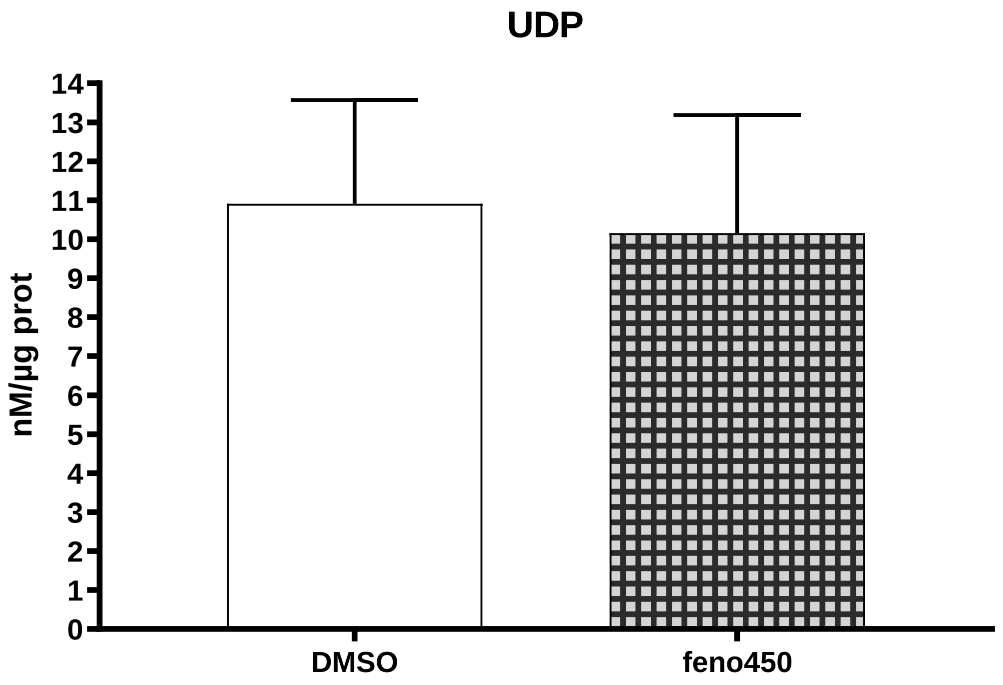
UDP
nM/µg prot
14
13
12
11
10
9
8
7
6
5
4
3
2
1
0
DMSO
feno450

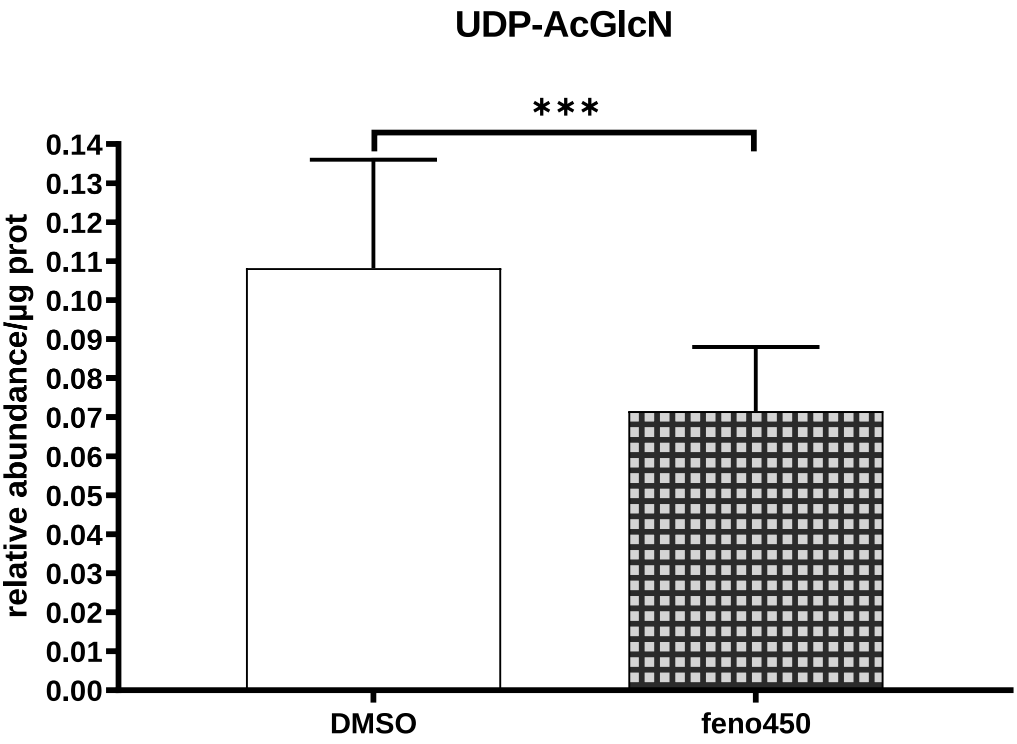
UDP-AcGlcN
***
relative abundance/µg prot
0.14
0.13
0.12
0.11
0.10
0.09
0.08
0.07
0.06
0.05
0.04
0.03
0.02
0.01
0.00
DMSO
feno450

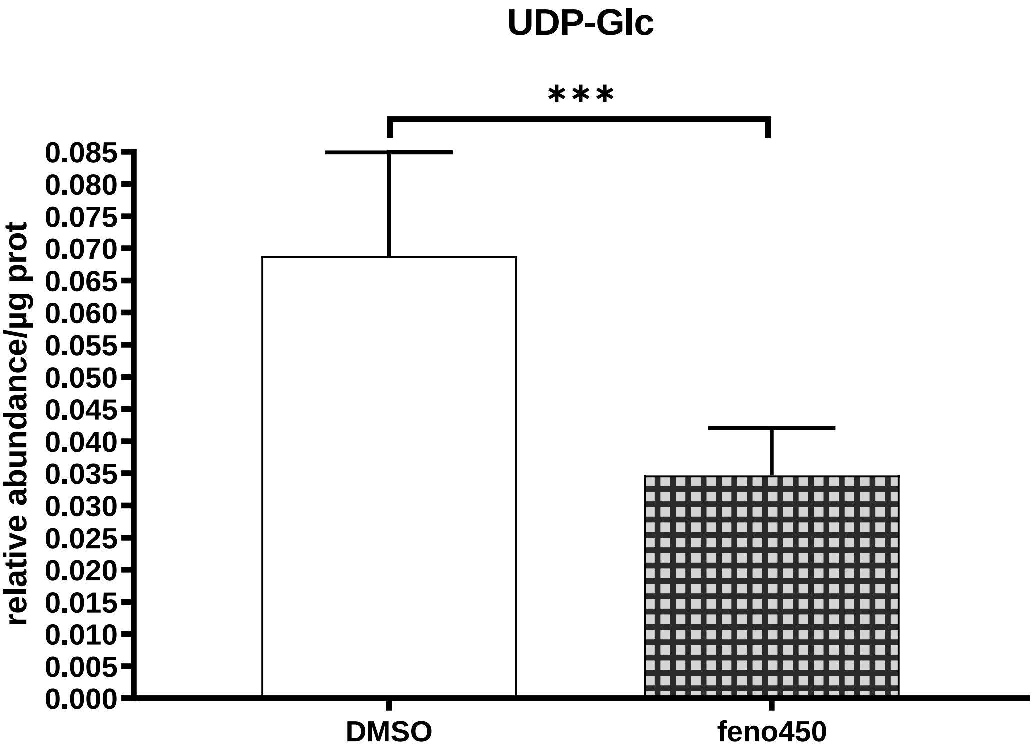
UDP-Glc
***
relative abundance/µg prot
0.085
0.080
0.075
0.070
0.065
0.060
0.055
0.050
0.045
0.040
0.035
0.030
0.025
0.020
0.015
0.010
0.005
0.000
DMSO
feno450

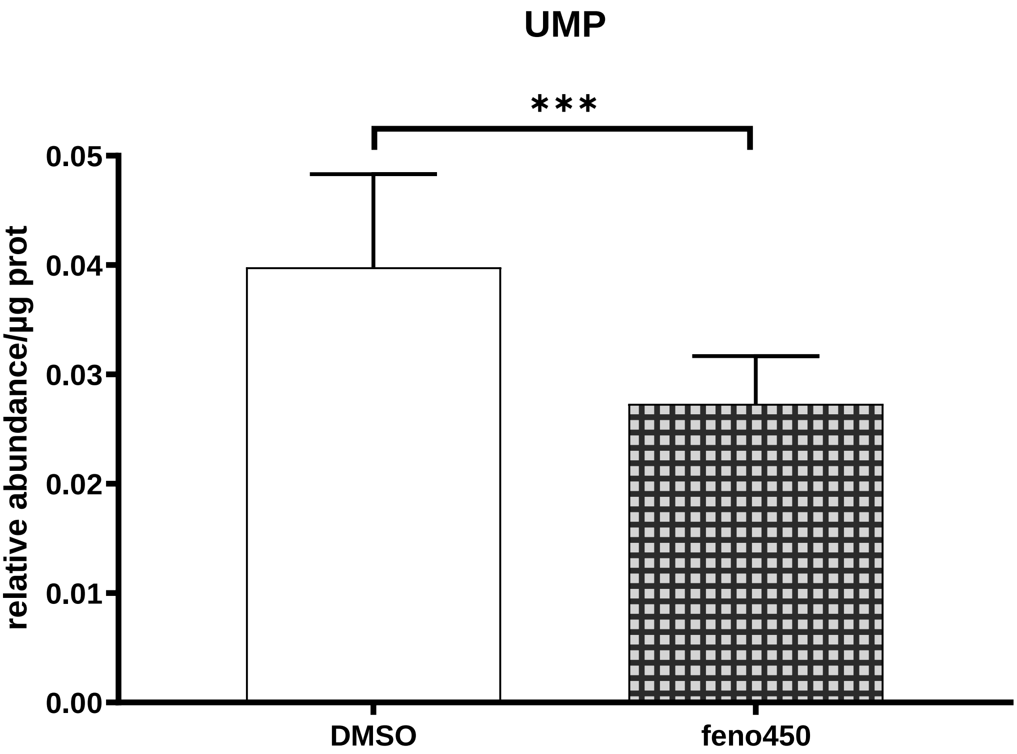
UMP
***
relative abundance/µg prot
0.05
0.04
0.03
0.02
0.01
0.00
DMSO
feno450

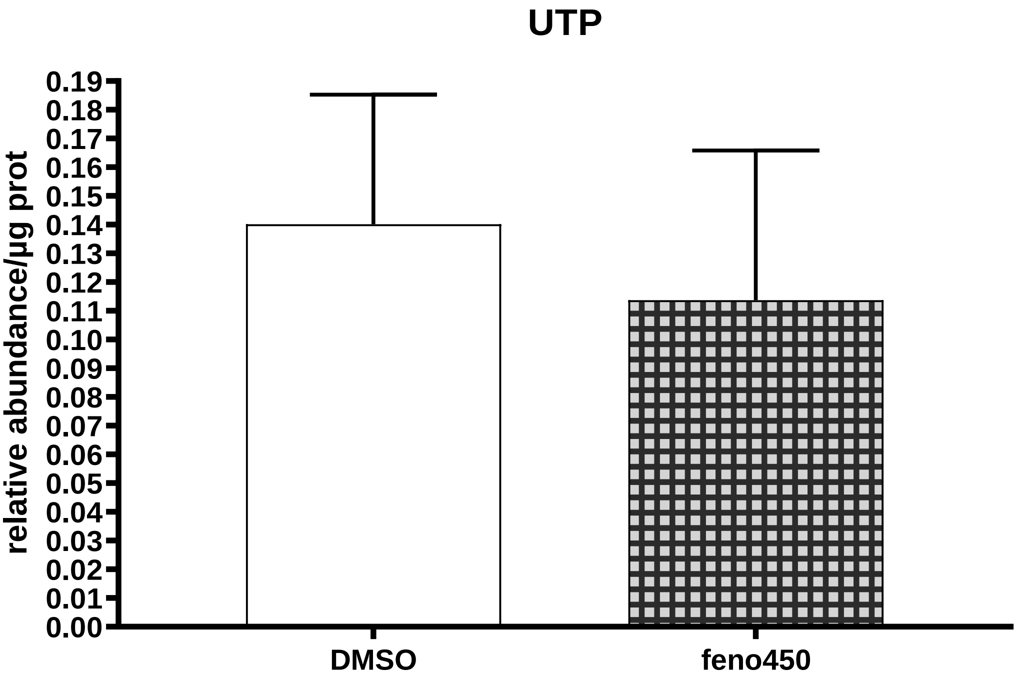

Figure S3. Effect of a high concentration of fenofibrate (450 µM) on HepaRG® cells. Data were obtained with 18 replicates. Amino acids, energy metabolites were quantified using 12C/13C ratio. Concentrations were obtained in nM using an external calibration. Relative abundance was obtained using only 12C/13C ratios for polar metabolites and ratio with their corresponding unlabelled internal standard for lipids. Bars represent mean ± SD (n=5). T-tests were performed for each metabolite (* p < 0.05; ** p < 0.01; *** p < 0.001). Triglycerides (TG), ceramides (Cer), phosphatidylcholines (PC), phosphatidylethanolamines (PE), sphingomyelins (SM), phosphatidylinositols (PI), alanine (Ala), aspartate (Asp), glutamine (Gln), glutamate (Glu) , glycine (Gly), leucine (Leu), isoleucine (Ile), serine (Ser), threonine (Thr), tryptophan (Trp), phenylalanine (Phe), arginine (Arg), histidine (his), asparagine (Asn), lysine (Lys), methionine (Met), proline (Pro), tyrosine (Tyr), valine (Val), 2,3-bisphosphoglycerate (2_3PG), 6_phosphogluconate (6-PG), a-ketoglutarate (a-KG), adenosine 5'-monophosphate (AMP), cytidine diphosphate (CDP), cytidine 5'-monophosphate (CMP), N-Acetylglucosamine-1-phosphate (GlucNac-1P), N-Acetylglucosamine-6-phosphate (GlucNac-6P), fructose-6-phosphate (Fru6P), fructose-1-phosphate (Fru1P), fructose bisphosphate (FruBP) guanosine diphosphate (GDP), guanosine diphosphate mannose (GDP-Man), glucose-6-phosphate (Glc6P), glucose-1-phosphate (Glc1P), glycerol-3-Phosphate (Gly3P), guanosine 5'-Monophosphate (GMP); mannose-6-phosphate (Man6P), pyridoxal-5-phosphate (P5P), phosphoenolpyruvate (PEP), uridine diphosphate (UDP), adenosine diphosphate (ADP), adenosine triphosphate (ATP), cytidine triphosphate (CTP), phosphoserine (P-Ser), sedoheptulose-7-phosphate (sed-7P), uridine diphosphate acetylglucosamine (UDP-AcGlcN), uridine diphosphate glucose (UDP-Glc), uridine 5'-monophosphate (UMP), uridine triphosphate (UTP).
